# Chemoproteomic profiling of kinases in live cells using electrophilic sulfonyl triazole probes†

**DOI:** 10.1039/d0sc06623k

**Published:** 2021-01-21

**Authors:** Tao Huang, Seyyedmohsen Hosseinibarkooie, Adam L. Borne, Mitchell E. Granade, Jeffrey W. Brulet, Thurl E. Harris, Heather A. Ferris, Ku-Lung Hsu

**Affiliations:** Department of Chemistry, University of Virginia McCormick Road, P.O. Box 400319 Charlottesville Virginia 22904 USA kenhsu@virginia.edu +1-434-297-4864; Department of Pharmacology, University of Virginia School of Medicine Charlottesville Virginia 22908 USA; University of Virginia Cancer Center, University of Virginia Charlottesville VA 22903 USA; Department of Molecular Physiology and Biological Physics, University of Virginia Charlottesville Virginia 22908 USA; Department of Medicine, University of Virginia School of Medicine Charlottesville Virginia 22903 USA

## Abstract

Sulfonyl-triazoles are a new class of electrophiles that mediate covalent reaction with tyrosine residues on proteins through sulfur-triazole exchange (SuTEx) chemistry. Recent studies demonstrate the broad utility and tunability of SuTEx chemistry for chemical proteomics and protein ligand discovery. Here, we present a strategy for mapping protein interaction networks of structurally complex binding elements using functionalized SuTEx probes. We show that the triazole leaving group (LG) can serve as a releasable linker for embedding hydrophobic fragments to direct molecular recognition while permitting efficient proteome-wide identification of binding sites in live cells. We synthesized a series of SuTEx probes functionalized with a lipid kinase fragment binder for discovery of ligandable tyrosines residing in catalytic and regulatory domains of protein and metabolic kinases in live cells. We performed competition studies with kinase inhibitors and substrates to demonstrate that probe binding is occurring in an activity-dependent manner. Our functional studies led to discovery of probe-modified sites within the C2 domain that were important for downregulation of protein kinase C-alpha in response to phorbol ester activation. Our proof of concept studies highlight the triazole LG of SuTEx probes as a traceless linker for locating protein binding sites targeted by complex recognition elements in live cells.

## Abstract 2

Sulfonyl-triazole probes modified with a kinase recognition element are developed for live cell activity-based profiling to identify tyrosine sites located in catalytic and regulatory domains that are important for kinase function.

## Graphical abstract

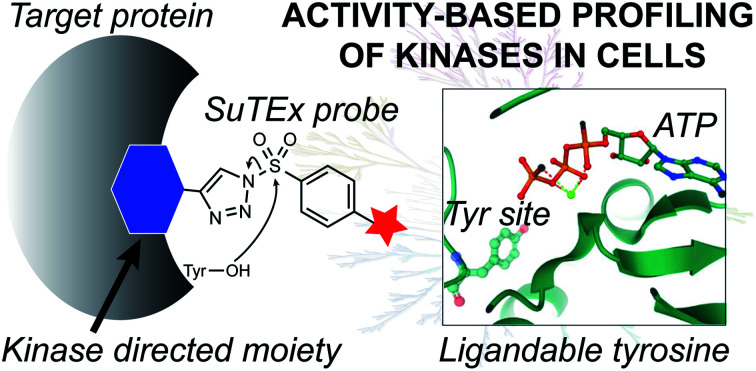

## Introduction

Covalent probes are important tools for studying protein biology and for advancing translational discoveries.^1,2^ Fragments and functionalized inhibitor molecules bearing electrophiles or photoreactive groups have been used for proteomic discovery of ligand sites that can be targeted for pharmacological control^3–5^ and protein degradation.^6,7^ A suite of chemistries for investigating cysteine-,^5,8–10^ lysine-,^11–14^ aspartate/glutamate-,^15,16^ and tyrosine-^17–22^ residues is providing new opportunities to develop ligands for perturbing protein function.^4–7,11,23^ Covalent probes have also provided creative solutions for studying post-translational modifications (PTM) including phosphorylation,^17^ methylation,^24^ crotonolyation,^25^ and deimination.^26^

We introduced sulfur-triazole exchange (SuTEx) chemistry as a new class of electrophiles for chemical proteomic applications^17^ and fragment-based ligand discovery^27^ (FBLD). The SuTEx reaction occurs through nucleophilic attack of the sulfur center to facilitate protein reaction preferentially on tyrosine residues in lysates and live cells.^17,27^ In contrast with the fluoride leaving group (LG) on SuFEx,^28,29^ the addition of a triazolide LG on SuTEx molecules introduced additional opportunities for tuning activity of the sulfur electrophile. We recently expanded our reactivity studies to investigate adduct group (AG) and LG modifications for activating nucleophilic substitution reactions at the sulfur center. We applied the reactivity principles gained from our FBLD studies to demonstrate the tunability of SuTEx for developing ligands to disrupt functional tyrosine sites on proteins.^27^

**Fig. 1:**
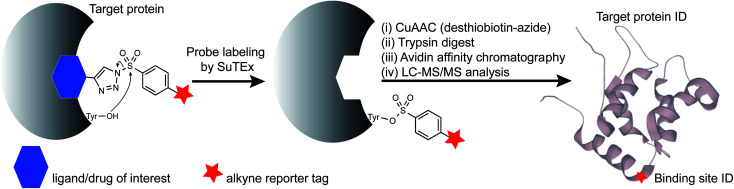
SuTEx-enabled identification of binding sites of ligands/drugs of interest. Embedding the triazolide leaving group with recognition elements (ligands/drugs) can direct binding specificity of alkynyl-SuTEx probes for LC-MS chemical proteomic evaluation. After covalent reaction, the ligand/drug-functionalized triazole group is absent from probe-modified sites to simplify site of binding experiments.

Ligandable tyrosine sites could also be discovered by functionalizing existing fragments with the SuTEx electrophile and bioorthogonal reporter groups. The triazole group is well positioned for incorporating recognition elements because of its synthetic accessibility and absence from modified binding sites after covalent reaction to simplify chemical proteomic investigations (Fig. 1). The latter feature is especially important for determining the site of binding using fragment binders of medium to high structural complexity, which typically produce covalent probe-peptide adducts that are difficult to identify by liquid chromatography-mass spectrometry (LC-MS). To circumvent these issues, specialized proteomic workflows have been developed, for example, to understand LC-MS fragmentation mechanisms and increase confidence in binding site identifications of cysteine-directed covalent drugs.^30,31^ Given the complexities of LC-MS compound fragmentation mechanisms, these approaches, while effective, will likely need to be customized for each covalent probe analyzed.

Here, we present an alternative strategy for mapping protein interactions of structurally complex binding elements. We used a hydrophobic fragment (RF001) derived from the serotonin receptor antagonist ritanserin to determine whether its secondary activity against kinases *in vitro* was reflected in live cells. We synthesized a series of RF001-functionalized SuTEx probes to discover modified tyrosines on known targets of ritanserin including sites important for biochemical function of diacylglycerol kinase-alpha and inhibitor binding of the non-receptor tyrosine kinase FER. We applied RF001-SuTEx probes to profile inhibitor and substrate binding across a suite of ligandable tyrosine sites located in catalytic and regulatory domains of >50 native kinases in live T cells to discover regions of the C2 domain important for downregulating protein kinase C-alpha during prolonged cellular activation.

## Results and discussion

### Synthesis of RF001-functionalized SuTEx probes

RF001 is a fragment derived from the serotonin receptor antagonist ritanserin that was tested in the clinic for treatment of psychiatric disorders.^32^ Further investigations into ritanserin activity revealed secondary activity against DGKα^33^ and other kinases.^34^ RF001 showed improved specificity for DGKα compared with ritanserin as determined by activity-based profiling studies using ATP acyl phosphate probes.^35^ The proteome-wide activity of RF001, however, is currently unknown and is important for evaluating whether RF001 is a suitable fragment for developing kinase probes and inhibitors.

**Scheme 1:**
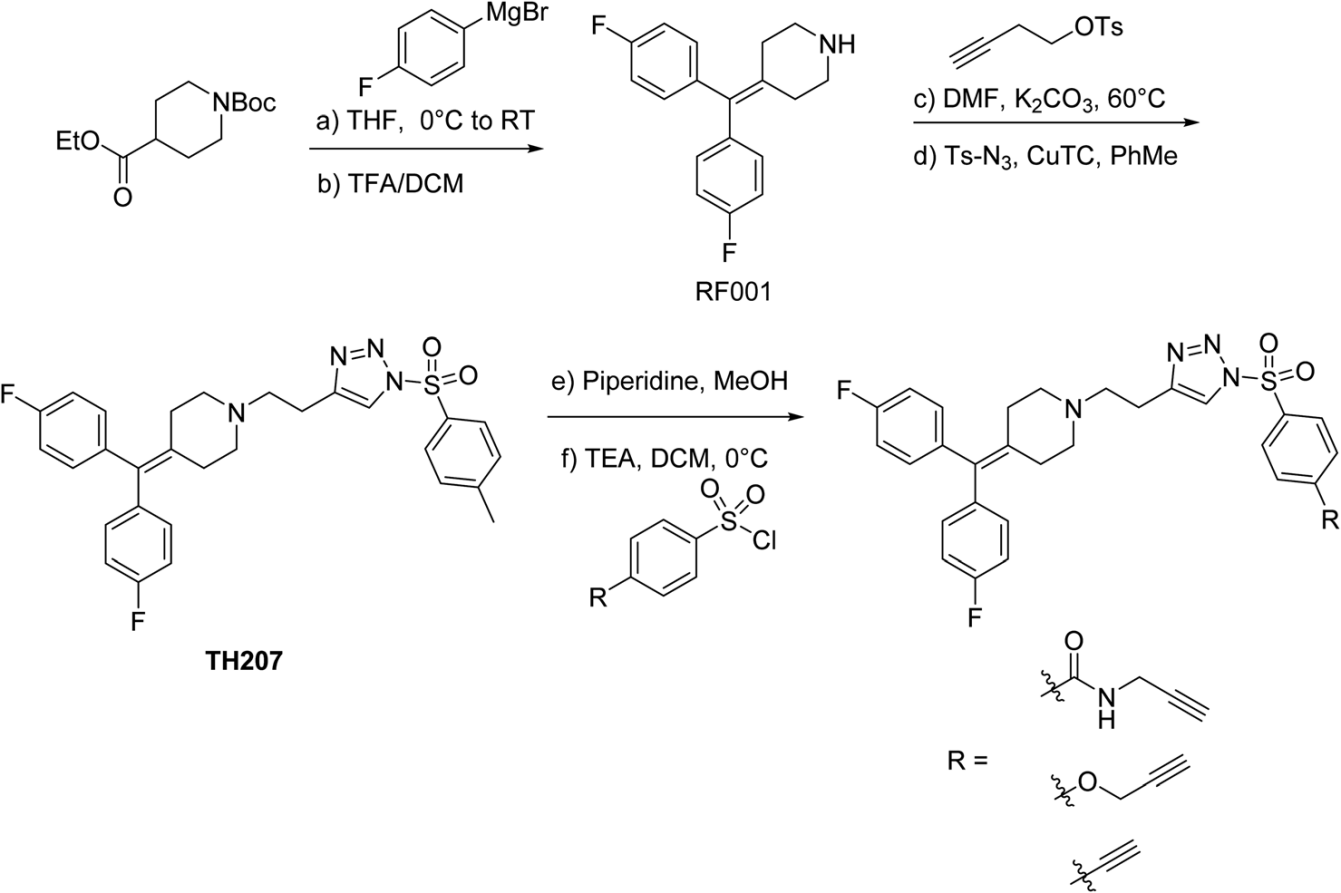
Synthesis of SuTEx probes. Reagents and conditions: (a) 4-fluorophenylmagnesium bromide (2.2 equiv.), THF, 0 °C to RT, 14 h; (b) 50% TFA in DCM, RT, 14 h, 80% over two steps; (c) but-3-yn-1-yl tosylate (1.2 equiv.), K_2_CO_3_ (5 equiv.), DMF, 60 °C, 6 h, 85%; (d) Ts-N_3_ (1.2 equiv.), CuTC (20 mol%), PhMe, RT, 4 h, 50%; (e) piperidine (5 equiv.), MeOH, RT, 0.5 h, quant.; (f) sulfonyl chloride (1.2 equiv.), TEA (2 equiv.), DCM, 0 °C to RT, 14 h, 35–60%. Ts-N_3_, tosyl azide; CuTC, copper(i) thiophene-2-carboxylate.

We modified the triazole group with the RF001 recognition element to produce functionalized SuTEx probes for evaluation of proteome activity. We chose to incorporate the RF001 moiety into the LG and not the AG for our probe design to avoid generating bulky probe-peptide adducts that could complicate LC-MS site-of-binding studies. We developed a synthetic route to incorporate RF001 as a recognition element for functionalized SuTEx probe design. Specifically, ethyl *N*-Boc-piperidine-4-carboxylate was reacted with two equivalents of 4-fluorophenyl magnesium bromide to produce an intermediate that was dehydrated and deprotected with TFA in one pot to form the RF001 product (Scheme 1). RF001 was synthesized with excellent yield (>80%) without the need for additional column chromatography. A terminal alkyne was installed through alkylation of the piperidine nitrogen. Compound TH207 was synthesized following a copper(i) thiophene-2-carboxylate (CuTC)-mediated coupling^36^ between the RF001-alkyne intermediate and tosyl azide with moderate yield (50%). Next, the tosyl group was removed and the resulting triazole intermediate coupled to various alkyne-containing sulfonyl chlorides to produce the corresponding SuTEx probes. See ESI† for synthetic procedures and probe characterization.

### Adduct group modifications tune reactivity of RF001-SuTEx probes in proteomes

**Fig. 2:**
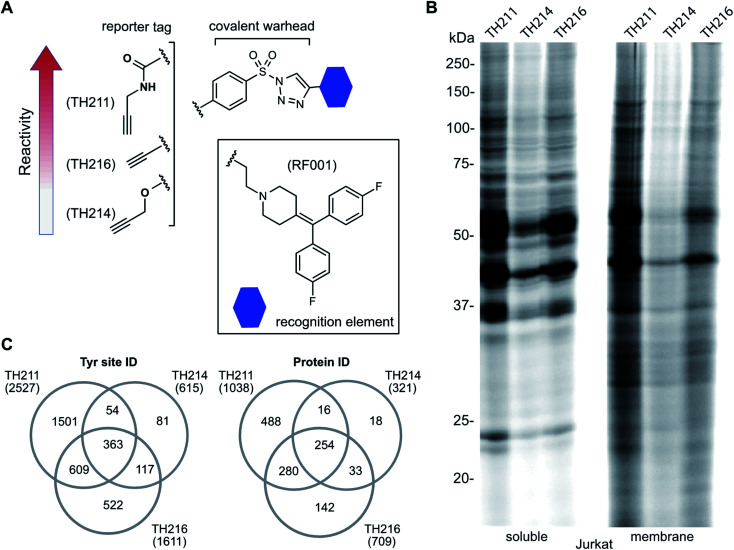
Tuning reactivity of RF001-SuTEx probes. (A) RF001-SuTEx probes contain a common triazole binding element but differ in whether the alkyne reporter is conjugated to the AG through a carbonyl (TH211), alkoxy (TH214), or direct (TH216) linkage. (B) Gel-based chemical proteomic evaluation of Jurkat cell proteomes treated with RF001-SuTEx probes (100 μM, 1 h, 37 °C) shows that AG modifications affect proteome reactivity of probes. (C) Quantitative LC-MS chemical proteomic evaluation of SILAC light and heavy HEK293T soluble proteomes (overexpressing human DGKα) treated with RF001-SuTEx probes (100 μM, 1 h, 37 °C) shows the number of probe-modified tyrosines and proteins, which reflects the reactivity of corresponding probes. Only probe-modified sites that are specifically enriched (light – probe/heavy – vehicle SILAC ratios >5) and meet our peptide quality control criteria are included. See ESI† for additional details of LC-MS studies and data analysis. A complete list of SuTEx probe-modified sites and proteins from *in vitro* probe labeling studies can be found in Table S1.† All data shown are representative of 3 experiments (*n* = 3 biologically independent experiments).

We used a gel-based chemical proteomic assay to evaluate reactivity of RF001-SuTEx probes. Each SuTEx probe shared a common RF001-modified triazole group but differed by the chemical linker for attachment of the alkyne reporter tag. The alkyne group was incorporated into the AG structure through an amide (TH211), methoxy (TH214), or direct conjugation (TH216) to the phenyl of the sulfonyl group (Fig. 2A). Considering the sensitivity of SuTEx reaction to AG modifications,^27^ we reasoned that these structurally analogous probes would show differences in proteome reactivity and provide further insights into electronic effects for tuning activity of the sulfur electrophile.

To compare proteome activity of RF001-SuTEx probes, Jurkat soluble and membrane proteomes were treated with TH211, TH214, or TH216 (100 μM, 1 h, 37 °C) followed by copper-catalyzed azide–alkyne cycloaddition (CuAAC^37^) conjugation of rhodamine-azide to visualize probe-labeled protein targets resolved by SDS-PAGE and detected by in-gel fluorescence scanning. Our gel-based experiments identified TH211 as the most reactive probe as evidenced by robust fluorescence labeling of proteins across the entire molecular weight range (Fig. 2B). These findings are in agreement with our previous studies that demonstrated modifications of the AG with electron-withdrawing groups (*e.g.* the carbonyl of TH211) can enhance reactivity of SuTEx reaction in solution and proteomes.^27^ This reactivity profile was also supported by differences in activity for TH214 and TH216. Although both probes showed reduced labeling compared with TH211, the electron donating character of the alkoxy group^38^ of TH214 could help explain the decrease in reactivity compared with TH216 (Fig. 2B). Gel-based analyses of TH211-, TH214- and TH216-treated HEK293T proteomes yielded reactivity profiles similar to those observed with Jurkat proteomes (Fig. S1†).

In summary, we utilized gel-based chemical proteomics to compare reactivity of SuTEx probes bearing a common RF001-modified LG and differentiated by the reporter tag conjugation to the AG. We demonstrate that changing the chemical connectivity of the alkyne tag on the AG can profoundly affect proteome reactivity of resulting RF001-SuTEx probes.

### RF001 binding element directs molecular recognition of SuTEx probes in proteomes

Next, we deployed quantitative chemical proteomics^17,27^ to evaluate proteome-wide activity of RF001-SuTEx probes. We performed chemical proteomic studies using recombinant DGKα overexpressed lysates for proof of concept that the RF001 binding element was directing molecular recognition. In support of RF001-mediated binding, we observed concentration-dependent labeling of an ∼80 kDa fluorescent TH211-labeled band in recombinant FLAG-tagged rat DGKα (rDGKα) overexpressed but not mock transfected HEK293T membrane and soluble proteomes (Fig. S2†). Next, we analyzed isotopically light and heavy amino acid-labeled recombinant DGKα-HEK293T proteomes to enable quantitative LC-MS by SILAC.^39^ Here, we focused our efforts on human DGKα (hDGKα) because the RF001 binding sites against the human protein have not yet been identified. In brief, recombinant light and heavy hDGKα-HEK293T cell proteomes were treated with RF001-SuTEx probes (100 μM, 1 h, 37 °C) or dimethyl sulfoxide (DMSO) vehicle, respectively, followed by CuAAC^37^ coupling with a desthiobiotin-azide tag. Proteomes were digested with trypsin protease and desthiobiotin-modified peptides enriched by avidin affinity chromatography, released, and analyzed by high-resolution LC-MS as previously described^17,27^ and depicted in Fig. 1.

Probe-modified peptide-spectrum matches (PSMs) that met our quality control confidence criteria of ≥300 Byonic score,^40^ 1% protein false discovery rate (FDR), and ≤5 ppm mass accuracy were selected for further evaluation to minimize false positives.^17^ SILAC ratios (SR) from these chemical proteomic studies (light – SuTEx probe/heavy – DMSO vehicle) were used to identify probe-modified peptides that were substantially enriched in probe- *versus* vehicle-treated samples (SR >5). We compared reactivity profiles of each respective RF001-SuTEx probe across >3000 distinct probe-modified sites from ∼1200 detected proteins (Fig. 2C). In agreement with our gel-based results (Fig. 2B and S1†), we observed an approximate 2–4-fold higher number of TH211-compared with TH214- or TH216-modified tyrosine sites (∼2–4-fold higher number of modified sites, Fig. 2C). The difference in reactivity between TH214 and TH216 was also recapitulated in our quantitative proteomic experiments with the latter SuTEx probe showing a >2-fold enhanced reactivity in the number of modified sites and proteins (Fig. 2C). A complete list of *in vitro* RF001-SuTEx probe-modified sites can be found in Table S1.†

We showed in previous studies that RF001 engages the rDGKα active site through interactions with the catalytic domain^35^ and C1 lipid recognition domain.^41^ Here, we reasoned that probe-modified binding sites identified consistently across all 3 RF001-SuTEx probes would represent hDGKα active site regions that have a high propensity for ligand binding. In support of our hypothesis, we identified probe-modified sites in several domains, which we previously identified as ligand-binding regions of the rat DGKα active site using ATP acyl phosphate activity-based probes of kinases.^35^ Specifically, we identified probe binding at the C1A (Y240, Y258), DAGKc (Y399, Y477), and DAGKa (Y623, Y669) domains of recombinant hDGKα that showed specific enrichment across all 3 RF001-SuTEx probes in our chemical proteomic studies (SR >5, Fig. S3†). Interestingly, we identified additional enriched peptides that contained modified tyrosines at the N-terminus within the RVH domain^42^ (Y19, Y22) and the second EF-hand domain of hDGKα (Y169, Fig. S3†).

In summary, we demonstrate that installment of a RF001 binding element on SuTEx probes can direct molecular recognition to the hDGKα active site and reveal ligandable tyrosine sites within the C1A and DAGKc/DAGKa domains. In contrast with ATP acyl phosphate probes used previously, the RF001-SuTEx probes identified novel binding sites within the RVH and EF-hand domains of DGKα. Future studies will focus on evaluating the biochemical and metabolic impact of perturbing these DGKα tyrosine sites.

### Live cell profiling of inhibitor binding interactions against native DGKα and FER

Next, we deployed quantitative chemical proteomics^17,27^ to map TH211 binding sites in live Jurkat T cells cultured in SILAC media. We treated Jurkat cells with varying concentrations of TH211 followed by gel-based chemical proteomic analysis to determine the optimal probe concentration for our quantitative LC-MS studies. We observed concentration-dependent probe labeling of proteomes from TH211-treated Jurkat cells and identified a saturating probe concentration for our LC-MS studies (50 μM TH211, Fig. S4†).

**Fig. 3:**
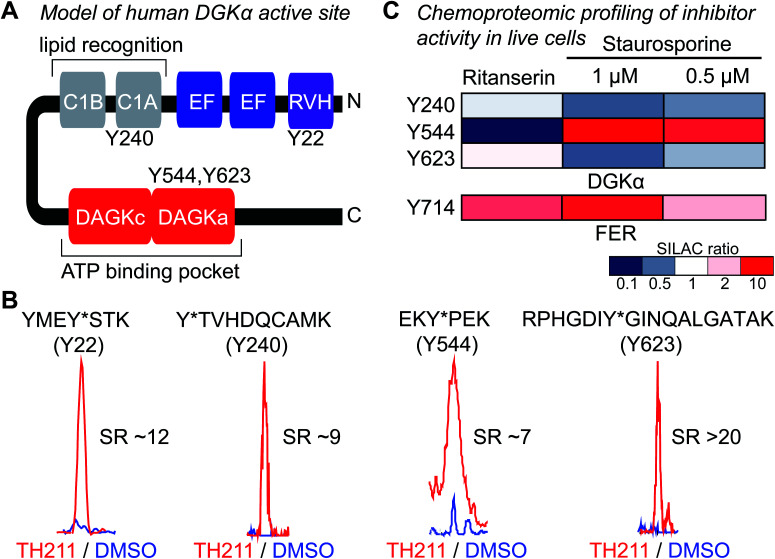
TH211 enables functional profiling of inhibitor binding against native DGKα and FER in live cells. (A) Working model of the DGKα active site composed of a lipid- (C1A) and ATP-binding (DAGKc/DAGKa) interdomain architecture. (B) TH211-modified tyrosine sites on native human DGKα were identified by treating light and heavy SILAC Jurkat cells with TH211 probe (50 μM) or DMSO vehicle, respectively, for 2 h at 37 °C followed by quantitative LC-MS chemical proteomics. Representative MS1 extracted ion chromatograms (EICs) of TH211-modified peptides containing tyrosine site of binding information from live cell probe labeling studies. Specific probe enrichment was confirmed by light – TH211 (red)/heavy – DMSO vehicle (blue) SILAC ratios (SR) >5. See ESI† for additional details of *in situ* TH211 studies and distribution of TH211-modified sites detected in soluble, membrane, or both fractions. (C) Inhibitory activity of ritanserin and staurosporine against native human DGKα and FER kinase probe-modified sites as quantified by chemical proteomics. SILAC light and heavy Jurkat cells were treated with DMSO vehicle or inhibitor (ritanserin – 25 μM or staurosporine – 1 and 0.5 μM), respectively, for 1 h followed by TH211 probe labeling (50 μM) for 2 h. Cells were lysed and subjected to LC-MS chemical proteomics as described in Methods. The degree of inhibition of probe labeling by compounds at respective sites was quantified by the SR of light to heavy MS1 peptide abundances. The heatmap depicts sensitivity of tyrosine sites on DGKα and FER to compound treatments in order to determine site of binding for inhibitors tested. A complete list of SuTEx probe-modified sites and proteins from live cells studies can be found in Table S1.† All data shown are representative of *n* = 2–3 biologically independent experiments.

Next, light and heavy SILAC Jurkat cells were treated with TH211 probe (50 μM, 2 h) or DMSO vehicle, respectively, followed by cell lysis and quantitative chemical proteomics by LC-MS (Fig. 1). Initially, we evaluated whether TH211 could mediate molecular recognition and binding to native hDGKα in live cells. These studies are important because current probes for DGKs are not suitable for direct activity-based profiling in live cells. We identified prominent TH211 labeling of hDGKα in regions that demonstrated specific enrichment (SR >5) of active site binding of TH211 in live cells (Fig. 3A). Specifically, we identified TH211 modified sites within the C1A (Y240) and accessory domain (Y544, Y623) of native hDGKα (Fig. 3B). We showed that the Y240F mutant is catalytically impaired compared with wild-type protein, which supports the ability of TH211 to bind functional sites important for hDGKα activity (Fig. S5†). Interestingly, the binding site profiles were dependent to some degree on the fraction analyzed; certain probe-modified peptides were detected preferentially in soluble, membrane, or both fractions (see ESI Methods for details†). Additional studies are needed to determine whether these differences in live cell probe labeling reflect differential regulation of hDGKα (*e.g.* autoinhibition) in cellular environments.

Having established the TH211 binding profile for hDGKα, we performed competition studies using reported DGKα inhibitors (ritanserin) and broad-spectrum kinase inhibitors (staurosporine) to determine whether TH211-hDGKα interactions *in situ* are functionally relevant. RF001 was not used as the competitor because this fragment compound requires millimolar concentrations^35^ for effective binding to target proteins and this high amount of compound is not suitable for live cell studies. Light and heavy SILAC Jurkat cells were treated with vehicle or inhibitor (ritanserin, 25 μM; staurosporine, 1 or 0.5 μM; 60 min at 37 °C) followed by TH211 probe labeling (50 μM, 2 h). Cells were lysed and processed for quantitative chemical proteomic analysis to evaluate target engagement of competitors at respective hDGKα binding sites. We observed mild competition (SR ∼2) at the C1 domain (Y240) and DAGKa catalytic domain (Y623) in ritanserin-treated cells, which is in agreement with previous observation of ritanserin activity against rat DGKα^35^ (Fig. 3C). The other TH211-modified sites of hDGKα did not appear to be inhibited by ritanserin treatments (Fig. 3C).

While ritanserin showed mild activity against hDGKα, we observed potent activity of this compound against FER kinase in live cells as determined by blockade of TH211 probe labeling at the Y714 site (SR ∼4, Fig. 3C). These findings are in agreement with our previous ATP acyl phosphate kinome profiling studies that identified FER as the principal kinase targeted by ritanserin.^35^ Treatment of Jurkat cells with the broad-spectrum kinase inhibitor staurosporine resulted in concentration-dependent blockade of TH211 probe labeling at FER Y714 (SR of 2 and 5 for 0.5 and 1 μM staurosporine, respectively; Fig. 3C). These findings are in agreement with previous reports that staurosporine is competitive for active site probe labeling of FER.^43^ Interestingly, we also observed competition at the Y544 site of hDGKα in Jurkat cells treated with staurosporine (Fig. 3C).

In summary, our chemical proteomic findings demonstrate the utility of SuTEx probes such as TH211 that exhibit broad reactivity and binding recognition for mapping protein targets directly in live cells (Fig. 3 and Table S1†). We identify ligand-binding sites on native hDGKα in domain regions that are in agreement with previous chemoproteomic evaluation of DGK active sites. We also demonstrate that TH211 can be used to evaluate inhibitor binding against lipid (DGKα) and protein (FER) kinases directly in live cells to support activity profiles previously reported *in vitro*.

### TH211 enables identification of functional tyrosine sites on kinases

**Fig. 4:**
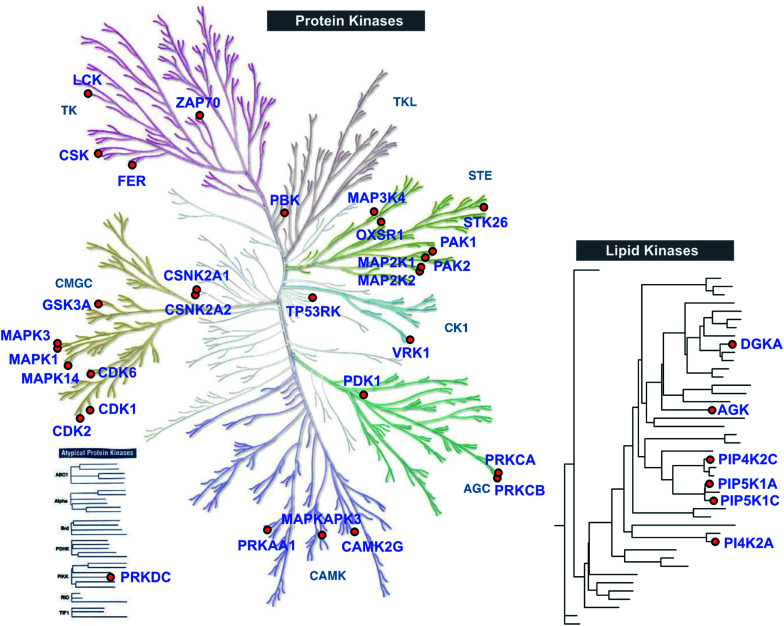
TH211 enables live cell chemoproteomic profiling of human protein and lipid kinases. Kinome tree of proteins that show specific enrichment from SILAC Jurkat cells treated with TH211 probe (50 μM) or DMSO vehicle, respectively, for 2 h at 37 °C followed by quantitative LC-MS chemical proteomics. See ESI† for selection criteria of kinases shown in the protein and lipid kinome trees. See Table S1† for complete list of TH211-modified sites on kinases from live Jurkat cell studies. The protein kinase tree was generated using KinMap as previously described.^63^ Kinome tree illustration reproduced courtesy of Cell Signaling Technology, Inc. (http://www.cellsignal.com). The lipid kinase tree was generated in-house as previously described.^34^ All data shown are representative of 3 experiments (*n* = 3 biologically independent experiments).

**Fig. 5:**
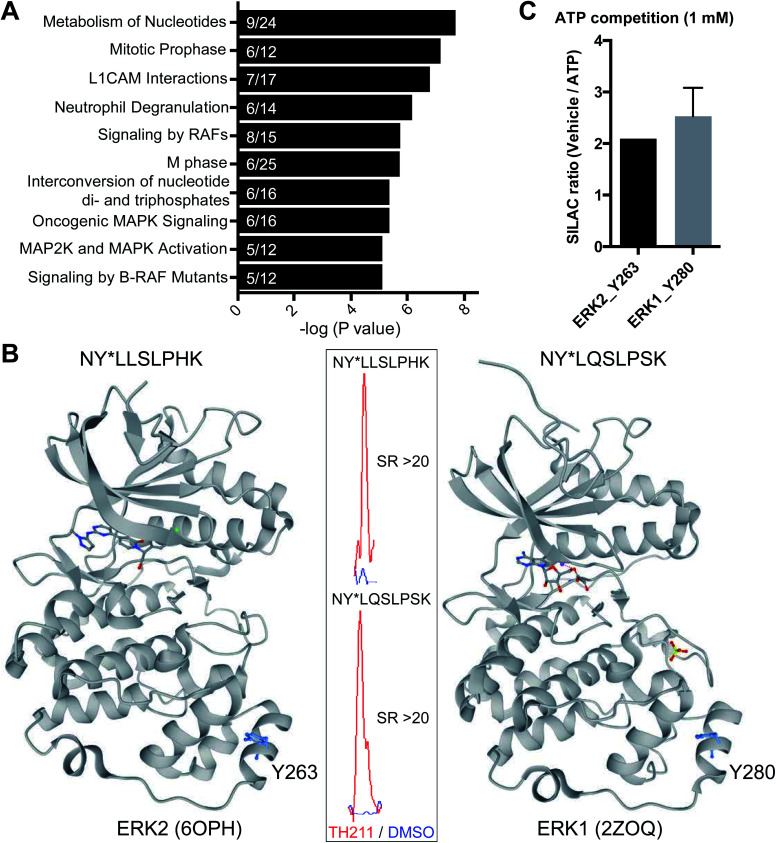
TH211 targets substrate binding sites of kinases mediating MAPK signaling. (A) Reactome pathway enrichment analysis of probe-modified protein kinases identified in TH211-treated Jurkat cells (50 μM probes, 2 h, 37 °C). Kinases used for analyses showed specific enrichment based on SILAC ratios from light – TH211/heavy – vehicle comparisons. See ESI† for selection criteria of kinases. Reactome pathway enrichment analyses were performed with ReactomePA^64^ using a sample space containing only kinases. Kinases included in the sample space were determined by using reviewed human proteins classified under the kinase keyword KW-0418 in Uniprot (https://www.uniprot.org/). Numbers inside of each bar represent the frequency in sample dataset compared with frequency of the kinase sample space. (B) Crystal structure of TH211-modified sites that differentiate the substrate-binding region of ERK2 (PDB accession code: 6OPH) and the homologous site on ERK1 (PDB accession code: 2ZOQ). ERK2 and ERK1 crystal structures show binding of inhibitors in the canonical ATP-binding pocket of the respective kinase. Inset: specific enrichment of ERK probe-modified sites was confirmed by a SILAC light – TH211 (red)/heavy – DMSO vehicle (blue) MS1 peptide abundance ratio >5. (C) Sensitivity of ERK probe-modified sites to competition with free ATP in *in vitro* assays using Jurkat proteomes. Light and heavy Jurkat proteomes were treated with PBS vehicle or free ATP (1 mM), respectively, for 1 h followed by TH211 probe labeling (100 μM, 1 h, 37 °C). The degree of inhibition at Y263 (ERK2) and Y280 (ERK1) was assessed by the SILAC ratio (SR) of light to heavy MS1 peptide abundances. All data shown are representative of *n* = 2–3 biologically independent experiments.

Closer inspection of our Jurkat data revealed a substantial number of kinases modified by TH211 in live cells (>50 kinases, Fig. 4; see ESI† for details of kinase analysis). The majority of probe-modified kinases mediate phosphorylation of protein substrates (Fig. 4 and Table 1). We performed a Reactome pathway enrichment analysis to gain further insights into molecular pathways that are overrepresented among kinases in our TH211-modified kinase dataset^44^ (Fig. 5A and S6†). From this global analysis, we identified enrichment in several pathways involved in signal transduction including mitogen-activated protein kinase (MAPK) signaling and activation (signaling by RAFs, MAPK signaling/activation; Fig. 5A). A common mediator of these signaling pathways are the extracellular signal-regulated kinases (ERK1 and ERK2) that regulate proliferation, differentiation, apoptosis, and migration as part of the MAPK pathway.^45–47^ In contrast to our previous chemoproteomic studies to map ATP binding sites of ERK isoforms,^48^ we identified TH211-modified tyrosines in the F-site recruitment site (FRS) of ERK2 (Y263) involved in binding substrates that contain a conserved Phe-X-Phe-Pro consensus sequence docking site^47,49,50^ (DEF motif, Fig. 5B). We also identified a homologous TH211-modified site on ERK1 (Y280). We performed competition studies *in vitro* with free ATP (1 mM) to show moderate competition (∼50%, SR ∼2), which supports functional binding from TH211 at these ERK sites (Fig. 5C; see ESI Methods† for details of ATP competition assay). Given that the FRS is only accessible in the active phosphorylated ERK1/2,^47^ our findings identify ligandable tyrosines for blocking FRS protein–protein interactions in future medicinal chemistry efforts. The full list of TH211-modified binding sites and corresponding kinases can be found in Table S1.†

**Table 1:** Probe-modified kinases and binding sites from respective domains detected in TH211-treated Jurkat cells

Uniprot	Gene name	Modified sites	Domain name
O95747	OXSR1	229|227|	Protein kinase domain
P05771	PRKCB	518|515|	Protein kinase domain
P05771	PRKCB	209|	C2 domain
P06239	LCK	192|209|	SH2 domain
P06239	LCK	470|394|441|	Protein kinase domain
P06493	CDK1	286|181|270|	Protein kinase domain
P16591	FER	714|	Protein kinase domain
P17252	PRKCA	515|512|504|	Protein kinase domain
P17252	PRKCA	195|232|209|	C2 domain
P19367	HK1	346|	Hexokinase domain
P19784	CSNK2A2	308|	Protein kinase domain
P23743	DGKA	240|	Zinc finger phorbol-ester/DAG-type
P24941	CDK2	236|237|	Protein kinase domain
P27361	MAPK3	280|	Protein kinase domain
P28482	MAPK1	263|	Protein kinase domain
P36507	MAP2K2	233|187|101|	Protein kinase domain
P41240	CSK	97|	SH2 domain
P41240	CSK	67|64|	Src homology 3 (SH3) domain
P43403	ZAP70	506|372|	Protein kinase domain
P43403	ZAP70	164|	SH2 domain
P49840	GSK3A	355|	Protein kinase domain
P52789	HK2	398|401|	Hexokinase domain
P68400	CSNK2A1	307|182|	Protein kinase domain
P78527	PRKDC	2965|3168|2936|3475|	FAT domain
Q00534	CDK6	257|43|	Protein kinase domain
Q02750	MAP2K1	97|	Protein kinase domain
Q13131	PRKAA1	190|247|	Protein kinase domain
Q13153	PAK1	429|	Protein kinase domain
Q13177	PAK2	408|	Protein kinase domain
Q13555	CAMK2G	231|	Protein kinase domain
Q15118	PDK1	365|	Histidine kinase core domain
Q16539	MAPK14	249|182|103|	Protein kinase domain
Q16644	MAPKAPK3	263|76|	Protein kinase domain
Q16774	GUK1	53|	Guanylate kinase-like domain
Q8NEV1	CSNK2A3	307|182|	Protein kinase domain
Q8TBX8	PIP4K2C	384|	Phosphatidylinositol phosphate kinase (PIPK) domain
Q96KB5	PBK	100|74|	Protein kinase domain
Q96S44	TP53RK	60|67|	Protein kinase domain
Q99986	VRK1	194|213|98|113|107|311|188|317|187|147|214|302|314|	Protein kinase domain

Although the majority of TH211 kinase targets were assigned to the protein kinase class, we observed a substantial fraction of modified kinases involved in phosphorylation of metabolites (∼40% of all detected kinases; Fig. 4 and Table S1†). In addition to DGKα, we identified several TH211-modified lipid kinases involved in phosphorylation of phosphatidylinositol (PI)-phosphate analogs to produce the secondary messenger PIP2 (ref. 51) (PIP5K1C, PIP5K1A, PIP4K2C; Fig. 4). We also identified a TH211-modified binding site (Y465) in the C-lobe of the catalytic domain of PI4K2A that is important for membrane association^52^ (Fig. 4 and Table S1†). In addition to phospholipid metabolism, we identified TH211-modified sites near the DAGKc domain (Y224) of the lipid kinase AGK, which is involved in phosphorylation of glycerol lipids^53^ (Fig. 4 and Table 1). The remaining probe modified-kinases largely mediate phosphorylation in glycolysis and metabolic pathways of nucleotides and sugars (Reactome pathways enrichment analysis of non-protein kinases, Fig. S7†).

**Fig. 6:**
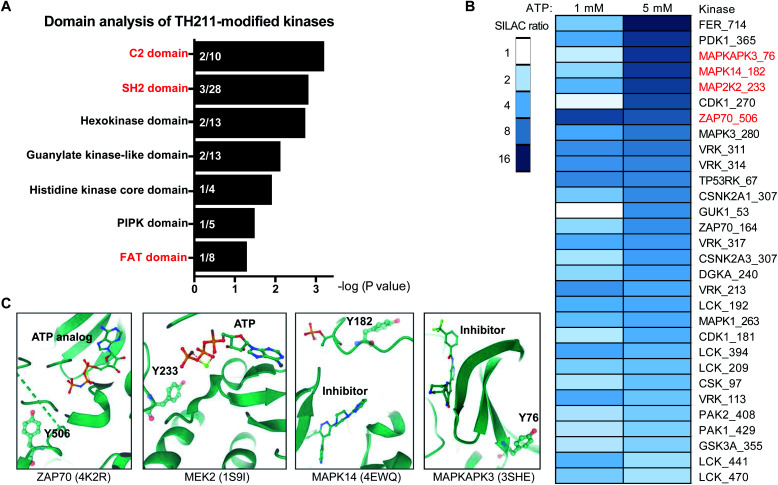
Discovery of ligandable tyrosine sites in kinase functional domains in live cells. (A) Probe-enriched domains of kinases from TH211-treated Jurkat cells (50 μM probes, 2 h, 37 °C). Probe-modified peptides used for analyses showed specific enrichment based on SILAC ratios from light – TH211/heavy – vehicle comparisons. See ESI† for selection criteria of kinase peptides. Domain annotations are plotted as a function of the enrichment value (−log *P* value after binomial test) comparing the probe-modified domains to a sample space containing only non-kinase domains on kinases. Kinases used for the sample space were determined by using reviewed human proteins from the kinase keyword KW-0418 in UniProt (https://www.uniprot.org/). (B) Heatmap shows the kinase, TH211-modified tyrosine site identified in live cells, and degree of inhibition of probe labeling with free ATP competition. The magnitude of inhibition of probe labeling was quantified using SILAC ratios of MS1 peptide abundances from light and heavy Jurkat proteomes treated with PBS vehicle or free ATP (1 or 5 mM), respectively, for 1 h followed by TH211 probe labeling (100 μM, 1 h, 37 °C). (C) Crystal structures showing proximity of free ATP-competed, probe-modified tyrosine sites on kinases to ATP substrate or inhibitor molecules. The kinase target, Protein Data Bank (PDB) identifier, TH211-modified tyrosine site, and ATP substrate or inhibitor are shown. All data shown are representative of *n* = 2–3 biologically independent experiments.

An enabling feature of our approach is to identify new ligand sites on protein kinases that may not be amenable to discovery with traditional biochemical and ATP-based chemoproteomic tools. Considering our TH211 studies are performed *in situ*, we have an opportunity to map ligand binding sites of kinases that may only be accessible and detected in live cell environments. These studies could also reveal ligand binding sites outside of the catalytic domain. We performed a domain enrichment analysis on probe-modified sites^17^ to evaluate domains within kinases that are targeted by TH211 (Fig. 6A and Table 1). Interestingly, we identified non-catalytic domains that showed preferential, albeit with varying degrees of statistical significance, enrichment with TH211. Several of these regulatory domains have discrete roles in regulating and localizing the activity of the catalytic domain of kinases.^54^

We performed ATP competition studies in Jurkat lysates to determine whether TH211 binding to kinase sites was activity dependent. We identified a suite of kinase tyrosine sites that were sensitive to blockade of probe labeling with free ATP (Fig. 6B). We tested different concentrations of ATP to evaluate concentration dependence, which can in some instances help localize the ATP binding region of kinase active sites. For example, we compared sensitivity of Y506 and Y164 on ZAP70 kinase and found that the former probe-modified site was potently competed at both ATP concentrations tested (1 and 5 mM free ATP, Fig. 6B). These findings are in agreement with crystal structures showing that Y506 is localized in the ATP-binding pocket and in direct proximity to an ATP analog (PDB ID: 4K2R, Fig. 6C). The reduced sensitivity of Y164 to ATP competition is in agreement with the location of this site in the SH2 domain of ZAP70 (Y164, Fig. 6B). T cell activation leads to TCR phosphorylation, which provides docking signals for SH2-mediated recruitment of ZAP70 and subsequent phosphorylation and activation of this kinase.^55^ The identification of a tyrosine-modified site (Y164) in vicinity of the phospho-recognition site of ZAP70 SH2 domain presents an opportunity in future studies to inactivate ZAP70 *via* blockade of its localization to the TCR.

We identified additional examples of functional TH211 binding to kinases by comparing ATP-competed sites in our chemical proteomic assay to their respective active site location in crystal structures. For example, the Y233 site of MAP2K2 (MEK2) kinase showed concentration-dependent blockade of probe labeling in lysates treated with free ATP (SR of 2 and 6 for 1 and 5 mM ATP, Fig. 6B). Co-crystal structures of MEK2 with ATP substrate^56^ verified our chemical proteomic findings by showing Y233 in close proximity to substrate in the MEK2 active site (PDB ID: 1S9I, Fig. 6C). We also identified probe-modified tyrosines in active site regions of MAPK14 and MAPKAPK3 that mediate binding to reported inhibitors. The sensitivity of Y182 and Y76 to ATP competition are in agreement with location of these tyrosine sites in ATP-binding regions of MAPK14 (ref. 57) (PDB ID: 4EWQ) and MAPKAPK3 (ref. 58) (PDB ID: 3SHE, Fig. 6C).

In summary, we demonstrate that TH211 is suitable for live cell chemoproteomic profiling of protein and metabolic kinases to reveal tyrosine (and lysine) sites in catalytic and regulatory domains of kinases. Identification of ligandable tyrosine sites in ATP and inhibitor binding regions of kinases provides future opportunities for developing covalent inhibitors against these key signaling proteins.

### Identification of C2 domain sites important for downregulation of PKC-α in response to PMA

Among candidate sites in kinase regulatory domains, we selected tyrosine-(Y195) and lysine-modified sites (K209, K232) in the C2 domain of protein kinase C-alpha for further studies (PRKCA or PKC-α, Table 1). The C2 domain is important for regulating activation of PKC-α-mediated signaling in cells.^59^ To test the function of these probe-modified sites, we performed mutagenesis studies to evaluate response of recombinant wild-type and mutant PKC-α proteins to cell activation with the DAG mimetic phorbol-12-myristate-13-acetate (PMA^60^). Specifically, prolonged activation with PMA triggers degradation of phorbol ester-responsive PKC isoforms to downregulate cellular responses.^61^

**Fig. 7:**
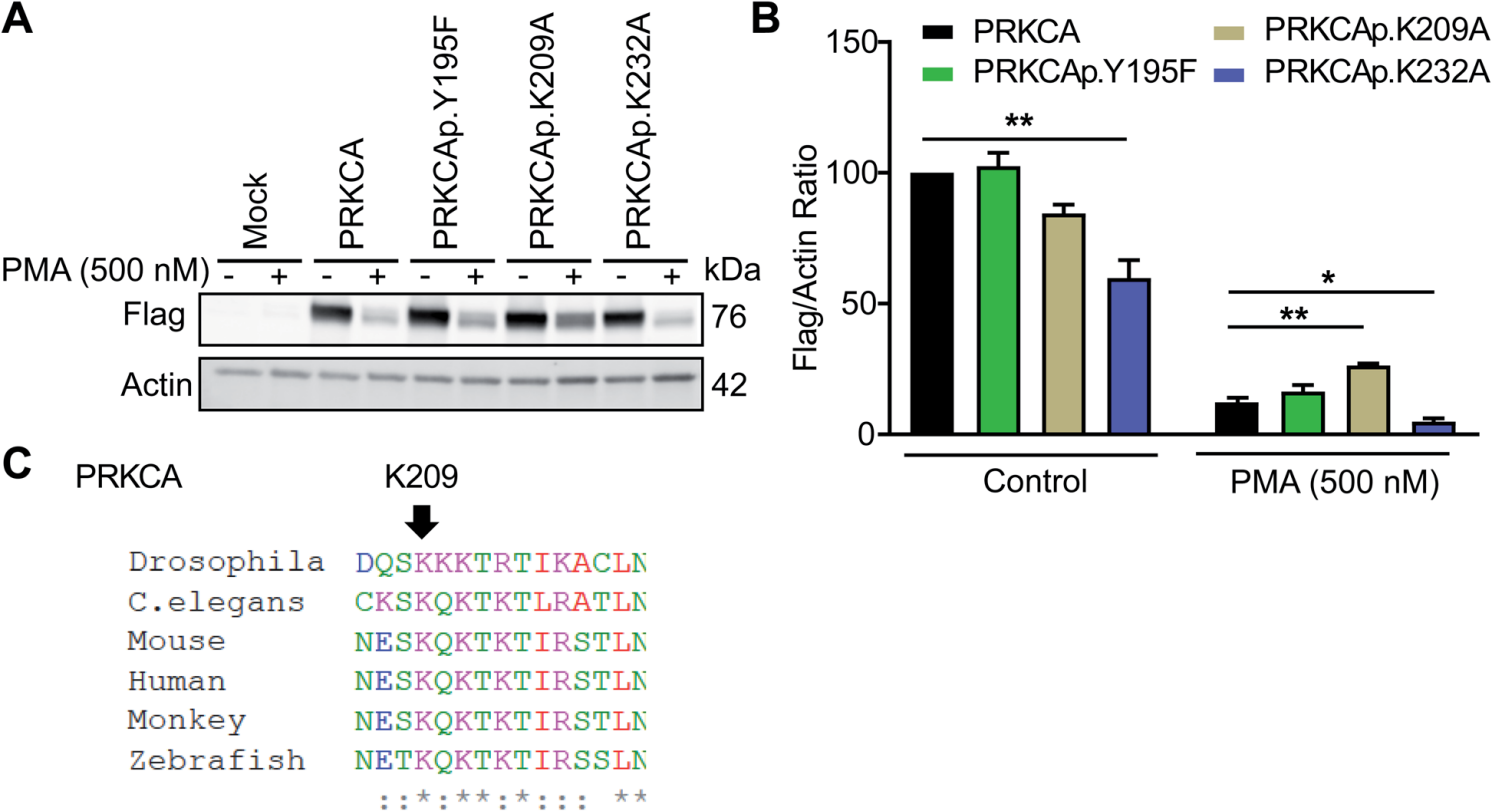
Probe-modified sites identified in the C2 domain are functionally important for PMA-mediated downregulation of PKC-α. Recombinant PKC-α (PRKCA) wild-type or mutant overexpressed HEK293T cells were stimulated with vehicle or PMA (500 nM) for 6 h followed by western blot analysis of recombinant protein levels (anti-flag; A and B). Actin signals were used as a loading control to normalize recombinant protein levels between samples. Evaluation of western blot signals showed a reduced degradation for the PRKCA K209A mutant upon prolonged PMA treatment. (C) Protein sequence alignment shows PRKCA K209 is evolutionary conserved among different species. All data shown are representative of *n* = 3 biologically independent experiments. See Fig. S8† for western blot data from the independent replicate experiments. *P < 0.05; **P < 0.01 for mutant *versus* wild-type PRKCA.

Consistent with previous reports, we observed substantial loss of PKC-α in recombinantly overexpressing HEK293T cells treated with PMA under chronic activation conditions (500 nM PMA, 6 h; Fig. 7A and S8†). Evaluation of PKC-α mutant response under the same activation paradigm showed equivalent downregulation (∼88% loss of protein) for the Y195F mutant protein. In contrast, PKC-α K209A mutant showed a significantly blunted response compared with its wild-type counterpart (∼68%, Fig. 7B). The observed differences in downregulation of PKC-α wild-type and mutant proteins were not due to expression because basal (control) recombinant protein levels were comparable with the exception of K232A, which showed significantly reduced control and PMA-stimulated protein levels (Fig. 7B). Sequence homology analyses revealed that K209 of PKC-α is evolutionarily conserved and further supports the functional importance of this probe modified amino acid (Fig. 7C and S9†).

Collectively, our studies support the ability of TH211 to identify functional sites in the C2 domain of PKC-α that are important for downregulation during chronic stimulation with PMA. Future studies will focus on determining whether liganding C2 domain sites can be utilized for pharmacological modulation of PKC-α levels and function in activated cells.

## Conclusion

Here, we demonstrate that a distinct advantage of the SuTEx electrophile is the capability for introducing structurally complex binding elements into the LG for directing molecular recognition on proteins without compromising the ability to map probe-modified sites by LC-MS chemical proteomics. The triazolide LG of SuTEx probes is well-suited as a traceless linker in LC-MS studies to expedite direct proteome-wide identification of binding sites in living systems. As proof of concept, we combined an existing lipid kinase fragment binding element with the SuTEx electrophile and bioorthogonal reporter tags to produce a kinase covalent probe (TH211) suitable for lysate and live cell chemoproteomic profiling (Fig. 4). We evaluated TH211 activity in live Jurkat T cells to identify modified sites in ATP- and lipid-substrate binding regions of DGKα, which matched previous chemical proteomics studies using orthogonal ATP-based probes (Fig. 3). Our finding that mutation of a probe-modified site in the C1 domain results in loss of catalytic activity of human DGKα helps support functional TH211 binding (Fig. S5†).

TH211 activity was evaluated across the kinome to identify additional metabolic and protein kinases that contained tyrosine and lysine sites amenable to SuTEx reaction (Fig. 4 and Table 1). Importantly, we demonstrate that TH211-binding to these kinase targets in lysates and live cells is activity dependent and can be blocked using ATP substrate and kinase inhibitors (Fig. 3 and 6). Interestingly, we identified TH211-modified sites in the C2 domain of PKC-α and demonstrated by site-directed mutagenesis that perturbations to a lysine site (K209) can affect downregulation of PKC-α upon prolonged exposure to PMA activation (Fig. 7). Additional follow-up studies are needed to determine the mechanism of PMA-induced degradation of PKC-α C2 domain mutants (*e.g.* proteasome-dependent or -independent pathways) and whether PKC-α protein function can be controlled through liganding of C2 domain sites using selective SuTEx compounds.

Another outcome from our LC-MS chemical proteomic studies was the inability to detect TH211-modified peptides that corresponded to any of the known human serotonin receptors (5-HTRs). These findings were somewhat surprising given that RF001 is derived from ritanserin, which is a potent inverse agonist of 5-HTR.^62^ Several factors could contribute to the lack of identification of peptides from 5-HTRs including low abundance, activation state, and probe-modified peptides that are not LC-MS compatible. Future studies evaluating additional cell types and LC-MS method are likely needed to conclusively determine whether RF001-based SuTEx probes engage these GPCRs.

In summary, we envision our chemoproteomic strategy can be generally extended to streamline LC-MS identification of binding sites of drug molecules and other chemically complex compounds, which is an important step towards understanding mode of action of small molecules.

## Methods

Detailed Methods are provided in the ESI†

## Conflicts of interest

There are no conflicts to declare.

## Supplementary Material

SC-012-D0SC06623K-s001

SC-012-D0SC06623K-s002

## References

[cit1] Schreiber S. L., Kotz J. D., Li M., Aube J., Austin C. P., Reed J. C., Rosen H., White E. L., Sklar L. A., Lindsley C. W., Alexander B. R., Bittker J. A., Clemons P. A., de Souza A., Foley M. A., Palmer M., Shamji A. F., Wawer M. J., McManus O., Wu M., Zou B., Yu H., Golden J. E., Schoenen F. J., Simeonov A., Jadhav A., Jackson M. R., Pinkerton A. B., Chung T. D., Griffin P. R., Cravatt B. F., Hodder P. S., Roush W. R., Roberts E., Chung D. H., Jonsson C. B., Noah J. W., Severson W. E., Ananthan S., Edwards B., Oprea T. I., Conn P. J., Hopkins C. R., Wood M. R., Stauffer S. R., Emmitte K. A., N. I. H. M. L. P. Team (2015). Cell.

[cit2] Singh J., Petter R. C., Baillie T. A., Whitty A. (2011). Nat. Rev. Drug Discovery.

[cit3] Parker C. G., Galmozzi A., Wang Y., Correia B. E., Sasaki K., Joslyn C. M., Kim A. S., Cavallaro C. L., Lawrence R. M., Johnson S. R., Narvaiza I., Saez E., Cravatt B. F. (2017). Cell.

[cit4] Wang Y., Dix M. M., Bianco G., Remsberg J. R., Lee H. Y., Kalocsay M., Gygi S. P., Forli S., Vite G., Lawrence R. M., Parker C. G., Cravatt B. F. (2019). Nat. Chem..

[cit5] Backus K. M., Correia B. E., Lum K. M., Forli S., Horning B. D., Gonzalez-Paez G. E., Chatterjee S., Lanning B. R., Teijaro J. R., Olson A. J., Wolan D. W., Cravatt B. F. (2016). Nature.

[cit6] Zhang X., Crowley V. M., Wucherpfennig T. G., Dix M. M., Cravatt B. F. (2019). Nat. Chem. Biol..

[cit7] Spradlin J. N., Hu X., Ward C. C., Brittain S. M., Jones M. D., Ou L., To M., Proudfoot A., Ornelas E., Woldegiorgis M., Olzmann J. A., Bussiere D. E., Thomas J. R., Tallarico J. A., McKenna J. M., Schirle M., Maimone T. J., Nomura D. K. (2019). Nat. Chem. Biol..

[cit8] Weerapana E., Wang C., Simon G. M., Richter F., Khare S., Dillon M. B., Bachovchin D. A., Mowen K., Baker D., Cravatt B. F. (2010). Nature.

[cit9] Bradshaw J. M., McFarland J. M., Paavilainen V. O., Bisconte A., Tam D., Phan V. T., Romanov S., Finkle D., Shu J., Patel V., Ton T., Li X., Loughhead D. G., Nunn P. A., Karr D. E., Gerritsen M. E., Funk J. O., Owens T. D., Verner E., Brameld K. A., Hill R. J., Goldstein D. M., Taunton J. (2015). Nat. Chem. Biol..

[cit10] Yoo E., Stokes B. H., de Jong H., Vanaerschot M., Kumar T., Lawrence N., Njoroge M., Garcia A., Van der Westhuyzen R., Momper J. D., Ng C. L., Fidock D. A., Bogyo M. (2018). J. Am. Chem. Soc..

[cit11] Hacker S. M., Backus K. M., Lazear M. R., Forli S., Correia B. E., Cravatt B. F. (2017). Nat. Chem..

[cit12] Zhao Q., Ouyang X., Wan X., Gajiwala K. S., Kath J. C., Jones L. H., Burlingame A. L., Taunton J. (2017). J. Am. Chem. Soc..

[cit13] Patricelli M. P., Nomanbhoy T. K., Wu J., Brown H., Zhou D., Zhang J., Jagannathan S., Aban A., Okerberg E., Herring C., Nordin B., Weissig H., Yang Q., Lee J. D., Gray N. S., Kozarich J. W. (2011). Chem. Biol..

[cit14] Shannon D. A., Banerjee R., Webster E. R., Bak D. W., Wang C., Weerapana E. (2014). J. Am. Chem. Soc..

[cit15] Bach K., Beerkens B. L. H., Zanon P. R. A., Hacker S. M. (2020). ACS Cent. Sci..

[cit16] Martin-Gago P., Fansa E. K., Winzker M., Murarka S., Janning P., Schultz-Fademrecht C., Baumann M., Wittinghofer A., Waldmann H. (2017). Cell Chem. Biol..

[cit17] Hahm H. S., Toroitich E. K., Borne A. L., Brulet J. W., Libby A. H., Yuan K., Ware T. B., McCloud R. L., Ciancone A. M., Hsu K. L. (2020). Nat. Chem. Biol..

[cit18] Chen W., Dong J., Plate L., Mortenson D. E., Brighty G. J., Li S., Liu Y., Galmozzi A., Lee P. S., Hulce J. J., Cravatt B. F., Saez E., Powers E. T., Wilson I. A., Sharpless K. B., Kelly J. W. (2016). J. Am. Chem. Soc..

[cit19] Crawford L. A., Weerapana E. (2016). Mol. BioSyst..

[cit20] Gu C., Shannon D. A., Colby T., Wang Z., Shabab M., Kumari S., Villamor J. G., McLaughlin C. J., Weerapana E., Kaiser M., Cravatt B. F., van der Hoorn R. A. (2013). Chem. Biol..

[cit21] Hett E. C., Xu H., Geoghegan K. F., Gopalsamy A., Kyne Jr R. E., Menard C. A., Narayanan A., Parikh M. D., Liu S., Roberts L., Robinson R. P., Tones M. A., Jones L. H. (2015). ACS Chem. Biol..

[cit22] Narayanan A., Jones L. H. (2015). Chem. Sci..

[cit23] Resnick E., Bradley A., Gan J., Douangamath A., Krojer T., Sethi R., Geurink P. P., Aimon A., Amitai G., Bellini D., Bennett J., Fairhead M., Fedorov O., Gabizon R., Gan J., Guo J., Plotnikov A., Reznik N., Ruda G. F., Diaz-Saez L., Straub V. M., Szommer T., Velupillai S., Zaidman D., Zhang Y., Coker A. R., Dowson C. G., Barr H. M., Wang C., Huber K. V. M., Brennan P. E., Ovaa H., von Delft F., London N. (2019). J. Am. Chem. Soc..

[cit24] Wang R., Islam K., Liu Y., Zheng W., Tang H., Lailler N., Blum G., Deng H., Luo M. (2013). J. Am. Chem. Soc..

[cit25] Bos J., Muir T. W. (2018). J. Am. Chem. Soc..

[cit26] Bicker K. L., Subramanian V., Chumanevich A. A., Hofseth L. J., Thompson P. R. (2012). J. Am. Chem. Soc..

[cit27] Brulet J. W., Borne A. L., Yuan K., Libby A. H., Hsu K. L. (2020). J. Am. Chem. Soc..

[cit28] Dong J., Krasnova L., Finn M. G., Sharpless K. B. (2014). Angew. Chem., Int. Ed..

[cit29] Dong J., Sharpless K. B., Kwisnek L., Oakdale J. S., Fokin V. V. (2014). Angew. Chem., Int. Ed..

[cit30] Ficarro S. B., Browne C. M., Card J. D., Alexander W. M., Zhang T., Park E., McNally R., Dhe-Paganon S., Seo H. S., Lamberto I., Eck M. J., Buhrlage S. J., Gray N. S., Marto J. A. (2016). Anal. Chem..

[cit31] Browne C. M., Jiang B., Ficarro S. B., Doctor Z. M., Johnson J. L., Card J. D., Sivakumaren S. C., Alexander W. M., Yaron T. M., Murphy C. J., Kwiatkowski N. P., Zhang T., Cantley L. C., Gray N. S., Marto J. A. (2019). J. Am. Chem. Soc..

[cit32] Barone J. A., Bierman R. H., Cornish J. W., Hsuan A., Drake N. D., Colaizzi J. L. (1986). Drug Intell. Clin. Pharm..

[cit33] Boroda S., Niccum M., Raje V., Purow B. W., Harris T. E. (2017). Biochem. Pharmacol..

[cit34] Campbell S. T., Franks C. E., Borne A. L., Shin M., Zhang L., Hsu K. L. (2018). Mol. Pharmacol..

[cit35] Franks C. E., Campbell S. T., Purow B. W., Harris T. E., Hsu K. L. (2017). Cell Chem. Biol..

[cit36] Raushel J., Fokin V. V. (2010). Org. Lett..

[cit37] Rostovtsev V. V., Green L. G., Fokin V. V., Sharpless K. B. (2002). Angew. Chem., Int. Ed..

[cit38] Charton M. (2007). Prog. Phys. Org. Chem..

[cit39] Mann M. (2006). Nat. Rev. Mol. Cell Biol..

[cit40] Bern M., Kil Y. J., Becker C. (2012). Curr. Protoc. Bioinf..

[cit41] Ware T. B., Franks C. E., Granade M. E., Zhang M., Kim K. B., Park K. S., Gahlmann A., Harris T. E., Hsu K. L. (2020). Nat. Chem. Biol..

[cit42] Jiang Y., Qian W., Hawes J. W., Walsh J. P. (2000). J. Biol. Chem..

[cit43] Patricelli M. P., Szardenings A. K., Liyanage M., Nomanbhoy T. K., Wu M., Weissig H., Aban A., Chun D., Tanner S., Kozarich J. W. (2007). Biochemistry.

[cit44] Mi H., Muruganujan A., Huang X., Ebert D., Mills C., Guo X., Thomas P. D. (2019). Nat. Protoc..

[cit45] Seger R., Krebs E. G. (1995). FASEB J..

[cit46] Derijard B., Raingeaud J., Barrett T., Wu I. H., Han J., Ulevitch R. J., Davis R. J. (1995). Science.

[cit47] Roskoski Jr R. (2012). Pharmacol. Res..

[cit48] Shin M., Franks C. E., Hsu K.-L. (2018). Chem. Sci..

[cit49] Jacobs D., Glossip D., Xing H., Muslin A. J., Kornfeld K. (1999). Genes Dev..

[cit50] Lee T., Hoofnagle A. N., Kabuyama Y., Stroud J., Min X., Goldsmith E. J., Chen L., Resing K. A., Ahn N. G. (2004). Mol. Cell.

[cit51] Czech M. P. (2000). Cell.

[cit52] Zhou Q., Li J., Yu H., Zhai Y., Gao Z., Liu Y., Pang X., Zhang L., Schulten K., Sun F., Chen C. (2014). Nat. Commun..

[cit53] Hu Z., Qu G., Yu X., Jiang H., Teng X. L., Ding L., Hu Q., Guo X., Zhou Y., Wang F., Li H. B., Chen L., Jiang J., Su B., Liu J., Zou Q. (2019). Cell Metab..

[cit54] Taylor S. S., Ilouz R., Zhang P., Kornev A. P. (2012). Nat. Rev. Mol. Cell Biol..

[cit55] Lo W. L., Shah N. H., Ahsan N., Horkova V., Stepanek O., Salomon A. R., Kuriyan J., Weiss A. (2018). Nat. Immunol..

[cit56] Ohren J. F., Chen H., Pavlovsky A., Whitehead C., Zhang E., Kuffa P., Yan C., McConnell P., Spessard C., Banotai C., Mueller W. T., Delaney A., Omer C., Sebolt-Leopold J., Dudley D. T., Leung I. K., Flamme C., Warmus J., Kaufman M., Barrett S., Tecle H., Hasemann C. A. (2004). Nat. Struct. Mol. Biol..

[cit57] Roy S. M., Minasov G., Arancio O., Chico L. W., Van Eldik L. J., Anderson W. F., Pelletier J. C., Watterson D. M. (2019). J. Med. Chem..

[cit58] Oubrie A., Kaptein A., de Zwart E., Hoogenboom N., Goorden R., van de Kar B., van Hoek M., de Kimpe V., van der Heijden R., Borsboom J., Kazemier B., de Roos J., Scheffers M., Lommerse J., Schultz-Fademrecht C., Barf T. (2012). Bioorg. Med. Chem. Lett..

[cit59] Cho W., Stahelin R. V. (2006). Biochim. Biophys. Acta.

[cit60] Marquez V. E., Blumberg P. M. (2003). Acc. Chem. Res..

[cit61] Lu Z., Liu D., Hornia A., Devonish W., Pagano M., Foster D. A. (1998). Mol. Cell. Biol..

[cit62] Peng Y., McCorvy J. D., Harpsoe K., Lansu K., Yuan S., Popov P., Qu L., Pu M., Che T., Nikolajsen L. F., Huang X. P., Wu Y., Shen L., Bjorn-Yoshimoto W. E., Ding K., Wacker D., Han G. W., Cheng J., Katritch V., Jensen A. A., Hanson M. A., Zhao S., Gloriam D. E., Roth B. L., Stevens R. C., Liu Z. J. (2018). Cell.

[cit63] Eid S., Turk S., Volkamer A., Rippmann F., Fulle S. (2017). BMC Bioinf..

[cit64] Yu G., He Q. Y. (2016). Mol. BioSyst..

