# Supplementary material for: Chemoproteomic profiling of kinases in live cells using electrophilic sulfonyl triazole probes
Source: Chem Sci. 2021 Jan 21;12(9):3295–307. doi: 10.1039/d0sc06623k (PMC8179411; doi:10.1039/d0sc06623k)

Electronic Supplementary Material (ESI) for Chemical Science.
This journal is © The Royal Society of Chemistry 2021

SUPPORTING INFORMATION

Chemoproteomic profiling of kinases in live cells using electrophilic sulfonyl triazole probes

Tao Huang[1], Seyyedmohsen Hosseinibarkooie[5], Adam L. Borne[2], Mitchell E. Granade[2], Jeffrey W. Brulet[1], Thurl E. Harris[2], Heather A. Ferris[5], and Ku-Lung Hsu*[1,2,3,4]

[1]Department of Chemistry, University of Virginia, Charlottesville, Virginia 22904, United States

[2]Department of Pharmacology, University of Virginia School of Medicine, Charlottesville, Virginia 22908, United States

[3]University of Virginia Cancer Center, University of Virginia, Charlottesville, VA 22903, USA

[4]Department of Molecular Physiology and Biological Physics, University of Virginia, Charlottesville, Virginia 22908, United States

[5]Department of Medicine, University of Virginia School of Medicine, Charlottesville, Virginia 22903, United States

*Author to whom correspondence should be addressed: K.-L.H. (kenhsu@virginia.edu)

CONTENTS:

1. Supporting Figures
2. Chemical Synthesis
3. Biological Methods
4. References
5. Appendix

   5.1 NMR Spectra

   5.2 MS/MS Spectra of probe-modified peptides

1. SUPPORTING FIGURES

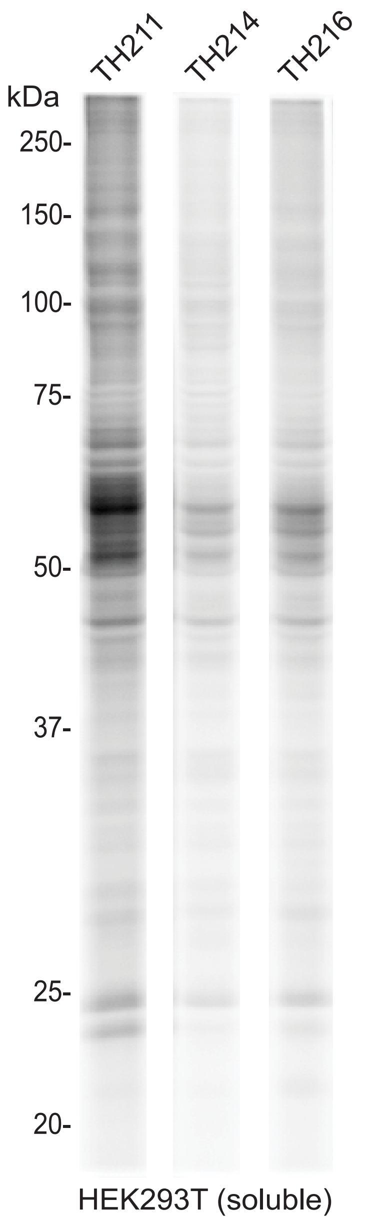

Figure S1. **AG modifications tune reactivity of RF001-SuTEx probes**. Gel-based chemical proteomic evaluation of HEK293T cell proteomes treated with RF001-SuTEx probes (100 µM, 1 hr, 37 °C) shows that AG modifications affects proteome reactivity of probes. All data shown are representative of 2 experiments (n = 2 biologically independent experiments)

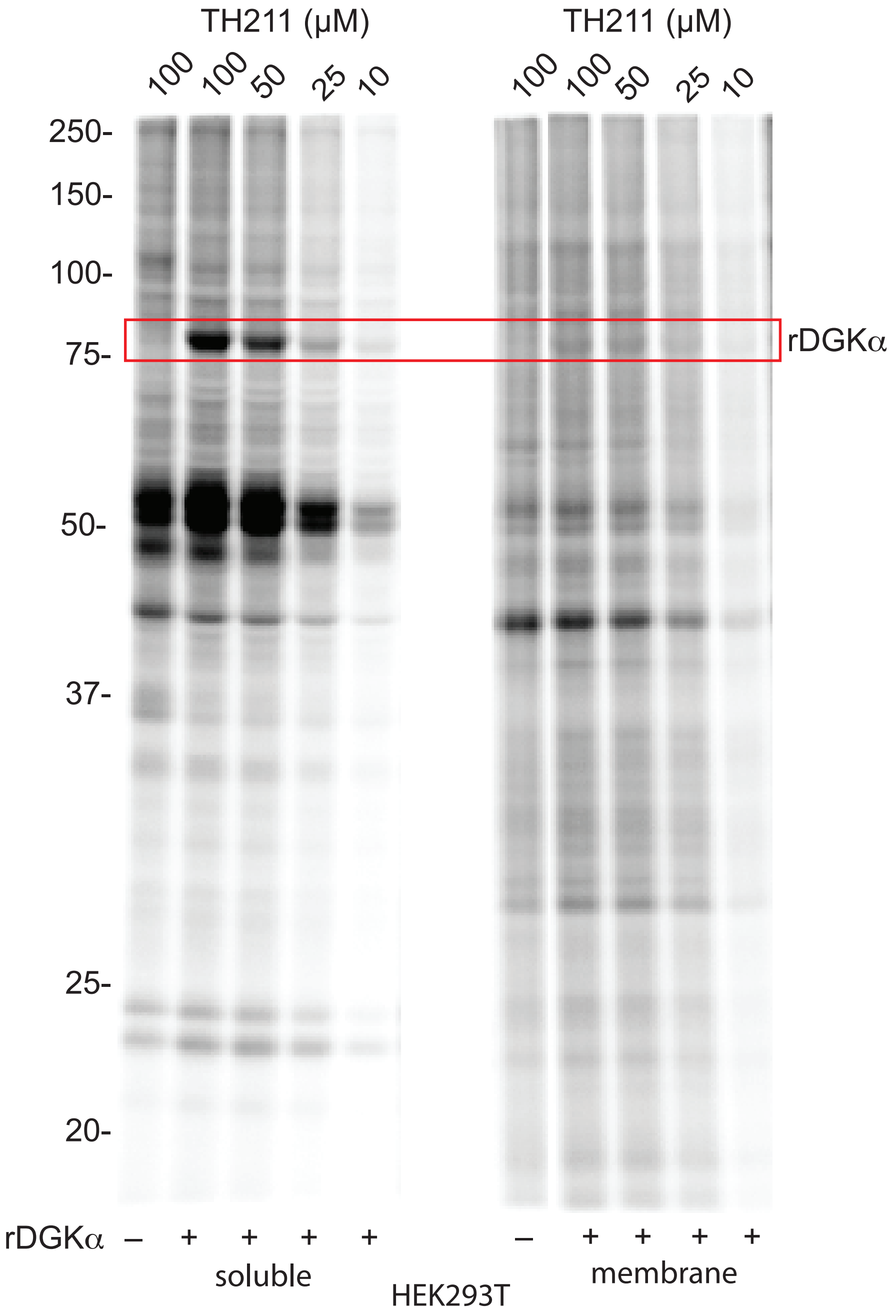

Figure S2. **RF001 binding element directs molecular recognition of SuTEx probes**. The DGKα binding element RF001 directs molecular recognition of RF001-SuTEx probes as evidenced by concentration-dependent labeling of recombinant rat DGKα (rDGKα) overexpressed in HEK293T cell proteomes (10-100 μM TH211, 1 hr, 37 °C). Mock transfected proteomes were included to aid in identification of recombinant HEK293T cell proteomes in gel-based chemical proteomic studies. All data shown are representative of 2 experiments (n = 2 biologically independent experiments)

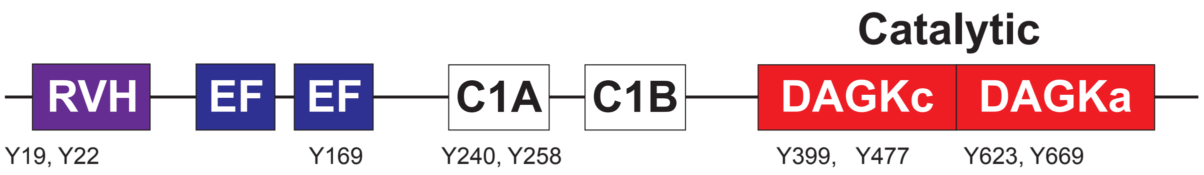

Figure S3. **Quantitative chemical proteomic evaluation of recombinant human DGKα-HEK293T soluble proteomes**. Probe-modified tyrosine sites identified and shared between TH211, TH214, and TH216 probes. Tyrosine sites shown are specifically enriched (light – probe / heavy – vehicle SILAC ratios >5) and meet our peptide quality control criteria as described in Supporting Methods. A list of all probe-modified tyrosines sites can be found in Table S1. RVH, recoverin homology domain; EF, EF Hands motif; C1, atypical/typical C1 domain.

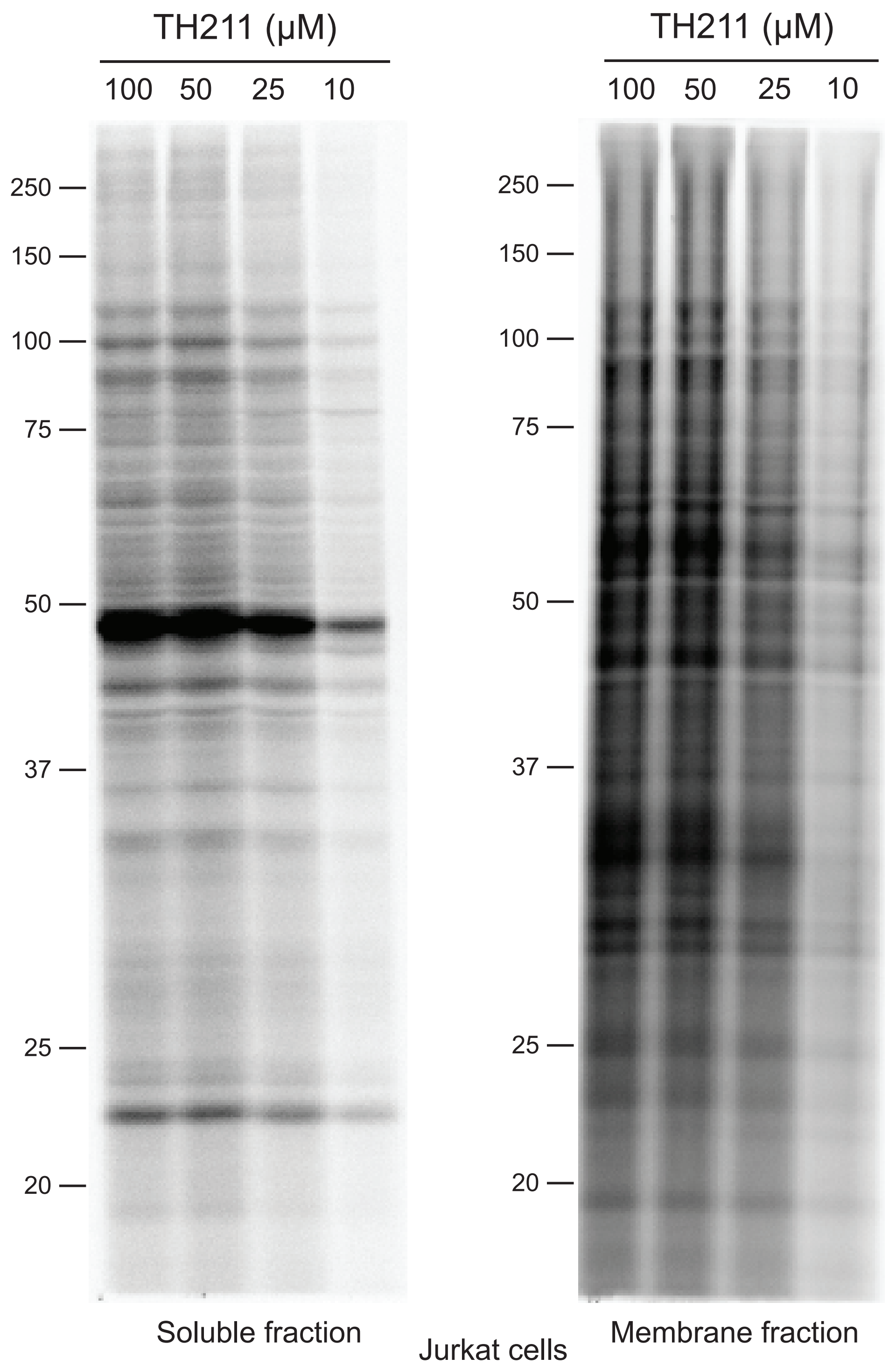

Figure S4. **TH211 is active in live cells**. Jurkat cells were treated with TH211 at varying concentrations (10-100 µM, 2 hrs, 37 °C) followed cell lysis and gel-based chemical proteomic analysis of soluble and membrane proteomes. All data shown are representative of 2 experiments (n = 2 biologically independent experiments).

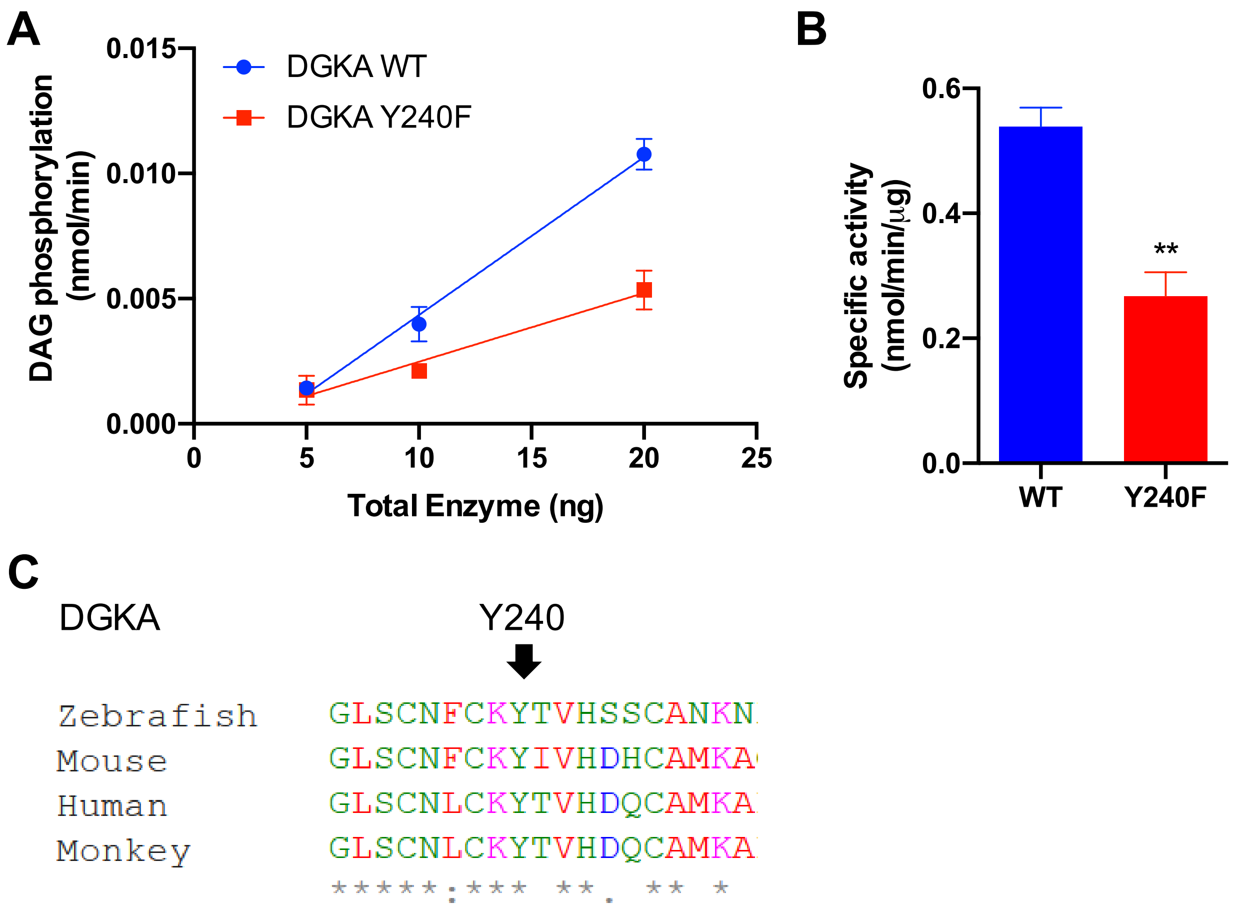

Figure S5. DGKα Y240F mutant is catalytically impaired compared with wild-type protein. (A and B) The catalytic activity of recombinant human DGKα and Y240F mutant were evaluated by a liposomal substrate assay against C18:1_C18:1 DAG substrate (n = 3 biological samples). The Y240F mutant was significantly catalytically impaired compared with wild-type protein. Data shown represent mean ± s.d. ***P* < 0.01 for Y240F versus wild-type DGKα. (C) Protein sequence alignment shows DGKA Y240 is evolutionary conserved among different species.

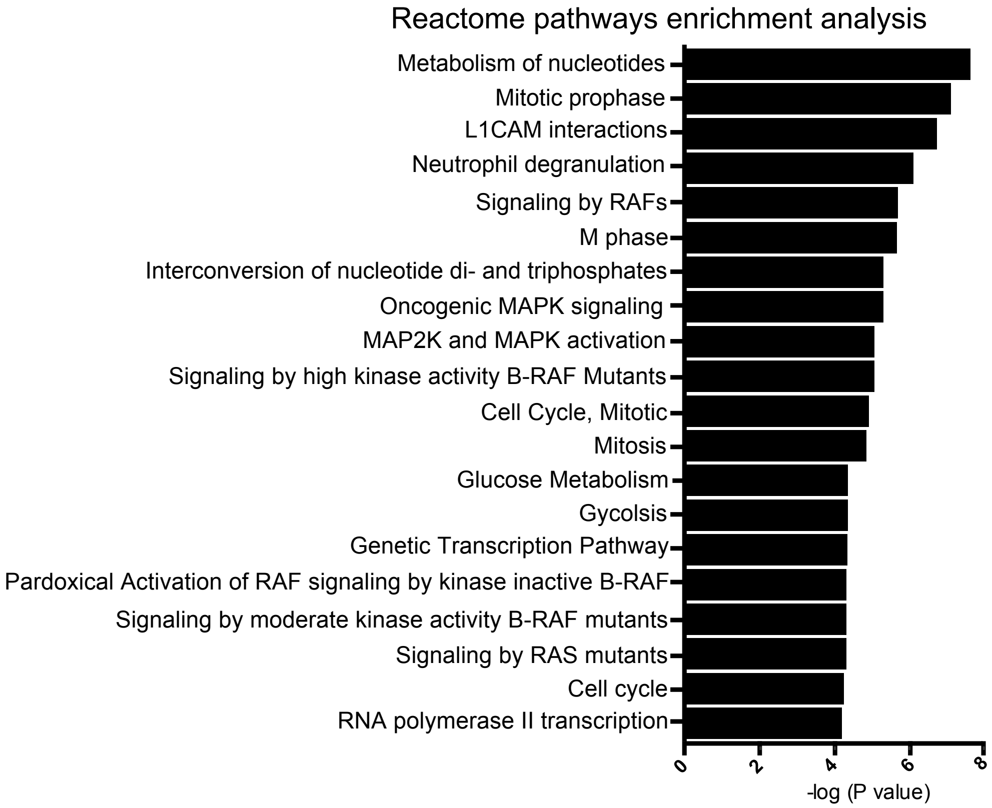

Figure S6. **TH211-modified protein kinases are enriched for signal transduction pathways**. Top 20 Reactome pathways from enrichment analysis of probe-modified protein kinases identified in TH211-treated Jurkat cells (50 μM probes, 2 hrs, 37 °C). Kinases used for analyses showed specific enrichment based on SILAC ratios from light – TH211 / heavy – vehicle comparisons. Kinases included in the sample space were determined by using reviewed human proteins classified under the kinase keyword KW-0418 in UniProt (https://www.uniprot.org/).

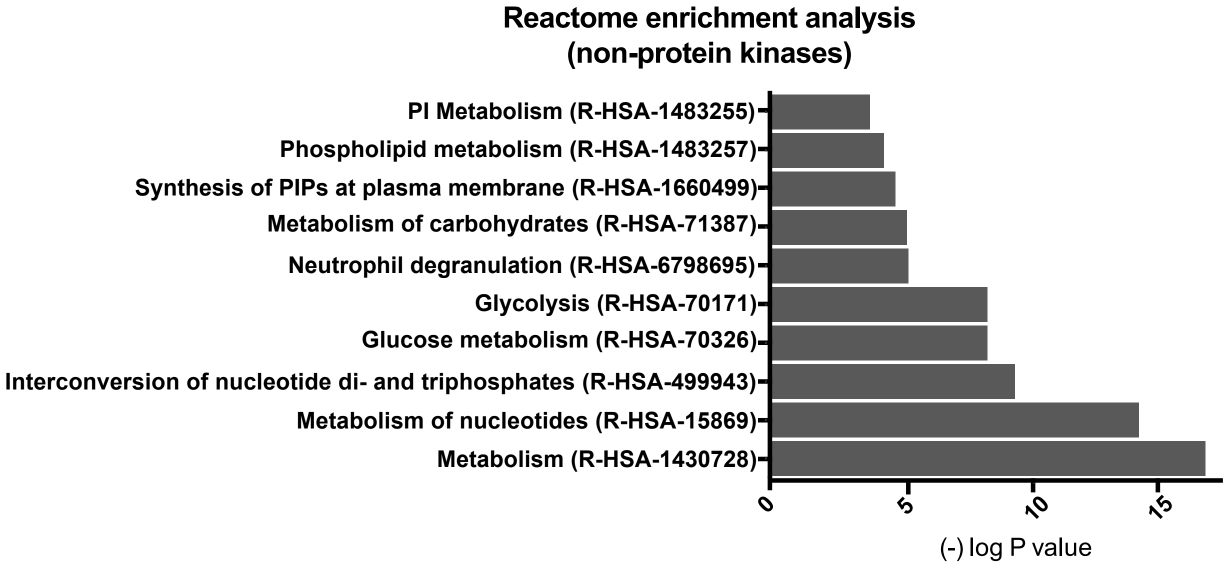

Figure S7. **Reactome pathways enrichment analysis of TH211-modified non-protein kinases**. Enrichment analyses was performed on probe-modified non-protein kinases identified in TH211-treated Jurkat cells (50 µM probes, 2 hrs, 37 °C). Kinases used for analyses showed specific enrichment based on SILAC ratios from light – TH211 / heavy – vehicle comparisons. Kinases used for the sample space were determined by using reviewed human proteins from the kinase keyword KW-0418 in UniProt (https://www.uniprot.org/).

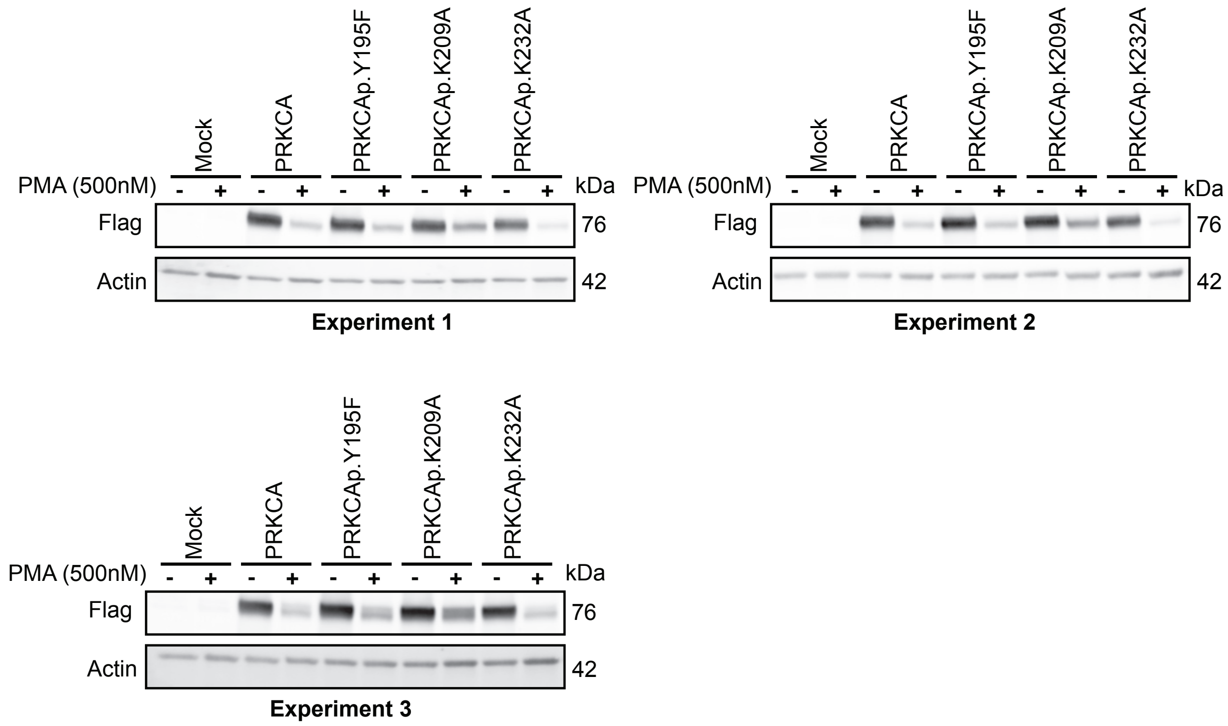

Figure S8. **Functional analysis of probe-modified sites in the C2 in PMA-mediated downregulation of PKC-α**. HEK293T cells expressing wild-type or mutant PKC-α (PRKCA) were treated with PMA (500 nM, 6 hrs) followed by western blot analysis of protein signals (anti-Flag) to determine PMA-mediated downregulation through protein degradation. Actin signals were used as a loading control for analyses.

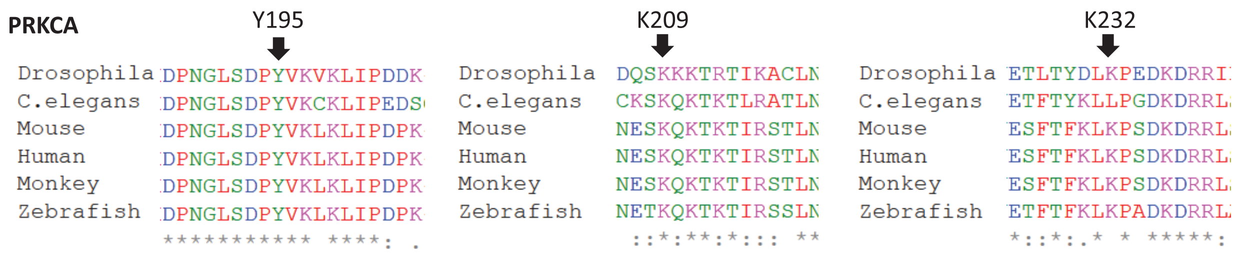

Figure S9. TH211-modified sites in PKC-α (PRKCA) C2 domain are evolutionarily conserved.

2. CHEMICAL SYNTHESIS

All chemical reagents and solvents were obtained from commercial suppliers such as Sigma Aldrich, Acros, Alfa Aesar, Combi-Blocks, and used as supplied without further purification unless stated otherwise. Analytical thin layer chromatography (TLC) was performed on Merck Silica gel 60 F254 plates (0.25 mm). Preparative TLC was conducted on Analtech Silica gel GF UV254 (1000 micron). Flash column chromatography was accomplished with Silica Gel 60 (230-400 mesh) purchased from Fisher Scientific. Detection was accomplished by using UV-light (254 nm) and/or TLC staining reagent such as phosphomolybdic acid (PMA). Nuclear magnetic resonance (NMR) spectra (^1H and ^{13}C) were recorded on a Varian spectrometer at 600 MHz at room temperature. Chemical shifts were provided in parts per million (ppm) with coupling constants in Hz. ^1H and ^{13}C spectra were calibrated in relation to deuterated solvents, namely CDCl3 (7.26 ppm for ^1H and 77.16 ppm for ^{13}C) and CD_3COCD_3 (2.05 ppm for ^1H, 29.84 and 206.26 ppm for ^{13}C). Splitting patterns for apparent multiplets were indicated as s (singlet), d (doublet), t (triplet), q (quartet), m (multiplet), br (broadened) as well as combinations of them. High resolution mass spectrometry was obtained with an Agilent 6545B LC/Q-TOF (Agilent Technologies, Santa Clara, CA, USA). Purity of all final products was greater than 97% as determined by analytical high performance liquid chromatography (HPLC) with a Shimadzu 1100 Series spectrometer detector.

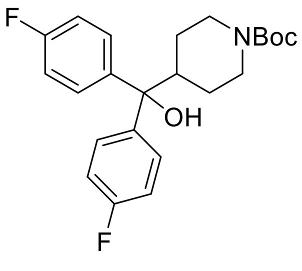

tert-Butyl 4-(bis(4-fluorophenyl)(hydroxy)methyl)piperidine-1-carboxylate (1)

The synthetic procedure was performed following previously described methods[1]. To a cooled solution of 1-Boc-isonipecotic acid ethyl ester (7.7 g, 30 mmol) in anhydrous THF (60 mL) at 0 °C was added 4-fluorophenyl magnesium bromide (2 M in diethyl ether, 36 mL, 72 mmol) over 20 min. The reaction mixture was warmed to room temperature and stirred overnight (14 hr) and then quenched with aqueous NH_4Cl solution. The aqueous layer was extracted with EtOAc 3 times and the combined organic layer was washed with brine, dried over $MgSO_4$ and concentrated on rotary evaporator. The crude material was dissolved in hot EtOAc/Hexane (1:3) and then cooled to 4 °C, and the precipitate was collected to yield compound **1** (10.5 g, 87%) as white solid. **^{1}H NMR** (600 MHz, $CDCl_3$) δ 7.46 – 7.34 (m, 4H), 6.95 (t, *J* = 8.7 Hz, 4H), 4.10 (d, *J* = 13.2 Hz, 2H), 2.66 (t, *J* = 12.8 Hz, 2H), 2.54 (broad, 1H), 2.45 (tt, *J* = 11.9, 3.1 Hz, 1H), 1.43 (m, 2H), 1.39 (s, 9H), 1.27 (qd, *J* = 12.6, 4.4 Hz, 2H). **^{13}C NMR** (150 MHz, $CDCl_3$) δ 162.30, 160.67, 154.63, 141.36, 141.34, 127.58, 127.52, 115.05, 114.91, 79.45, 79.00, 44.47, 43.93, 28.37, 26.40.

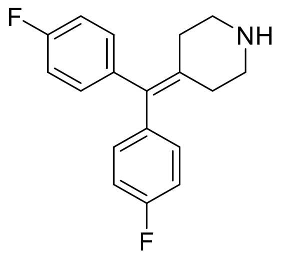

4-(Bis(4-fluorophenyl)methylene)piperidine (2, RF001)

A mixture of **1** (2.02 g, 5 mmol) and trifluoroacetic acid (10 mL) in CH_2Cl_2 (10 mL) was stirred at room temperature for overnight (14 hr) and then concentrated. The residue was diluted with NaOH (1 N) and extracted with CH_2Cl_2 several times. The combined organic layers were washed with brine, dried over MgSO4 and concentrated in vacuum to afford compound **2** (1.34 g, 94%) as a

white solid. **^{1}H NM**R (600 MHz, CD_3OD) δ 7.18 – 7.11 (m, 4H), 7.08 – 7.02 (m, 4H), 3.29 (m, 1H), 3.23 (t, *J* = 6.0 Hz, 4H), 2.56 (t, *J* = 6.0 Hz, 4H). **^{13}C NMR** (150 MHz, CD_3OD) δ 162.73, 161.10, 137.75, 137.12, 137.10, 130.86, 130.81, 129.39, 114.87, 114.73, 44.72, 27.80. **HRMS** (ESI-TOF) m/z $[M+H]^+$ calculated for $C_{18}H_{18}F_2N$ 286.1402, found 286.1404.

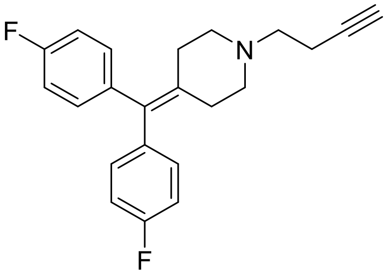

4-(Bis(4-fluorophenyl)methylene)-1-(but-3-yn-1-yl)piperidine (3)

The synthetic procedure was performed following previously described methods[2]. To a solution of **2** (1.52 g, 5.3 mmol) and 3-butynyl *p*-toluenesulfonate (1.43 g, 6.36 mmol) in DMF (15 mL) was added K_2CO_3 powder (3.66 g, 26.5 mmol). The mixture was heated to 60 °C for 6 hr and then concentrated when TLC showed most of starting material being consumed. The residue was partitioned between EtOAc and water, and the aqueous layer was extracted with EtOAc. The combined organic layers were washed with sat. Na_2CO_3, dried over $MgSO_4$ and concentrated on rotary evaporator. The product was purified by silica gel column chromatography (0 to 5% MeOH in DCM) to yield compound **3** (1.52 g, 85%) as a yellowish oil. **^{1}H NMR** (600 MHz, $CDCl_3$) δ 7.04 (dd, *J* = 8.8, 5.5 Hz, 4H), 6.95 (t, *J* = 8.8 Hz, 4H), 2.63 (dd, *J* = 8.2, 7.0 Hz, 4H), 2.53 (t, *J* = 5.6 Hz, 4H), 2.42 – 2.34 (m, 3H), 1.97 (t, *J* = 6 Hz, 1H). **^{13}C NMR** (150 MHz, $CDCl_3$) δ 162.30, 160.67, 138.07, 138.05, 135.82, 133.99, 131.28, 131.22, 115.01, 114.87, 82.70, 69.14, 56.82, 54.77, 31.47, 16.81.

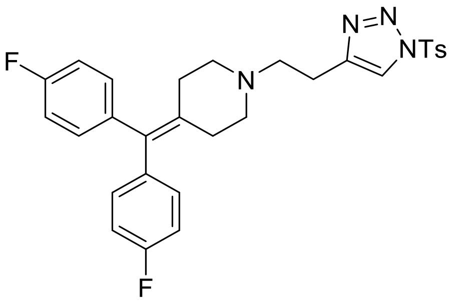

4-(Bis(4-fluorophenyl)methylene)-1-(2-(1-tosyl-1H-1,2,3-triazol-4-yl)ethyl)piperidine (TH207)

The synthetic procedure was performed following previously described methods[3]. To a solution of **3** (0.82 g, 2.43 mmol) and TsN_3 (0.58 g, 2.92 mmol) in toluene (20 mL) was added CuTC (91 mg, 20 mol%) at room temperature and the resulting mixture was stirred for 4 hr. Upon completion monitored by TLC, the reaction mixture was concentrated and loaded for column chromatography (0 to 15% acetone in DCM) to afford compound **4** (0.69 g, 50%) as a yellowish syrup, which became solid under vacuum. **^{1}H NMR** (600 MHz, $CDCl_3$) δ 8.05 – 7.87 (m, 2H), 7.36 (d, *J* = 8.3 Hz, 2H), 7.11 – 7.01 (m, 4H), 6.99 – 6.93 (m, 4H), 2.92 (t, *J* = 7.5 Hz, 2H), 2.67 (t, *J* = 7.5 Hz, 2H), 2.51 (t, *J* = 5.6 Hz, 4H), 2.43 (s, 3H), 2.36 (t, *J* = 5.6 Hz, 4H). **^{13}C NMR** (150 MHz, $CDCl_3$) δ 161.68, 160.05, 146.50, 145.55, 137.48, 135.31, 133.37, 132.62, 130.67, 130.61, 129.74, 127.95, 120.39, 114.39, 114.25, 56.23, 54.28, 30.98, 22.77, 21.17. **HRMS** (ESI-TOF) m/z $[M+H]^+$ calculated for $C_{29}H_{29}F_2N_4O_2S$ 535.1974, found 535.1984.

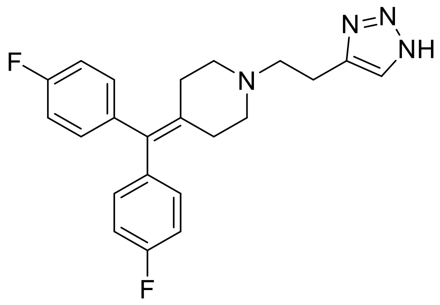

1-(2-(1H-1,2,3-Triazol-4-yl)ethyl)-4-(bis(4-fluorophenyl)methylene)piperidine (5)

To a solution of **4** (0.53 g, 1.0 mmol) in MeOH (5 mL) was added piperidine (0.43 g, 5.0 mmol) and the resulting mixture was stirred at room temperature for 0.5 hr, then concentrated and directly loaded for column chromatography (0 to 50% acetone in DCM) to afford compound **5** (0.37 g, 98%) as a yellowish syrup. **^{1}H NMR** (600 MHz, $CDCl_3$) δ 7.50 (s, 1H), 7.05 (ddd, *J* = 8.2, 5.4, 2.2 Hz, 4H), 6.97 (td, *J* = 8.7, 2.3 Hz, 4H), 2.96 (td, *J* = 7.1, 2.2 Hz, 2H), 2.75 (td, *J* = 7.1, 2.1 Hz, 2H), 2.61 (td, *J* = 5.7, 2.2 Hz, 4H), 2.48 – 2.37 (m, 4H). **^{13}C NMR** (150 MHz, $CDCl_3$) δ 162.35, 160.71, 137.93, 137.90, 135.27, 134.41, 131.24, 131.18, 130.90, 115.06, 114.92, 109.99, 56.98, 54.83, 31.40, 21.77. **HRMS** (ESI-TOF) m/z $[M+H]^+$ calculated for $C_{22}H_{23}F_2N_4$ 381.1886, found 381.1886.

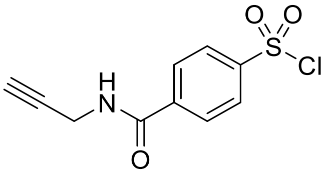

4-(Prop-2-yn-1-ylcarbamoyl)benzenesulfonyl chloride (6)

Compound **6** was synthesized as reported in our previous publication[4]. **^{1}H NMR** (600 MHz, $CDCl_3$) δ 8.13 – 7.94 (m, 4H), 7.36 (t, *J* = 5.4 Hz, 1H), 4.22 (dd, *J* = 5.4, 2.6 Hz, 2H), 2.27 (t, *J* = 2.6 Hz, 1H). **^{13}C NMR** (150 MHz, $CDCl_3$) δ 165.27, 146.32, 139.86, 128.66, 127.24, 78.85, 72.22, 30.02.

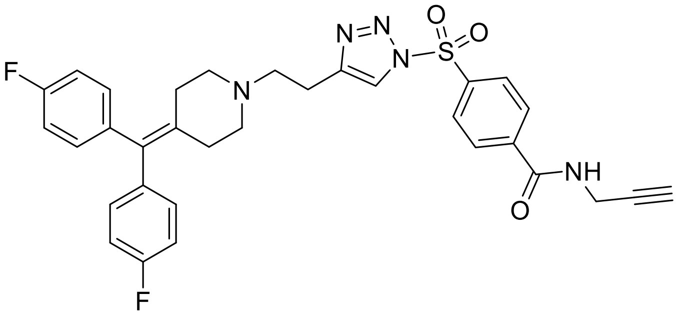

4-((4-(2-(4-(Bis(4-fluorophenyl)methylene)piperidin-1-yl)ethyl)-1H-1,2,3-triazol-1-yl)sulfonyl)-N-(prop-2-yn-1-yl)benzamide (TH211)

To a solution of compound **5** (100 mg, 0.26 mmol) and triethylamine (TEA, 75 µL, 0.52 mmol) in anhydrous DCM (3 mL) was added a solution of compound **6** (80.2 mg, 0.31 mmol) in DCM (1 mL) at 0 °C. The resulting mixture was stirred at room temperature overnight (14 hr). The reaction mixture was concentrated and then directly loaded onto preparative TLC plate for purification (10% acetone in DCM) to yield **TH211** (55 mg, 35%, after multiple purification) as white oil, which became solid under vacuum. **^{1}H NMR** (600 MHz, $CDCl_3$) δ 8.11 (d, *J* = 8.5 Hz, 2H), 8.01 (s, 1H), 7.98 (d, *J* = 8.5 Hz, 2H), 7.03 (dd, *J* = 8.5, 5.6 Hz, 4H), 6.95 (dd, *J* = 9.9, 7.5 Hz, 5H), 4.21 (dd, *J* = 5.3, 2.6 Hz, 2H), 2.93 (d, *J* = 7.4 Hz, 2H), 2.69 (t, *J* = 7.4 Hz, 2H), 2.53 (s, 4H), 2.36 (t, *J* = 5.6 Hz, 4H), 2.24 (t, *J* = 2.6 Hz, 1H). **^{13}C NMR** (150 MHz, $CDCl_3$) δ 187.96, 164.87, 162.32, 160.69, 140.22, 138.72, 137.92, 131.24, 131.18, 128.79, 128.59, 121.36, 115.05, 114.90, 78.79, 72.20, 56.60, 54.82, 31.38, 29.97, 23.17. **HRMS** (ESI-TOF) m/z $[M+H]^+$ calculated for $C_{32}H_{30}F_2N_5O_3S$ 602.2032, found 602.2032.

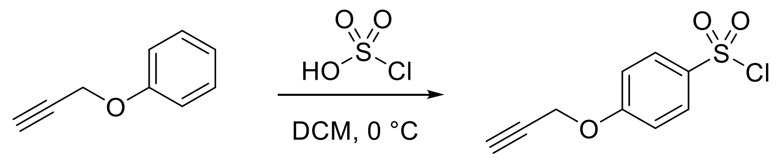

4-(Prop-2-yn-1-yloxy)benzenesulfonyl chloride (7)

The synthesis of compound **7** was adapted from a previously reported method[5]. Briefly, to a solution of phenyl propargyl ether (1.01g, 7.5 mmol) in DCM (15 mL) was added chlorosulfonic acid (3.46 g, 30 mmol) dropwise using glass syringe at 0 °C ice bath. The black mixture was stirred for 15 min, then poured into ice water to quench the reaction. The aqueous layer was extracted twice with DCM. The combined organic layers were washed with brine, dried over $MgSO_4$, and concentrated to get a greenish oil, which was purified by column chromatography (20% EtOAc in hexane) to afford compound **7** (1.08 g, 62%) as a yellowish oil. **^{1}H NMR** (600 MHz, $CDCl_3$) δ 7.98 (d, J = 9.1 Hz, 2H), 7.13 (d, J = 9.1 Hz, 2H), 4.80 (d, J = 2.4 Hz, 2H), 2.60 (t, J = 2.4 Hz 1H). **^{13}C NMR** (150 MHz, $CDCl_3$) δ 162.62, 136.86, 129.46, 115.62, 77.04, 76.86, 56.30.

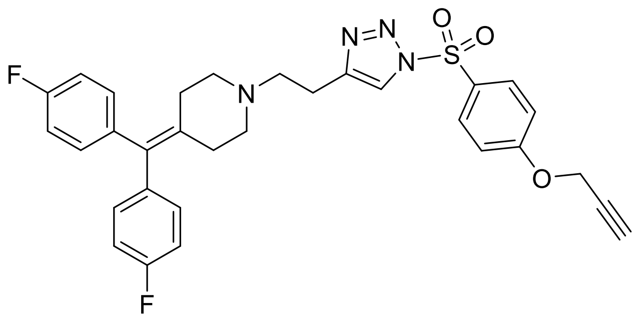

4-(Bis(4-fluorophenyl)methylene)-1-(2-(1-((4-(prop-2-yn-1-yloxy)phenyl)sulfonyl)-1H-1,2,3-triazol-4-yl)ethyl)piperidine (TH214)

TH214 was synthesized from compound **5** and **7** using the same method as **TH211**. **^{1}H NMR** (600 MHz, $CDCl_3$) δ 8.07 – 8.00 (m, 2H), 7.97 (s, 1H), 7.12 – 7.07 (m, 2H), 7.06 – 7.00 (m, 4H), 7.00 – 6.91 (m, 4H), 4.75 (d, *J* = 2.4 Hz, 2H), 2.98 (d, *J* = 7.5 Hz, 2H), 2.76 (s, 2H), 2.62 – 2.57 (m, 4H), 2.56 (m,1H), 2.42 (t, *J* = 5.5 Hz, 4H). **^{13}C NMR** (150 MHz, $CDCl_3$) δ 162.96, 162.29, 160.66, 146.09, 138.08, 138.06, 135.93, 133.99, 131.27, 131.22, 131.02, 128.16, 120.88, 115.83, 115.01, 114.87, 77.03, 76.77, 56.86, 56.21, 54.90, 31.61, 23.39. **HRMS** (ESI-TOF) m/z $[M+H]^+$ calculated for $C_{31}H_{29}F_2N_4O_3S$ 545.1818, found 545.1812.

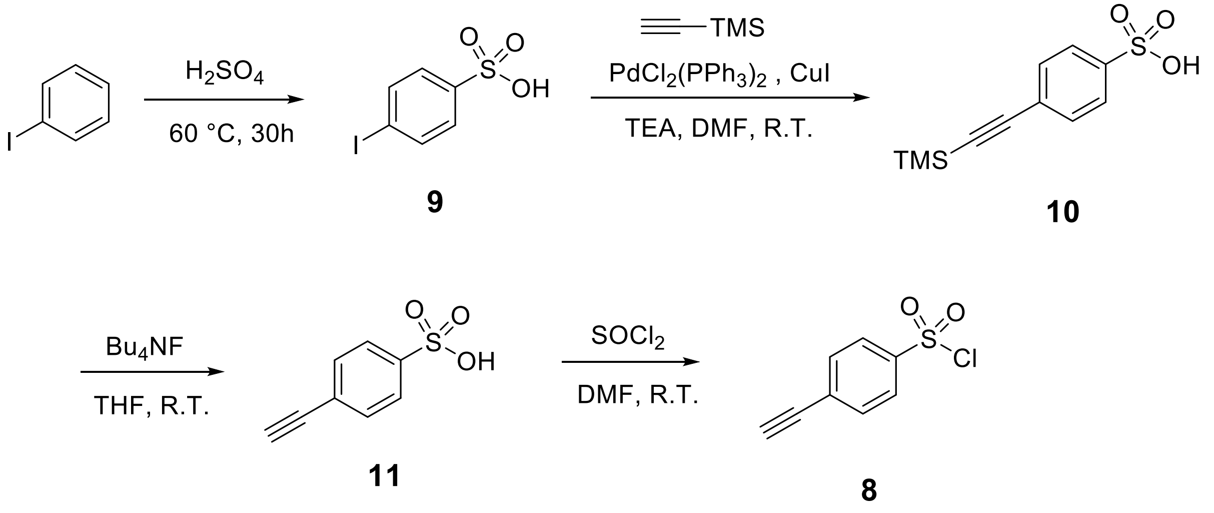

4-Iodobenzensulfonic Acid (9)

The synthetic procedure was performed following previously described methods[6]. To iodobenzene (2 mL, 3.64 g, 17.8 mmol) was added concentrated sulfuric acid (5 mL, 8.0 g, 82 mmol) and the reaction mixture was heated to 50 °C for 30 hr. Then the reaction mixture was stirred with hexane (5 mL) for 5 min in order to remove unreacted iodobenzene in the hexane layer. The sulfuric acid layer was extracted with small portions of boiling chloroform (total 20 mL) by removing the upper layer (solution of product in $CHCl_3$) with a pipette. The combined chloroform solution was concentrated to a small volume, and precipitates of the product were collected, washed with hexane, and dried to afford 4-iodobenzensulfonic acid (4.0 g, 80%), which was used for the next step without further purification.

4-((Trimethylsilyl)ethynyl)benzenesulfonic acid (10)

The synthetic procedure was performed following previously described methods[7]. To a solution of 4-iodobenzensulfonic acid (2.22 g, 7.8 mmol) in anhydrous DMF (20 mL) was added trimethylsilylacetylene (6.6 mL, 47 mmol), triethylamine (10 mL, 78 mmol), $PdCl_2(PPh_3)_2$ (550 mg, 0.78 mmol) and CuI (75 mg, 0.78 mmol), and the mixture was stirred at room temperature overnight (14 hr). After being concentrated under rotary evaporator, the residue was purified on flash silica gel column chromatography (10% MeOH in DCM) to afford the compound **10** (as form of triethylammonium salt, 2.07 g, 74%). **^{1}H NMR** (600 MHz, $CDCl_3$) δ 7.79 – 7.63 (m, 2H), 7.38 (d, J = 8.2 Hz, 2H), 3.01 (ddd, J = 7.4, 4.8, 1.0 Hz, 6H), 1.35 – 1.04 (m, 9H), 0.16 (d, J = 0.8 Hz, 9H). **^{13}C NMR** (150 MHz, $CDCl_3$) δ 145.21, 131.66, 125.74, 124.57, 104.21, 95.66, 46.35, 8.65, -0.17.

4-Ethynylbenzenesulfonic acid (11)

To a solution of compound **10** (568 mg, 1.60 mmol) in THF was added tetrabutylammonium fluoride (1.92 mL, 1 M solution in THF), and the mixture was stirred at room temperature for 2 hr. After being concentrated under rotary evaporator, the crude product was used for the next step without further purification.

4-Ethynylbenzenesulfonyl chloride (8)

To a solution of crude **11** (1.6 mmol) in DMF (3 mL) was added thionyl chloride (37 µL, 8 mmol) at 0 °C. The mixture was stirred at room temperature for 3 hr and then poured into ice water to quench unreacted thionyl chloride. The mixture was extracted with EtOAc for several times and the combined organic layers were washed with brine, dried over $MgSO_4$ and concentrated under rotary evaporator. The residue was purified on flash silica gel column chromatography to afford compound **8** (96 mg, 30% over two steps) as yellowish solid, which was immediately frozen to suppress the hydrolysis of sulfonyl chloride. **^{1}H NMR** (600 MHz, $CDCl_3$) δ 7.98 (dd, *J* = 8.6, 1.7 Hz, 2H), 7.68 (dd, *J* = 8.6, 1.7 Hz, 2H), 3.37 (s, 1H). **^{13}C NMR** (150 MHz, $CDCl_3$) δ 143.71, 133.13, 129.58, 126.95, 82.81, 81.31.

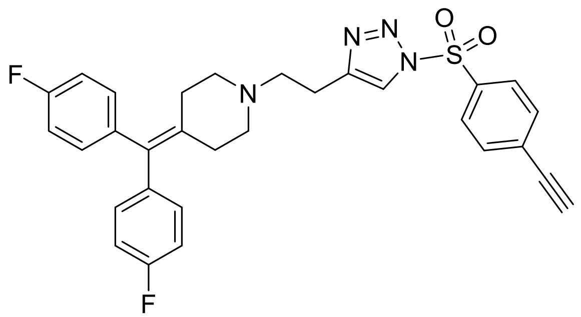

4-(Bis(4-fluorophenyl)methylene)-1-(2-(1-((4-ethynylphenyl)sulfonyl)-1H-1,2,3-triazol-4-yl)ethyl)piperidine (TH216)

TH216 was synthesized from compound **5** and **8** using the same method as **TH211**. **^{1}H NMR** (600 MHz, $CDCl_3$) δ 8.04 (d, *J* = 8.6 Hz, 2H), 7.98 (s, 1H), 7.64 (d, *J* = 8.5 Hz, 2H), 7.04 (dd, *J* = 8.6, 5.6 Hz, 4H), 6.96 (t, *J* = 8.7 Hz, 4H), 3.34 (s, 1H), 3.00 (s, 2H), 2.76 (s, 2H), 2.60 (s, 4H), 2.42 (s, 4H). **^{13}C NMR** (150 MHz, $CDCl_3$) δ 162.35, 160.72, 137.89, 137.87, 135.83, 133.16, 131.22, 131.17, 129.88, 128.46, 121.20, 115.07, 114.93, 82.79, 81.25, 56.64, 54.81, 31.24, 23.10. **HRMS** (ESI-TOF) m/z $[M+H]^+$ calculated for $C_{30}H_{27}F_2N_4O_2S$ 575.1923, found 575.1924.

3. BIOLOGICAL METHODS

Reagents

Ritanserin (purity >95%), Staurosporine (purity >98%) and ATP (disodium salt hydrate, purity >98%) used in competition studies were purchased from Cayman Chemical Company, Fisher Scientific and Acros, respectively.

Cell culture

Jurkat cells were cultured in RPMI media 1640 (Gibco) and HEK293T cells were in DMEM, both supplemented with 10% fetal bovine serum (FBS, U.S. Source, Omega Scientific) and 1% L-glutamine (Thermo Fisher Scientific). SILAC cells were cultured with corresponding SILAC media supplemented with 10% dialyzed FBS and either ^{12}C, ^{14}N-lysine and arginine (100 μg/mL) for "light" or ^{13}C, ^{15}N-lysine and arginine (100 μg/mL) for "heavy". All cells were maintained in a humidified incubator at 37 °C with 5% CO_2, and used for experiments around 90% confluency (HEK293T) or 1×10^6 cells/mL (Jurkat).

Transient transfection

HEK293T cells for overexpression studies were cultured at 37 °C with 5% CO2 in DMEM media (HyClone) with 10% fetal bovine serum (Omega Scientific), 1% L-glutamine (Fisher Scientific). The recombinant proteins were generated as previously described via transient transfection[8]. The wild-type human PKC-α plasmid was purchased from GenScript: pcDNA3.1-PRKCA-FLAG

(NM_002737.2). The wild-type human DGKα plasmid was generated in house: pGC-FLAG-DGKA.

Phorbol-12-myristate-13-acetate (PMA) activation of cells

PMA (EMD Millipore) was dissolved in DMSO to form a 500 μM solution, which was then diluted in serum free DMEM media (1:1,000, 500 nM final).48 hours after transfection, the cell culture media was removed. The cells were starved for 30 min at 37 °C with 5% CO_2 in serum free medium. The PMA was added to the final concentration of 500nm and cells were treated with PMA for 6 hours. After the PMA treatment, cells were washed and lysed in NP40 lysis buffer (50 mM Tris-HCl pH: 7.5, 1% NP40, 150 mM NaCl plus protease and phosphatase inhibitor). The cell lysates were sonicated and centrifuged for 20 minutes at 13000 rpm. Protein concentrations were determined using Bio-Rad DC kit. The degradation rate of both wild type and mutant PRKCA proteins were analyzed using western blot[4].

Western blot analysis

The supernatant after centrifugation was collected as the soluble fraction. The pelleted residue was resuspended in lysis buffer supplemented with 1% Triton X-100 followed by centrifugation at 17,000 × g for 10 minutes. The supernatant was collected as the membrane fraction. Protein concentrations were determined using Bio-Rad DC protein assay and adjusted to 1 mg/mL in PBS followed by Western blot analysis as previously described[4].

Site-directed mutagenesis

Mutated site	Primers	Sequence
h*DGK*p.Y240F	h*DGKA*p.Y240F Fr	actggtcgtgaacagtgaacttacagaggttacag
	h*DGKA*p.Y240F Re	ctgtaacctctgtaagttcactgttcacgaccagt
h*PRKCA*p.K209A	h*PRKCA*p.K209A Fr	cggatggttttggtttttttgcgcgctttcattcttgggatcagg
	h*PRKCA*p.K209A Re	cctgatcccaagaatgaaagcgcgcaaaaaaccaaaaccatccg
h*PRKCA*pY195F	h*PRKCA*pY195F-Fr	caaacgggctttcagatccttttgtgaagctgaaacttat
	h*PRKCA*pY195F-Re	ataagtttcagcttcacaaaaggatctgaaagcccgtttg
h*PRKCA*pK232A	h*PRKCA*pK232A-Fr	gtcggtctttgtctgaaggtgccaatttgaatgtaaaggactcattcc
	h*PRKCA*pK232A-Re	ggaatgagtcctttacattcaaattggcaccttcagacaaagaccgac

To introduce the mutations in *DGKA* and *PRKCA*, pcDNA3.1 vectors containing the cDNA of human *DGKA* or human *PRKCA* with a C-terminal flag signal were purchased from GenScript (www.genscript.com). To introduce three mutations in *PRKCA* and one mutation in *DGKA*, several primers were designed (see table above). Different mutations were introduced using site directed mutagenesis method (Quickexchange kit, Agilent Technologies). The sequence of each clone was confirmed using sanger sequencing.

Live cell treatment with probes and cell lysate preparation

Cells were treated with DMSO or probes (2.5 mM in DMSO, 50X, final concentration at 50 μM) in serum-free media for 2 hr at 37 °C with 5% CO_2, and then collected, washed with cold PBS for three times. For competition studies, cells were pretreated with vehicle (DMSO) or compound (50X stock in DMSO) at the same condition for 1 hr prior to probe treatment. The cell pellets were lysed by sonication (1 sec pulse, 20% amplitude, 3 times) in PBS in the presence of EDTA-free protease inhibitor cocktail tablet (Pierce). The cell lysates were subject to ultracentrifugation (100,000 x *g*, 45 min at 4 °C) to separate the cytosolic fraction in the supernatant and the membrane fraction as a pellet. The membrane pellet was re-suspended in the protease inhibitor-containing PBS by sonication. Protein concentrations in both fractions were measured by the Bio-Rad DC protein assay and adjusted to levels as needed.

Gel-based chemical proteomic assay

Proteome aliquots (2 mg/mL, 49 μL) were treated with probes at indicated concentration (1 μL, 50X stock in DMSO) at 37 °C for 1 hr. The subsequent conjugation with fluorophore was accomplished by copper-catalyzed azide-alkyne cycloaddition (CuAAC) with rhodamine-azide (TAMRA-azide, 1.25 mM, 1 μL, final concentration of 25 μM) in the presence of tris(2-carboxyethyl)phosphine (TCEP, 50 mM fresh in water, 1 μL, final concentration of 1 mM), tris[(1-benzyl-1H-1,2,3-triazol-4-yl)methyl]amine (TBTA, 1.7 mM in 4:1 *t*-butanol/DMSO, 3 μL, final concentration of 100 μM) and $CuSO_4$ (50 mM, 1 μL, final concentration of 1 mM). For samples of live cell treatment, it started with click reaction without the step of the probe treatment *in vitro*. After 1 hr incubation at room temperature, reactions were quenched by adding 4X SDS-PAGE loading buffer and beta-mercaptoethanol (17 μL) and samples were resolved by SDS-PAGE followed by in-gel fluorescence scanning.

SILAC sample preparation for MS-based chemical proteomic assay

The light proteome aliquots (2.3 mg/mL, 432 μL) were treated with the probe (5 μL, 100X fresh stock in DMSO) and the heavy proteome aliquots were treated with either the same probe or DMSO (no probe) at 37 °C for 1 hr. Samples were subjected to click reaction with the desthiobiotin-PEG3-azide (10 mM in DMSO, 10 μL, final concentration 200 μM) in the presence of TCEP (50 mM, 10 μL), TBTA (1.7 mM, 33 μL) and $CuSO_4$ (50 mM, 10 μL) at room temperature for 1 hr. The light and the heavy samples were mixed in the chloroform-methanol extraction step. The subsequent steps including reduction with dithiothreitol, alkylation with iodoacetamide, digestion with Trypsin/Lys-C, enrichment with avidin beads were conducted as previously described[4, 9].

Competitive chemical proteomics

For *in vitro* studies, the light and heavy proteomes were pretreated with vehicle (DMSO) or the inhibitor/ligand, respectively, at 37 °C for 1 hr prior to probe treatment for another 1 hr. For *in situ* studies, the light and heavy proteomes prepared from cells that were pretreated *in situ* with vehicle (DMSO) or the inhibitor, respectively, at 37 °C for 1 hr followed by probe treatment for 2 hr, were directly subjected to click reaction following the steps described above.

LC-MS/MS analysis

Data acquisition: The enriched peptide samples were analyzed by LC-MS/MS using an Easy-nLC 1200 (Thermo Scientific) coupled with an Orbitrap Q Exactive Plus mass spectrometer (Thermo Scientific). A trap column (Acclaim PepMap™, Thermo Scientific, 75 µm × 2 cm, 3 µm C18) and a homemade nanocapillary analytical column (20 cm, 5 µm C18 packed in 360 µm O.D. × 75 µm I.D. fused silica) with an integrated electrospray tip were employed with the following LC gradient (mobile phase A: 0.1% formic acid in H_2O; mobile phase B: 80% ACN, 0.1% formic acid in H_2O): 0 – 1.48 min 1% B, 400 nL/min; 1.48 – 2 min 1% B, to 300 nL/min; 2 – 10 min to 13% B, 300 nL/min; 10 – 110 min to 32% B, 300 nL/min; 110 – 151 min to 60% B, 300 nL/min; 151 – 152 min to 95% B, 300 nL/min; 152 – 160 min, 95% B, 300 nL/min; 160 – 161 min to 1% B, 300 nL/min; 161 – 161.1 min 1% B, to 400 nL/min; 161.1 – 180 min 1% B, 400 nL/min. A top 10 data-dependent acquisition method was used, which consisted of one full scan MS1 scan (m/z 375 – 1,500) followed by ten MS2 scans of the most abundant ions recorded in the MS1 scan.

Data analysis: Identification of peptides and target proteins from the LC-MS/MS raw data was achieved using the Byonic software package (Protein Metrics Inc.) to search against a modified human protein database (UniProt human protein database, angiotensin I and vasoactive intestinal peptide standards; 40660 proteins) with parameters previously described.[4] Identification of probe-modified peptides was accomplished with variable (common) modification of +635.27374 Da for TH211, +608.26284 for TH214, +578.25228 for TH216 on tyrosine and lysine residues. Fixed cysteine carbamidomethylation (+57.02146 Da) and variable modifications of methionine oxidation (+15.9949 Da), and the SILAC heavy amino acids (+10.0083 Da for R, +8.0142 Da for K) were included in search. The results from Byonic searches were imported into Skyline-daily to determine SILAC ratio (SR) of light/heavy peptide abundances[9]. To account for variations in

mixing and sample preparation, SRs of peptides from probe/no-probe (the light was treated with the probe and the heavy was treated with DMSO) were normalized to those from probe/probe (both the light and the heavy were treated with the probe). Unless otherwise stated, the results from Skyline-daily output were filtered in R to keep peptides of high quality as determined by the following criteria: Byonic score ≥ 300; a precursor mass error within 5 ppm; normalized SR ≥ 5, with both isotope dot-product (iDOTP) and ratio dot-product (rDOTP) ≥ 0.8. Comparison of probe-modified sites across all probes and cells was performed using the R package ggplot2 and Venn diagrams were generated with the VennDiagram R package[4]. Other bioinformatics analysis such as the domain enrichment analysis were performed as previously described[4]. For analysis of kinases, we also manually verified the identity of the following probe-modified peptides that did not meet our quality control criteria described above: Q53H12 (AGK, Y224), Q8TBX8 (PI42C, K384), Q9HA64 (KT3K, K45), Q96S44 (PRPK, Y67), and P27361 (MK03, Y280). Annotated MS/MS spectra that support identification of probe-modified peptides are included in the Appendix.

The analysis of TH211-modified peptides from human DGKα revealed that Y240 and Y623 appeared to be enriched for detection in membrane compared with soluble fractions. Detection of probe modification of the Y22 site of the RVH domain was enriched in soluble fraction analyses. Probe modification of Y544 appeared to be detected to an equivalent degree in both fractions.

Biochemical substrate assay of DGKα

The liposomal method for measuring purified DAG kinase activity was similar to previously described methods[8, 10]. Transient transfections with human DGKα wild-type or Y240F mutant were performed using PEI (Polysciences, Warrington, PA). Cells were fed with fresh media 24 hrs after transfection, and 48 hr following the transfection, the cells were harvested and homogenized with a 22-G needle using 500 µL/plate of lysis buffer (20 mM HEPES, pH 7.2, 150 mM NaCl, 0.5 mM DTT, 0.1% Brij-35, and the protease inhibitors phenylmethylsulfonyl fluoride (PMSF), leupeptin, and pepstatin). The cell homogenates were cleared by centrifugation at 16,000 x *g* for 10 min at 4°C. The supernatant was collected and incubated with 15 µL/plate of FLAG (M2) beads for 2 hr at 4 °C. The beads were then loaded on an affinity screening column (Fisher Scientific, Waltham, MA) and washed 3 times with lysis buffer, followed by 3 washes with wash buffer (20 mM HEPES, 150 mM NaCl, pH 7.2). The FLAG-DGKα was eluted with 5 successive additions of equal volume of 0.5 mg/mL of FLAG peptide. The fractions were collected and pooled, and purified enzyme was visualized on an SDS-PAGE gel stained with Coomassie-blue dye. The protein yield was quantified by comparison to bovine serum albumin (BSA) standards.

Lipids were prepared for liposome formation by dissolving dioleoyl phosphatidylcholine (DOPC), dioleoyl phosphatidylserine (DOPS), and dioleoyl DAG (DODG) in chloroform, combining, and drying under nitrogen gas to remove all solvent. The surface concentration of lipids within the liposomes was 10 mol% DAG, 20 mol% DOPS, and 70 mol% DOPC. The lipids were then hydrated to 10 mM in liposome buffer (50 mM (3-(*N*-morpholino)propanesulfonic acid) (MOPS), pH 7.5, 100 mM NaCl and 5 mM $MgCl_2$). The hydrated lipids were subjected to five freeze-thaw cycles in liquid nitrogen, followed by extrusion through a 100 nm polycarbonate filter 11 times. The measurement of DGK activity was determined by following the incorporation of the γ-Phosphate from [γ-^{32}P]ATP into DAG to form a radiolabeled PA product. Immediately prior to

running assays, [γ-^{32}P]ATP was prepared by adding 0.5 – 2 µL of [γ-^{32}P]ATP (6000 Ci/mmol) to 500 µL of 10 mM ATP. The assays were run as 100 µL reactions and contained liposome buffer, 0.1 mM $CaCl_2$, 1 mM dithiothreitol (DTT), appropriate purified enzyme as indicated in figure legends, 20 µL liposomes (2 mM lipids), and were initiated with the addition of 10 µL of the prepared 10 mM [γ-^{32}P] ATP. The reactions were allowed to proceed for 20 min at 30 °C before being terminated with the addition of 0.5 mL methanol with 0.1 N HCl, followed by 1 mL of ethyl acetate. Phase separation was achieved with the addition of 1 mL 1 M $MgCl_2$, and the organic phase was washed by thoroughly vortexing to remove soluble [γ-^{32}P] ATP. To measure the incorporation of [^{32}P] into DAG, the extract was centrifuged at 200 x G for 1 min, and 0.5 mL of the organic phase was removed and the radioactivity was measured using a scintillation counter. The activity of each purified DGK was normalized to background extracts without enzyme added. Activity was calculated as nmol of PA produced per min per µg of purified enzyme present in the assay.

4. REFERENCES

1. J. Lee, S. U. Kang, J. O. Lim, H. K. Choi, M. K. Jin, A. Toth, L. V. Pearce, R. Tran, Y. Wang, T. Szabo and P. M. Blumberg, *Bioorg Med Chem*, 2004, **12**, 371-385.
2. S. C. Turner, T. A. Esbenshade, Y. L. Bennani and A. A. Hancock, *Bioorg Med Chem Lett*, 2003, **13**, 2131-2135.
3. J. Raushel and V. V. Fokin, *Org Lett*, 2010, **12**, 4952-4955.
4. H. S. Hahm, E. K. Toroitich, A. L. Borne, J. W. Brulet, A. H. Libby, K. Yuan, T. B. Ware, R. L. McCloud, A. M. Ciancone and K. L. Hsu, *Nat Chem Biol*, 2020, **16**, 150-159.
5. M. Kheirabadi, G. S. Creech, J. X. Qiao, D. S. Nirschl, D. K. Leahy, K. M. Boy, P. H. Carter and M. D. Eastgate, *J Org Chem*, 2018, **83**, 4323-4335.
6. M. S. Yusubov, R. Y. Yusubova, V. N. Nemykin, A. V. Maskaev, M. R. Geraskina, A. Kirschning and V. V. Zhdankin, *European Journal of Organic Chemistry*, 2012, **2012**, 5935-5942.
7. S. Paoletta, D. K. Tosh, A. Finley, E. T. Gizewski, S. M. Moss, Z. G. Gao, J. A. Auchampach, D. Salvemini and K. A. Jacobson, *J Med Chem*, 2013, **56**, 5949-5963.
8. T. B. Ware, C. E. Franks, M. E. Granade, M. Zhang, K. B. Kim, K. S. Park, A. Gahlmann, T. E. Harris and K. L. Hsu, *Nat Chem Biol*, 2020, **16**, 170-178.
9. C. E. Franks, S. T. Campbell, B. W. Purow, T. E. Harris and K. L. Hsu, *Cell Chem Biol*, 2017, **24**, 870-880 e875.
10. S. Boroda, M. Niccum, V. Raje, B. W. Purow and T. E. Harris, *Biochem Pharmacol*, 2017, **123**, 29-39.

5. APPENDIX

5.1 NMR Spectra

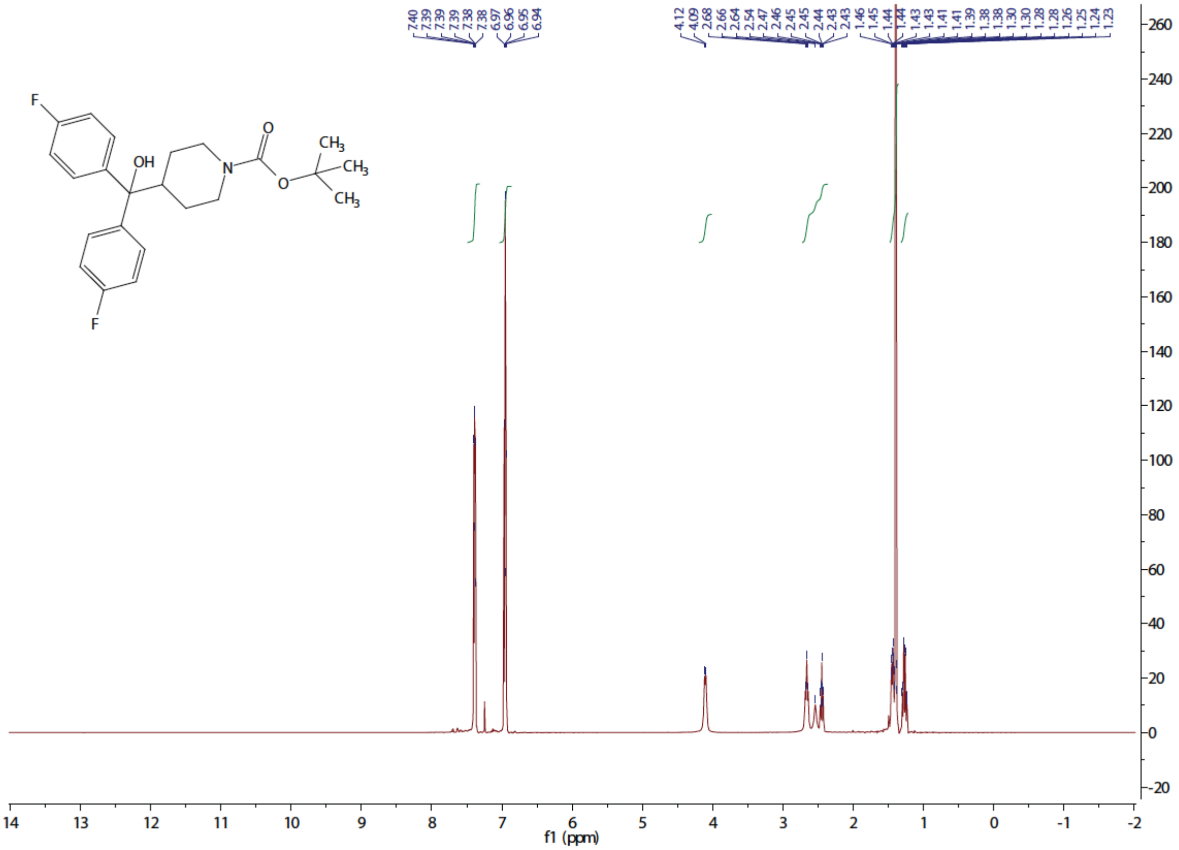

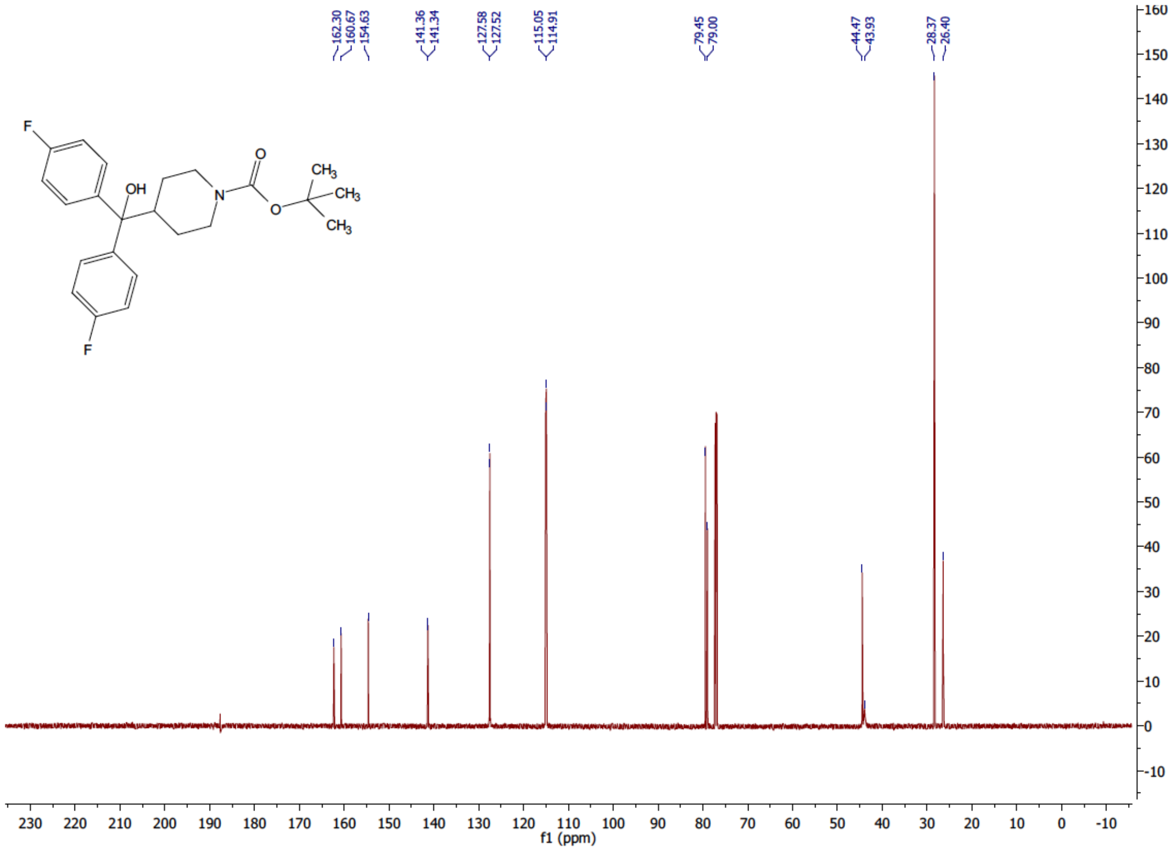

162.30
160.67
154.63
141.36
141.34
127.58
127.52
115.05
114.91
79.45
79.00
44.47
43.93
28.37
26.40
OH
N
O
O
CH_3
CH_3
CH_3
F
F
f1 (ppm)

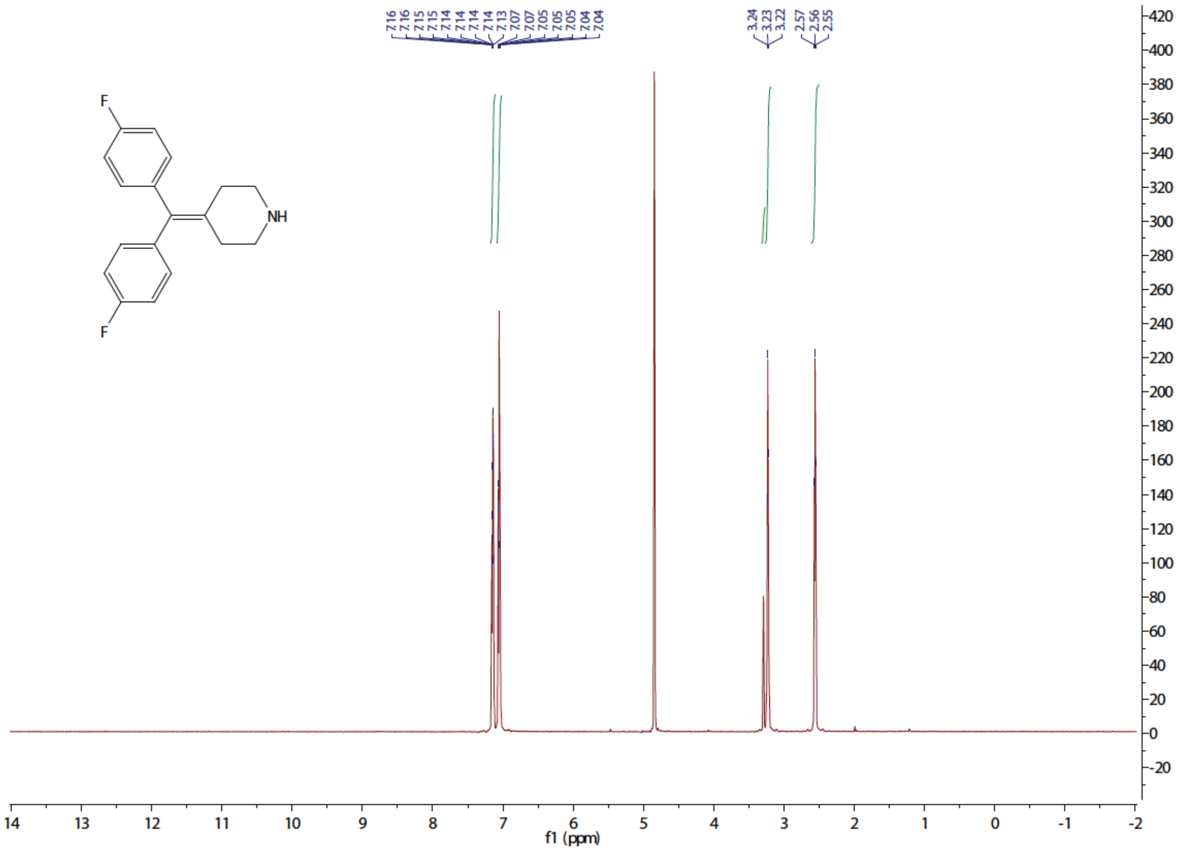

7.16
7.16
7.15
7.15
7.14
7.14
7.14
7.14
7.13
7.07
7.07
7.05
7.05
7.05
7.04
7.04
3.24
3.23
3.22
2.57
2.56
2.55
F
NH
F
f1 (ppm)

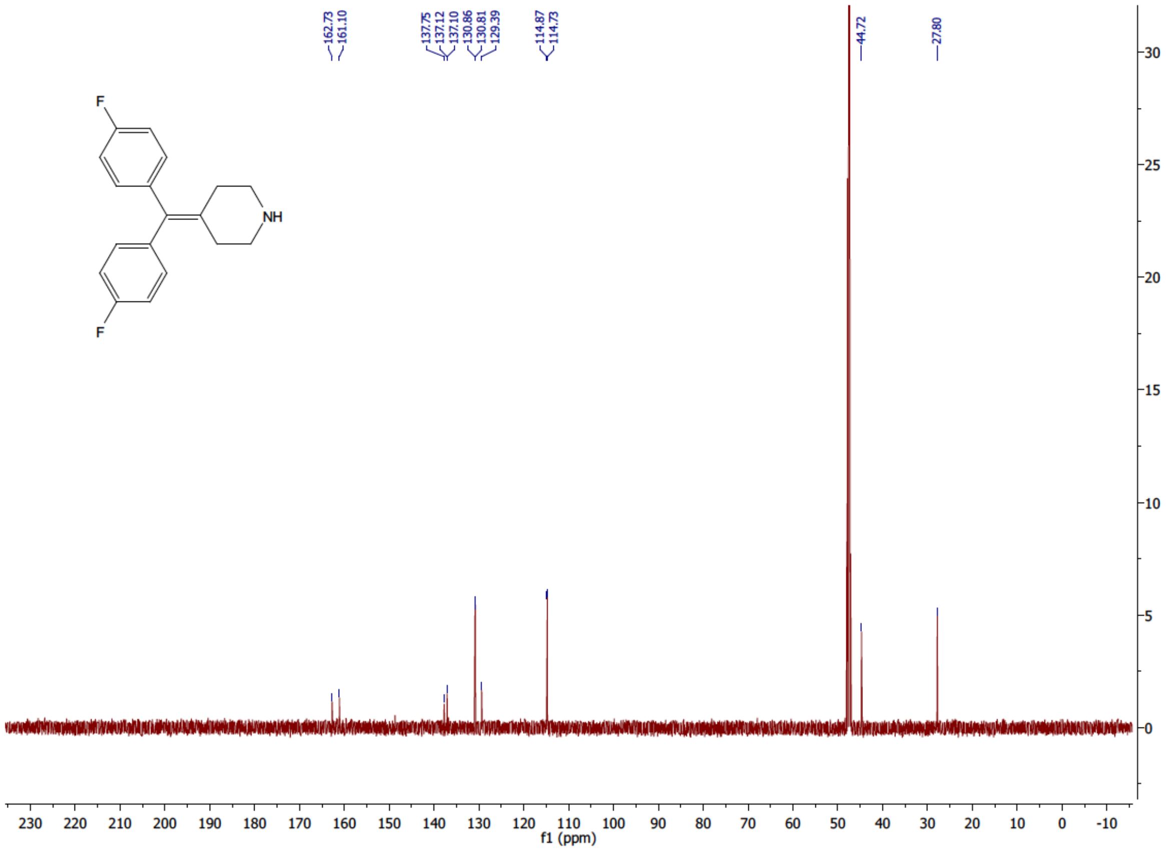

162.73
161.10
137.75
137.12
137.10
130.86
130.81
129.39
114.87
114.73
44.72
27.80
F
F
NH
f1 (ppm)

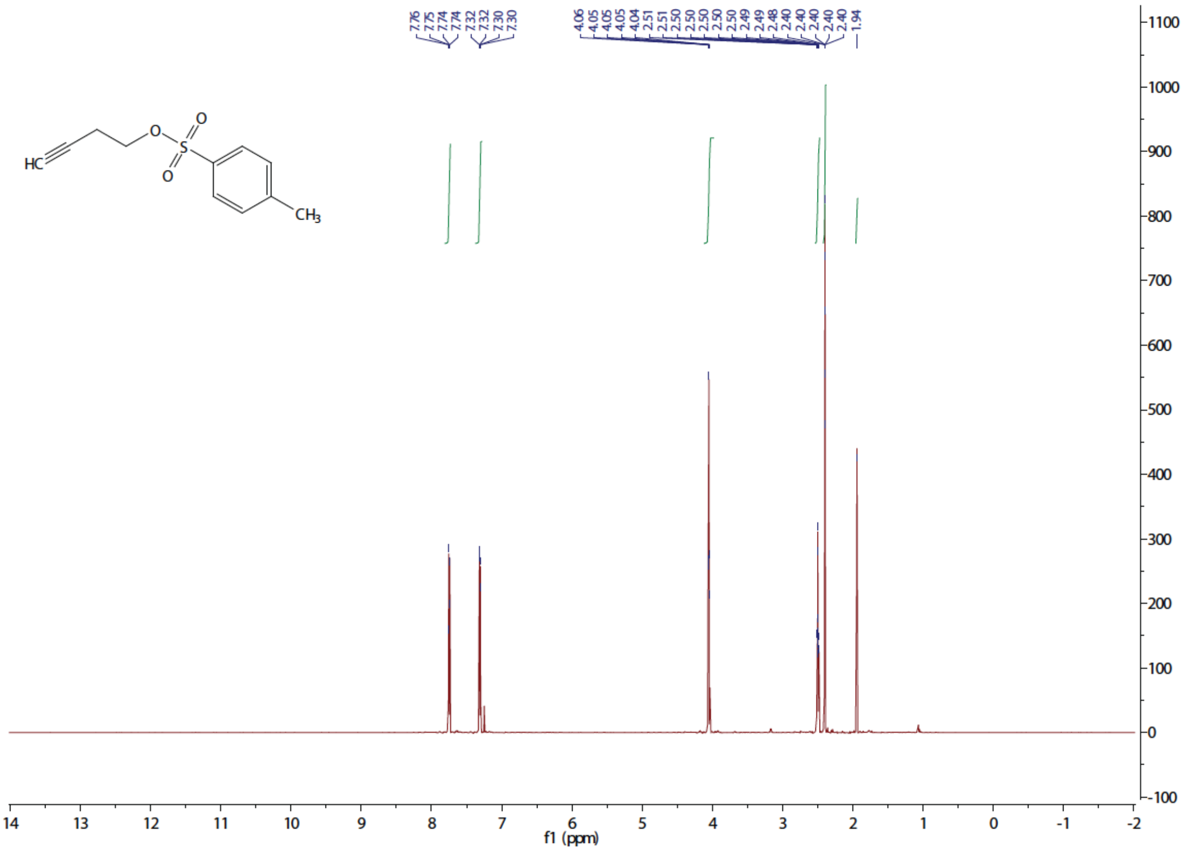

HC
O
S
O
O
CH3
7.76
7.75
7.74
7.74
7.32
7.32
7.30
7.30
4.06
4.05
4.05
4.05
4.04
2.51
2.51
2.50
2.50
2.50
2.50
2.49
2.49
2.48
2.40
2.40
2.40
2.40
1.94
f1 (ppm)

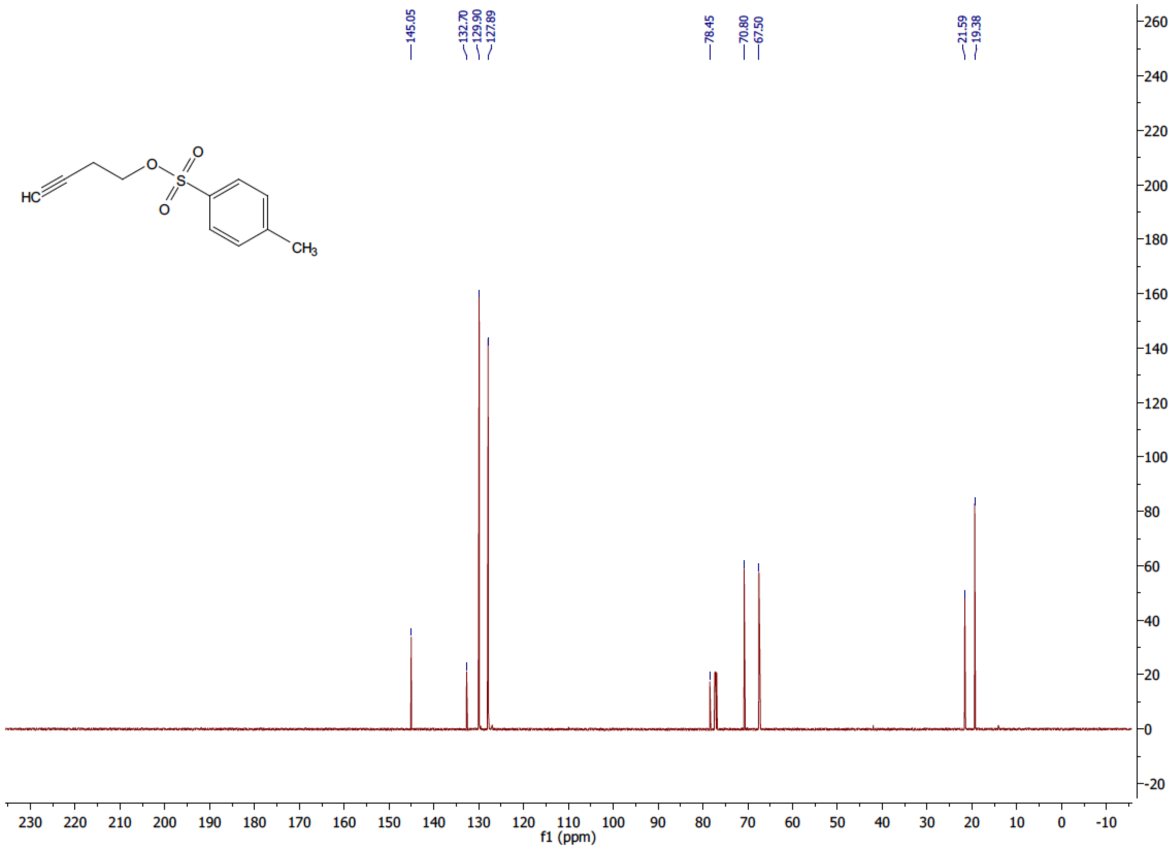

145.05
132.70
129.90
127.89
78.45
70.80
67.50
21.59
19.38
HC
O
S
O
O
CH3
f1 (ppm)

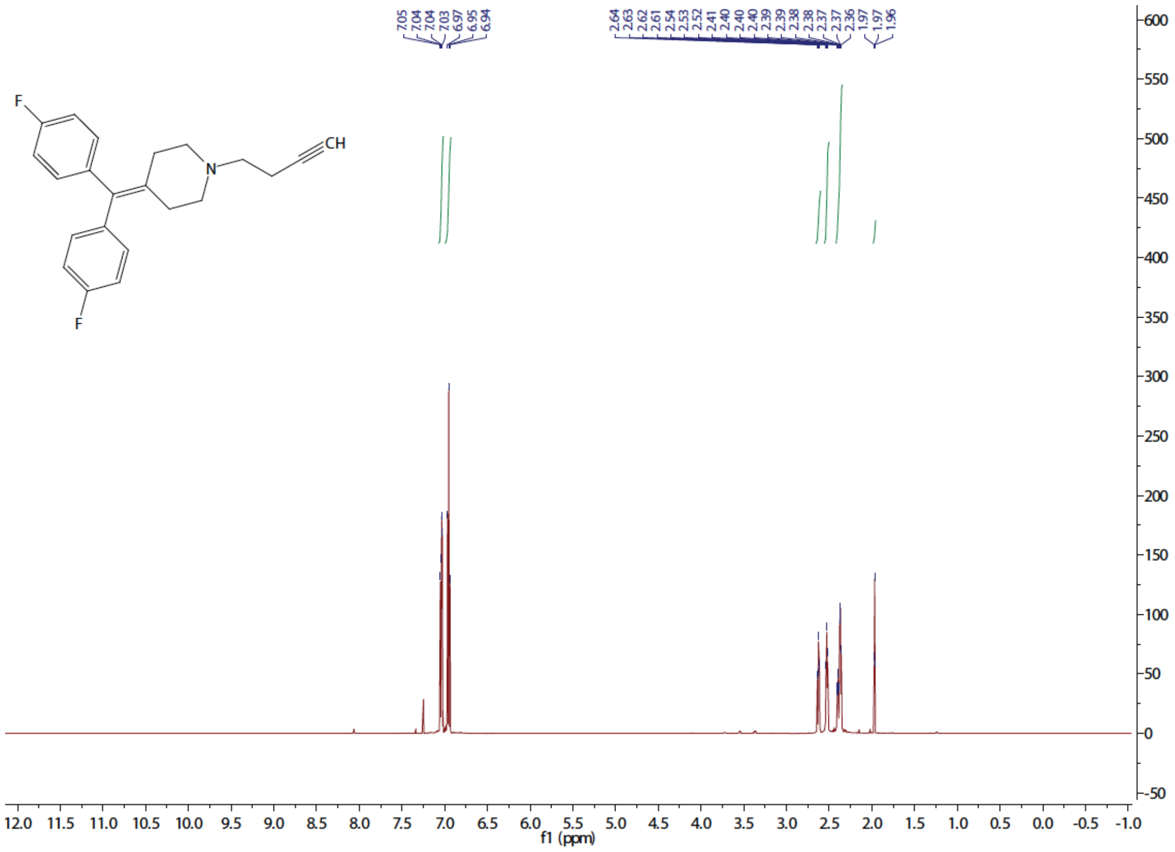

7.05
7.04
7.04
7.03
6.97
6.95
6.94
2.64
2.63
2.62
2.61
2.54
2.53
2.52
2.41
2.40
2.40
2.40
2.39
2.39
2.38
2.38
2.37
2.37
2.36
1.97
1.97
1.96
F
N
CH
F
f1 (ppm)

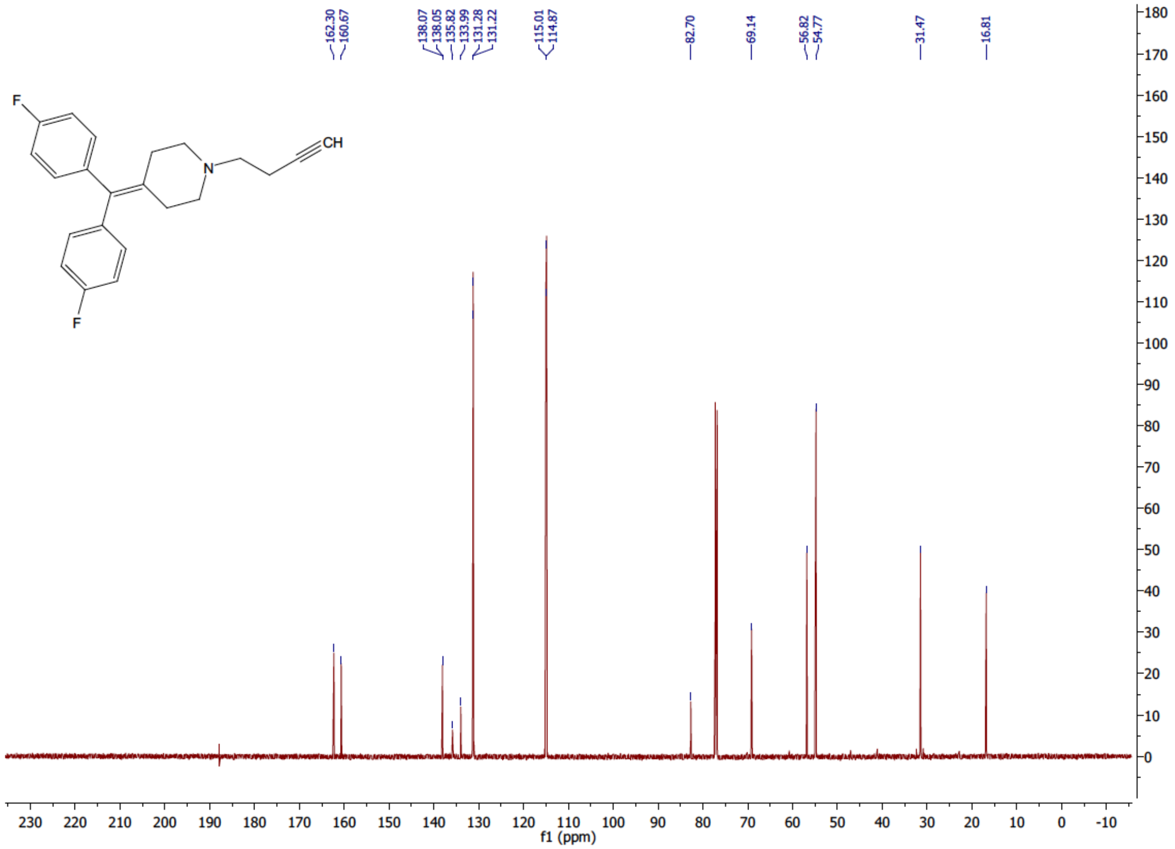
162.30
160.67
138.07
138.05
135.82
133.99
131.28
131.22
115.01
114.87
82.70
69.14
56.82
54.77
31.47
16.81
F
F
N
CH
f1 (ppm)

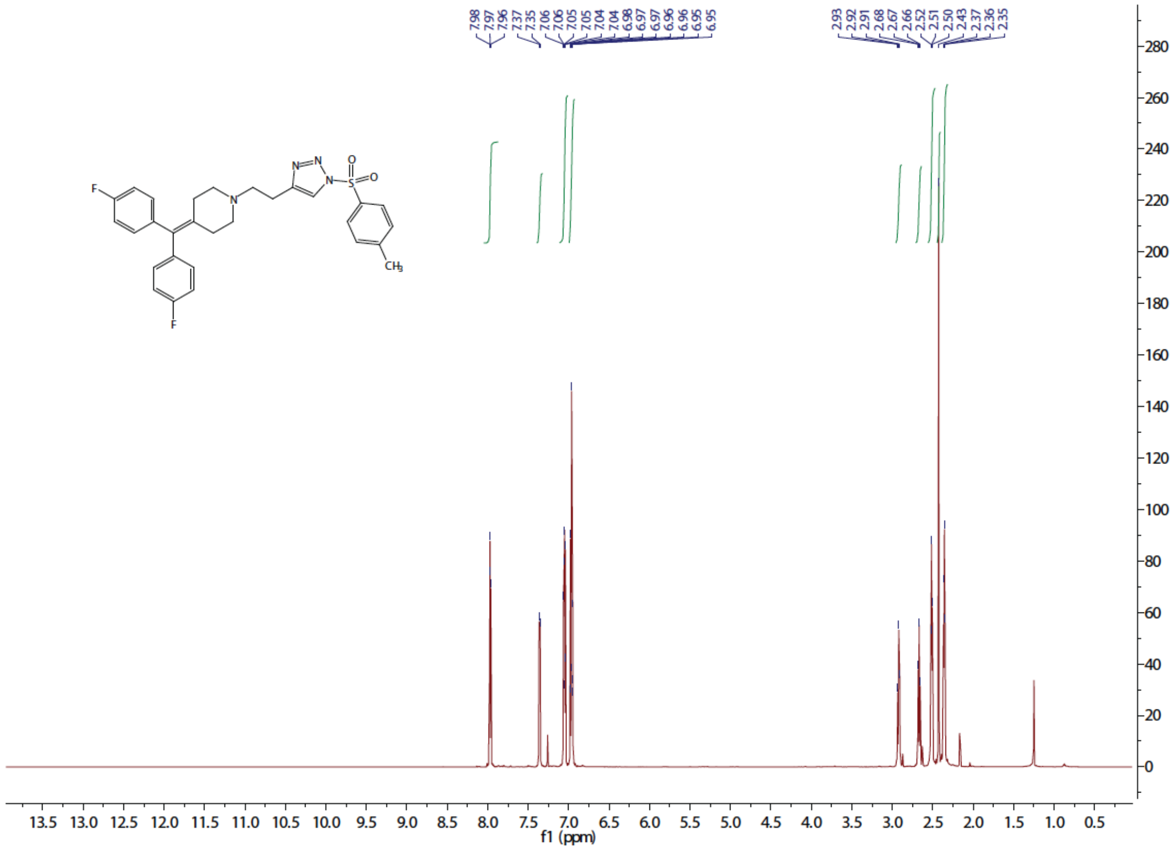

7.98 7.97 7.96 7.37 7.35 7.06 7.06 7.05 7.05 7.04 7.04 6.98 6.97 6.97 6.96 6.96 6.95 6.95
2.93 2.92 2.91 2.68 2.67 2.66 2.52 2.51 2.50 2.43 2.37 2.36 2.35
f1 (ppm)

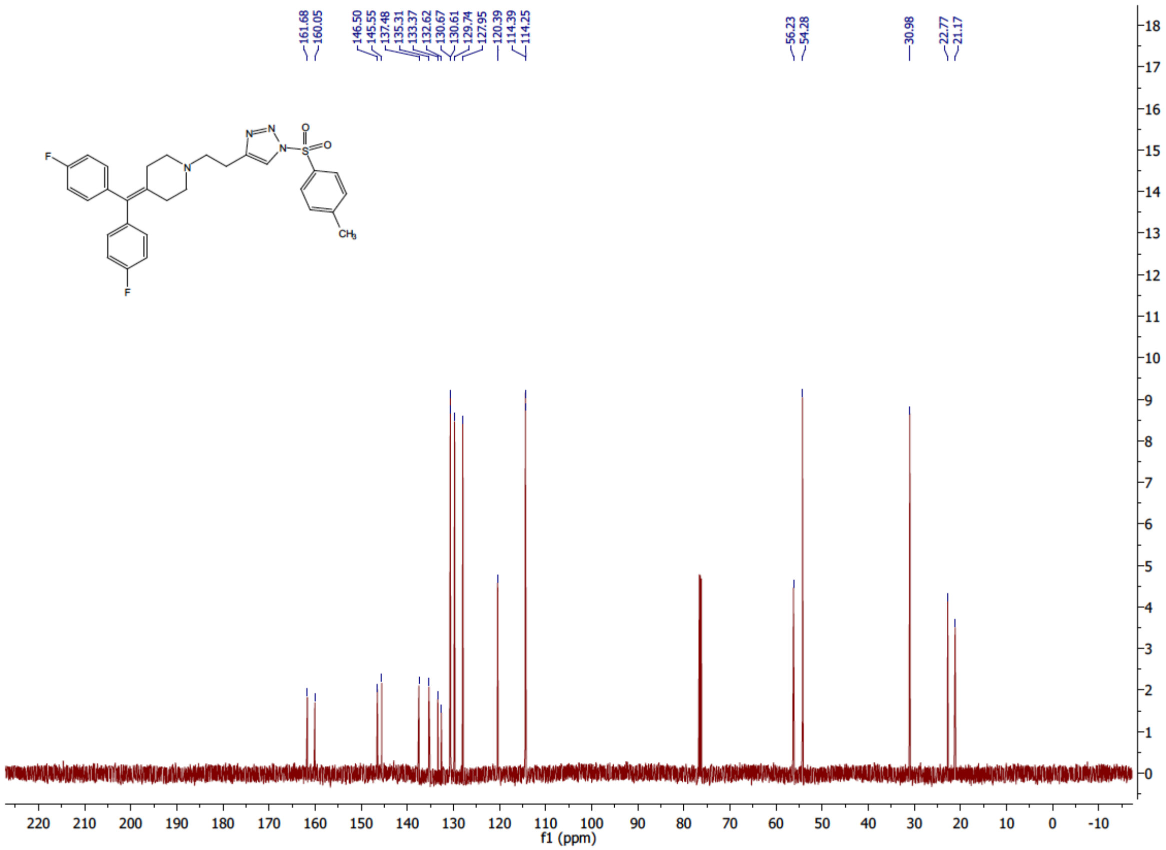

TH209CDCl3-H

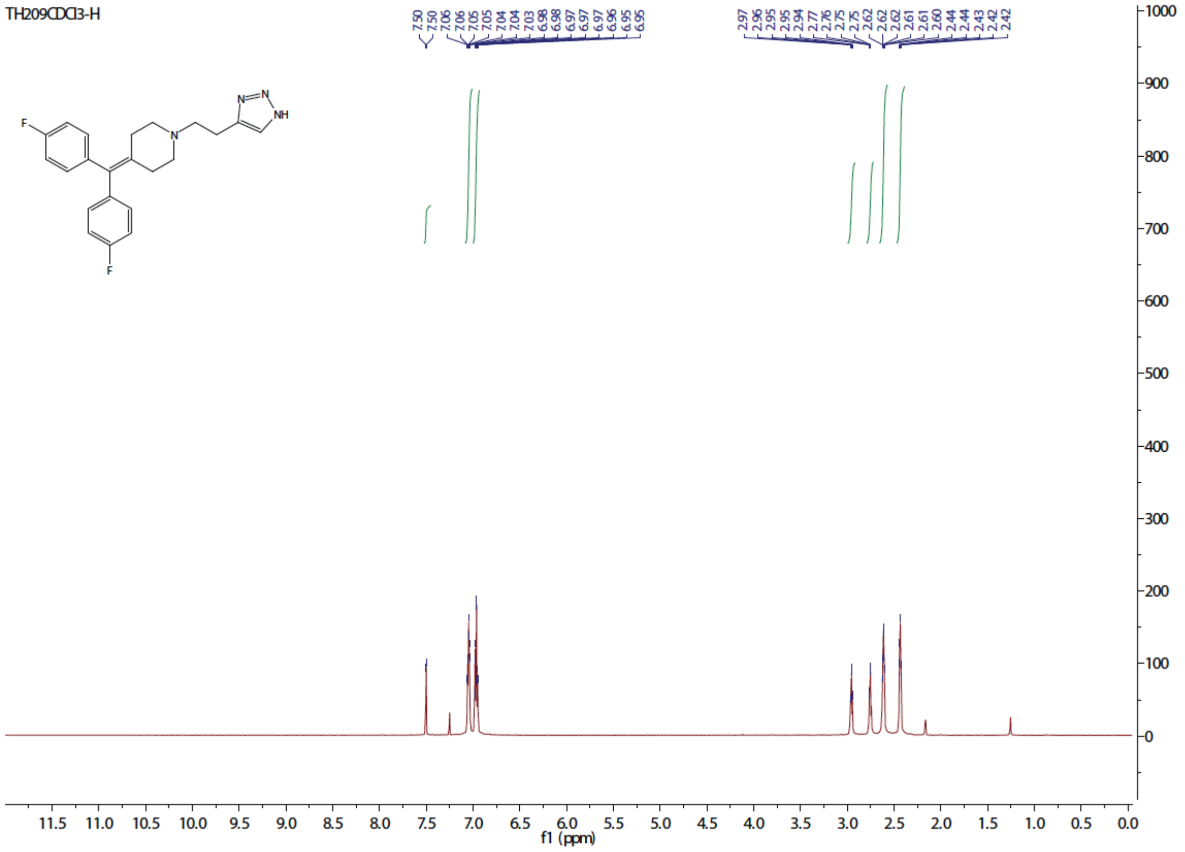

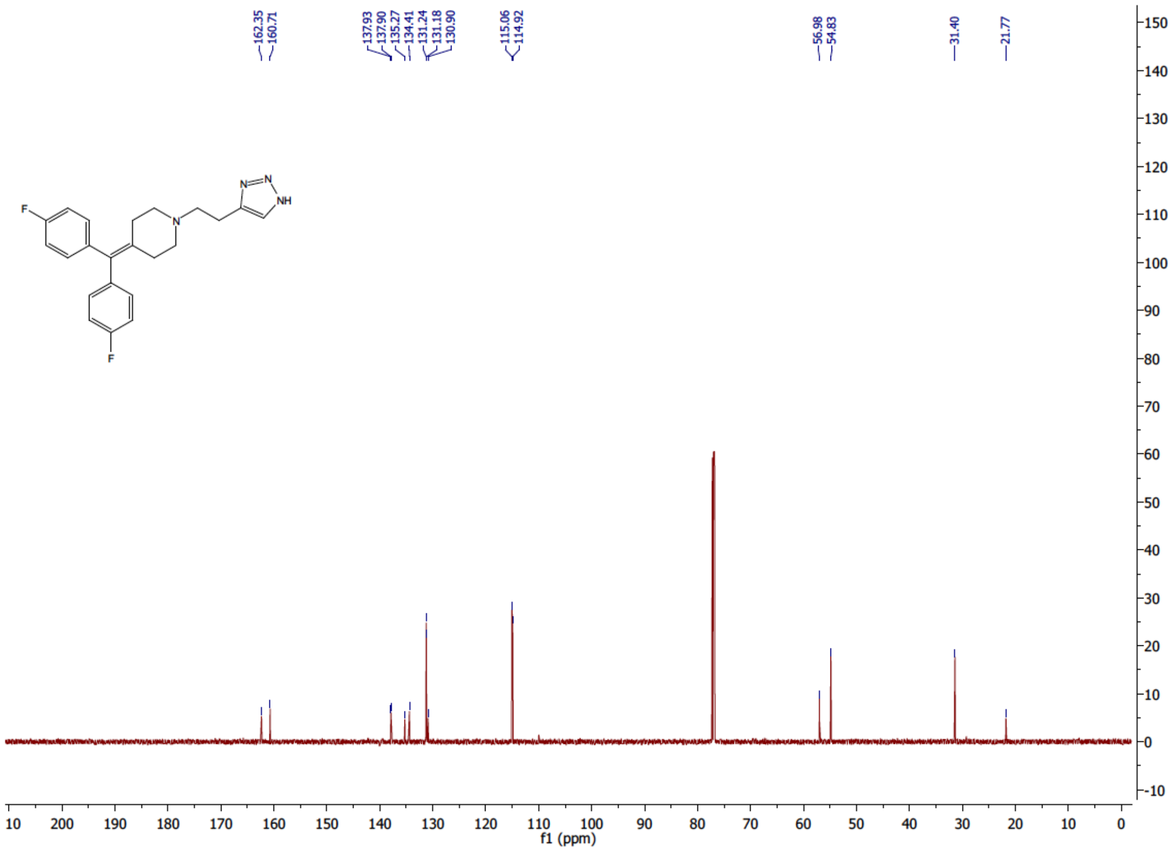
162.35
160.71
137.93
137.90
135.27
134.41
131.24
131.18
130.90
115.06
114.92
56.98
54.83
31.40
21.77
f1 (ppm)

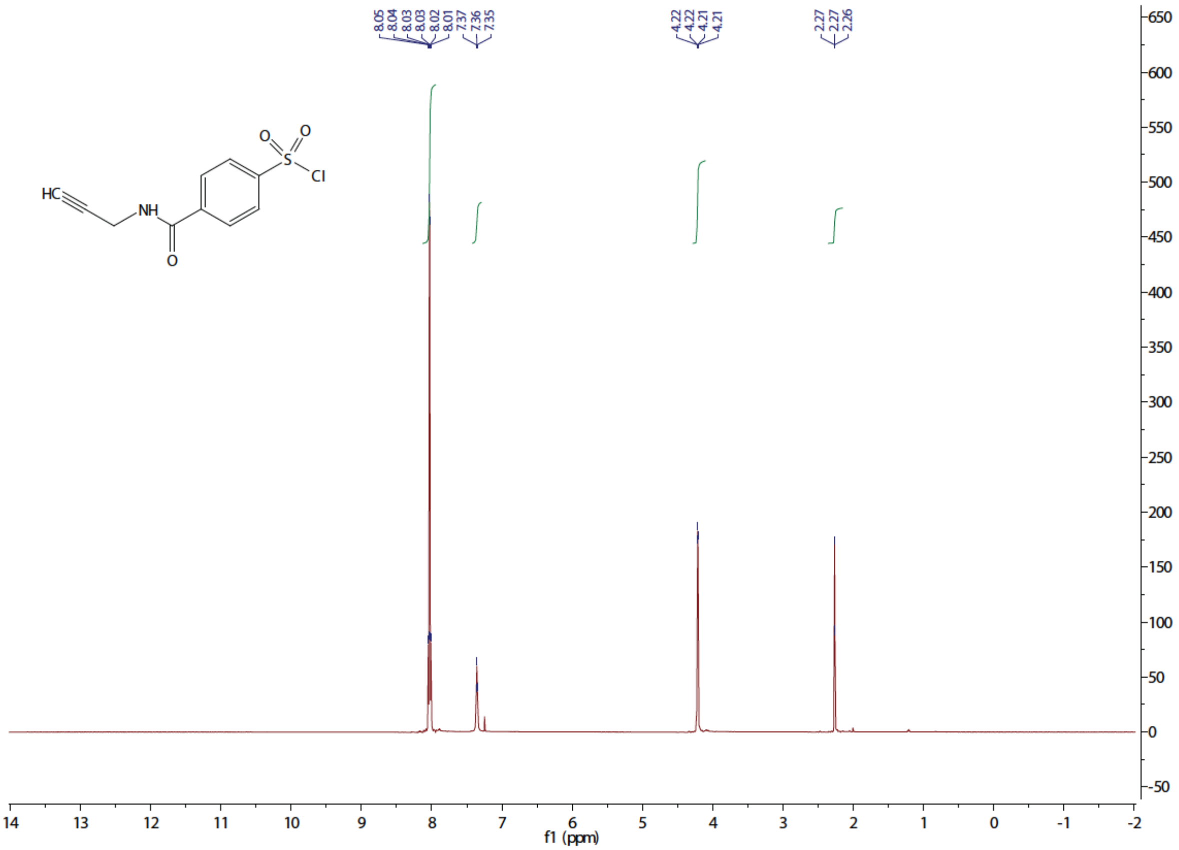

8.05
8.04
8.03
8.03
8.02
8.01
7.37
7.36
7.35
4.22
4.22
4.21
4.21
2.27
2.27
2.26
HC
NH
O
S
Cl
650
600
550
500
450
400
350
300
250
200
150
100
50
0
-50
14
13
12
11
10
9
8
7
6
5
4
3
2
1
0
-1
-2
f1 (ppm)

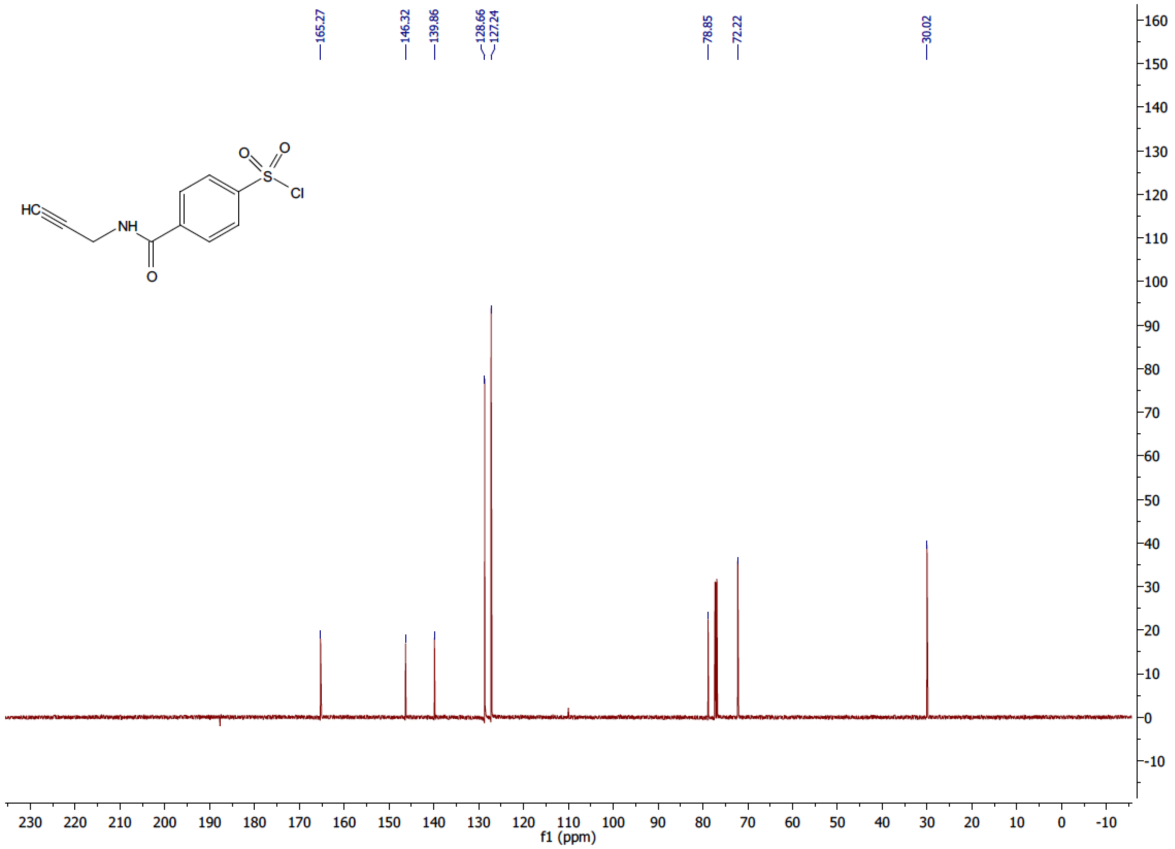

165.27
146.32
139.86
128.66
127.24
78.85
72.22
30.02
HC
NH
O
S
Cl
f1 (ppm)

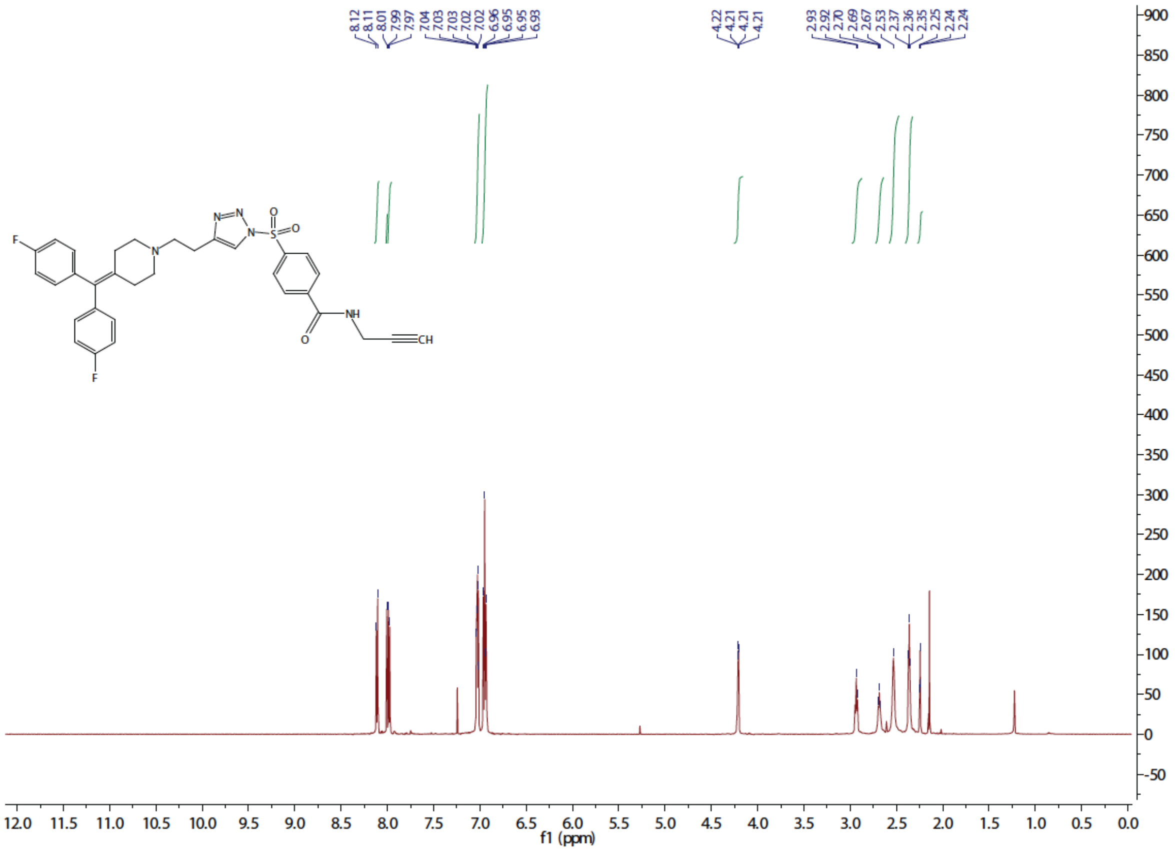

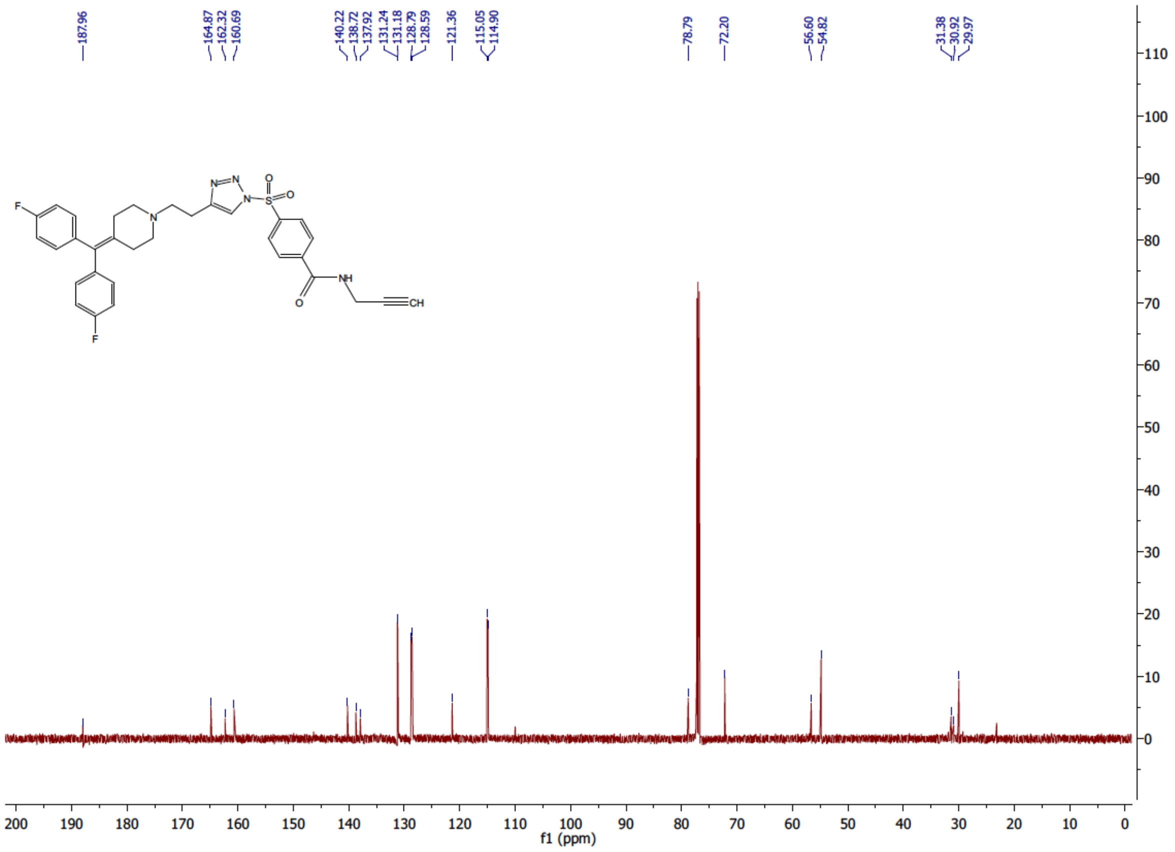
187.96
164.87
162.32
160.69
140.22
138.72
137.92
131.24
131.18
128.79
128.59
121.36
115.05
114.90
78.79
72.20
56.60
54.82
31.38
30.92
29.97
f1 (ppm)

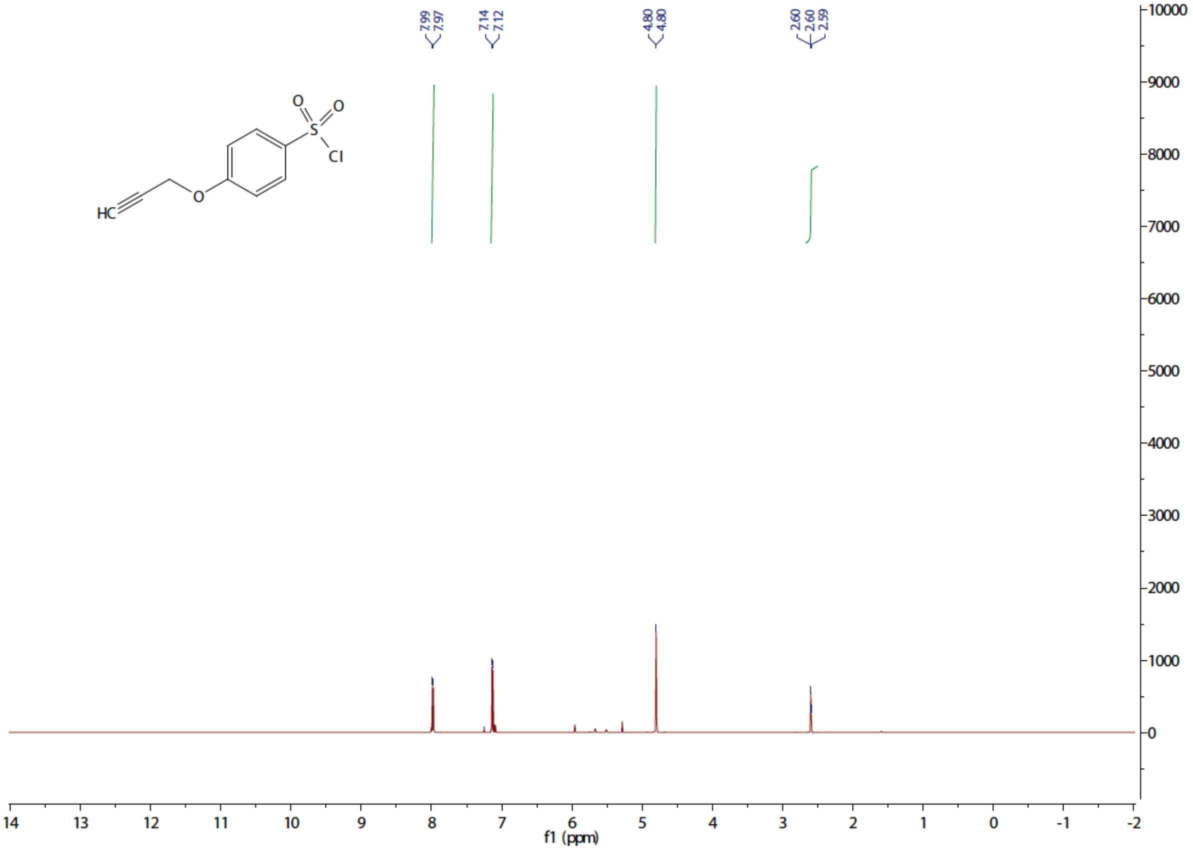

7.99
7.97
7.14
7.12
4.80
4.80
2.60
2.60
2.59
HC
O
S
O
O
Cl
10000
9000
8000
7000
6000
5000
4000
3000
2000
1000
0
14
13
12
11
10
9
8
7
6
5
4
3
2
1
0
-1
-2
f1 (ppm)

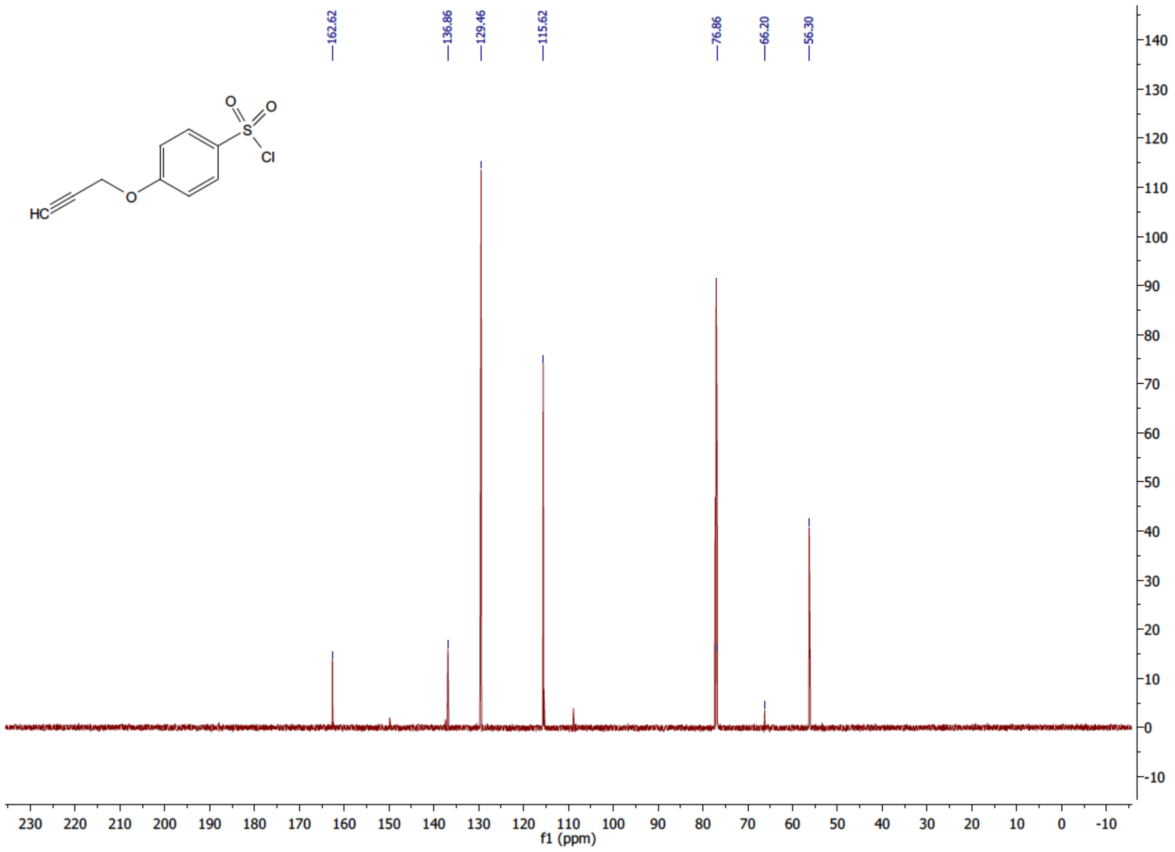

162.62
136.86
129.46
115.62
76.86
66.20
56.30
O
S
O
Cl
O
HC
f1 (ppm)

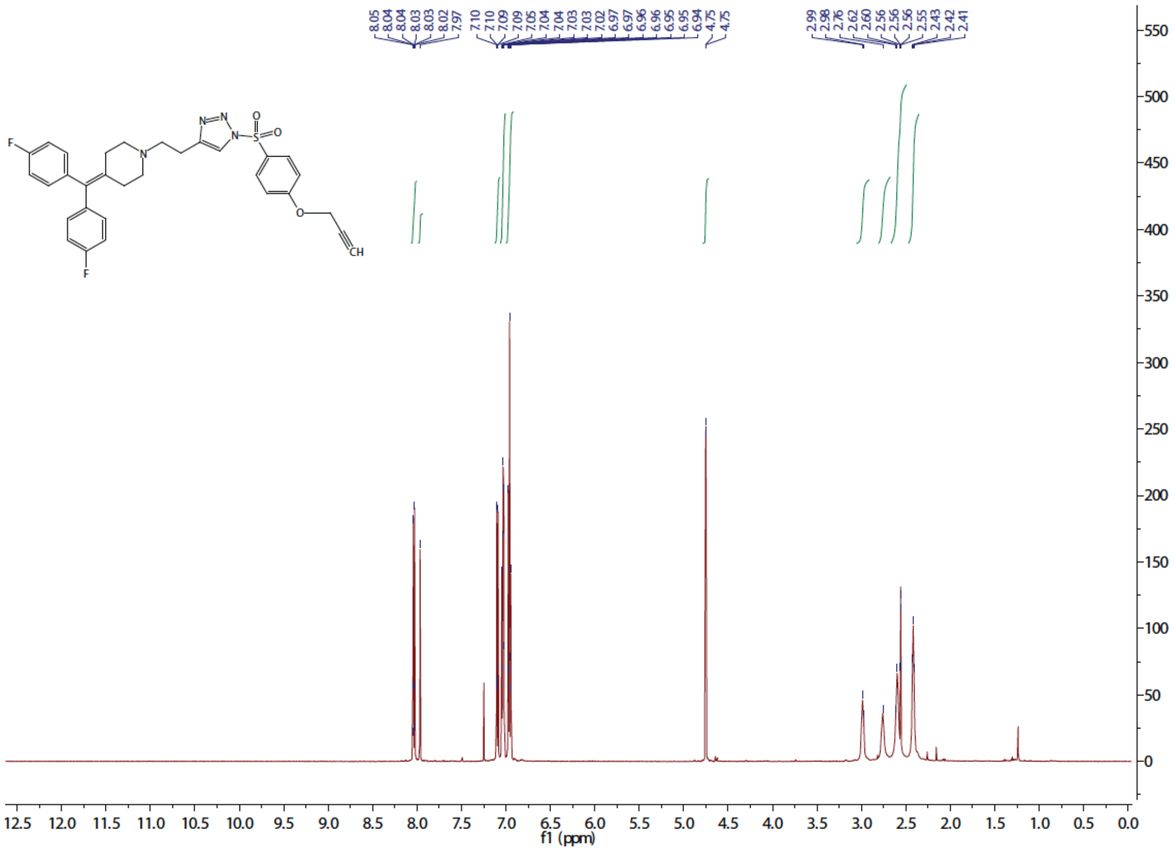
8.05 8.04 8.04 8.03 8.03 8.02 7.97
7.10 7.10 7.09 7.09 7.05 7.04 7.04 7.03 7.03 7.02 6.97 6.97 6.96 6.96 6.95 6.95 6.94 4.75 4.75
2.99 2.98 2.76 2.62 2.60 2.56 2.56 2.56 2.55 2.43 2.42 2.41
F
F
N
N=N
O
S
O
O
CH
550
500
450
400
350
300
250
200
150
100
50
0
12.5 12.0 11.5 11.0 10.5 10.0 9.5 9.0 8.5 8.0 7.5 7.0 6.5 6.0 5.5 5.0 4.5 4.0 3.5 3.0 2.5 2.0 1.5 1.0 0.5 0.0
f1 (ppm)

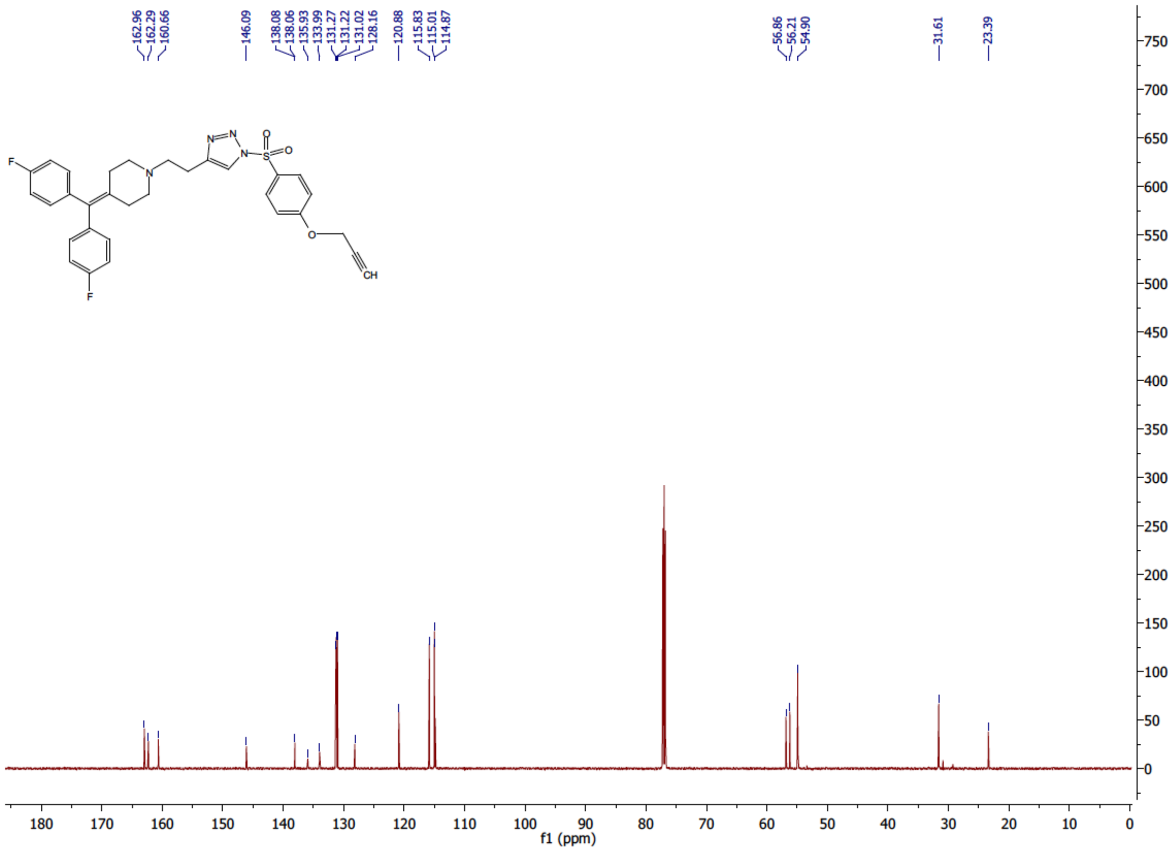

162.96
162.29
160.66
146.09
138.08
138.06
135.93
133.99
131.27
131.22
131.02
128.16
120.88
115.83
115.01
114.87
56.86
56.21
54.90
31.61
23.39
f1 (ppm)

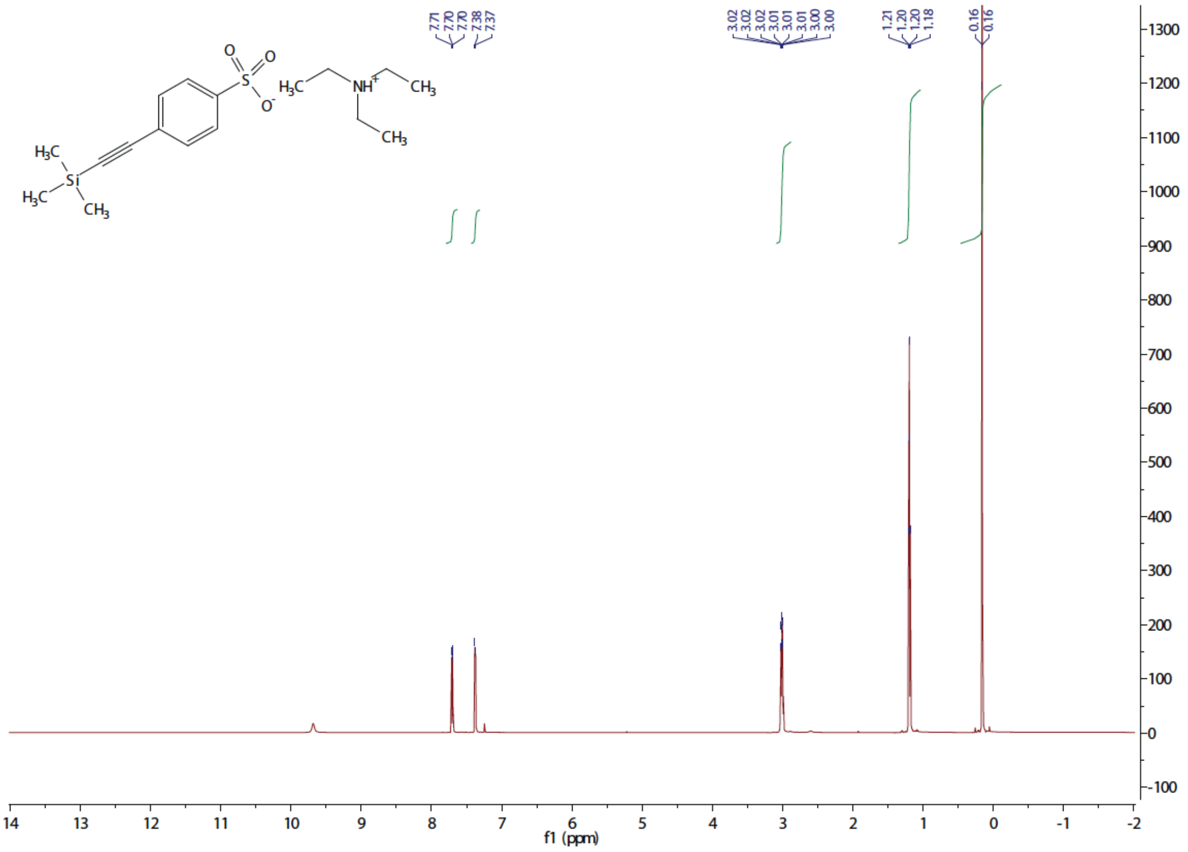

7.71
7.70
7.70
7.38
7.37
3.02
3.02
3.02
3.01
3.01
3.01
3.00
3.00
1.21
1.20
1.20
1.18
0.16
0.16
f1 (ppm)

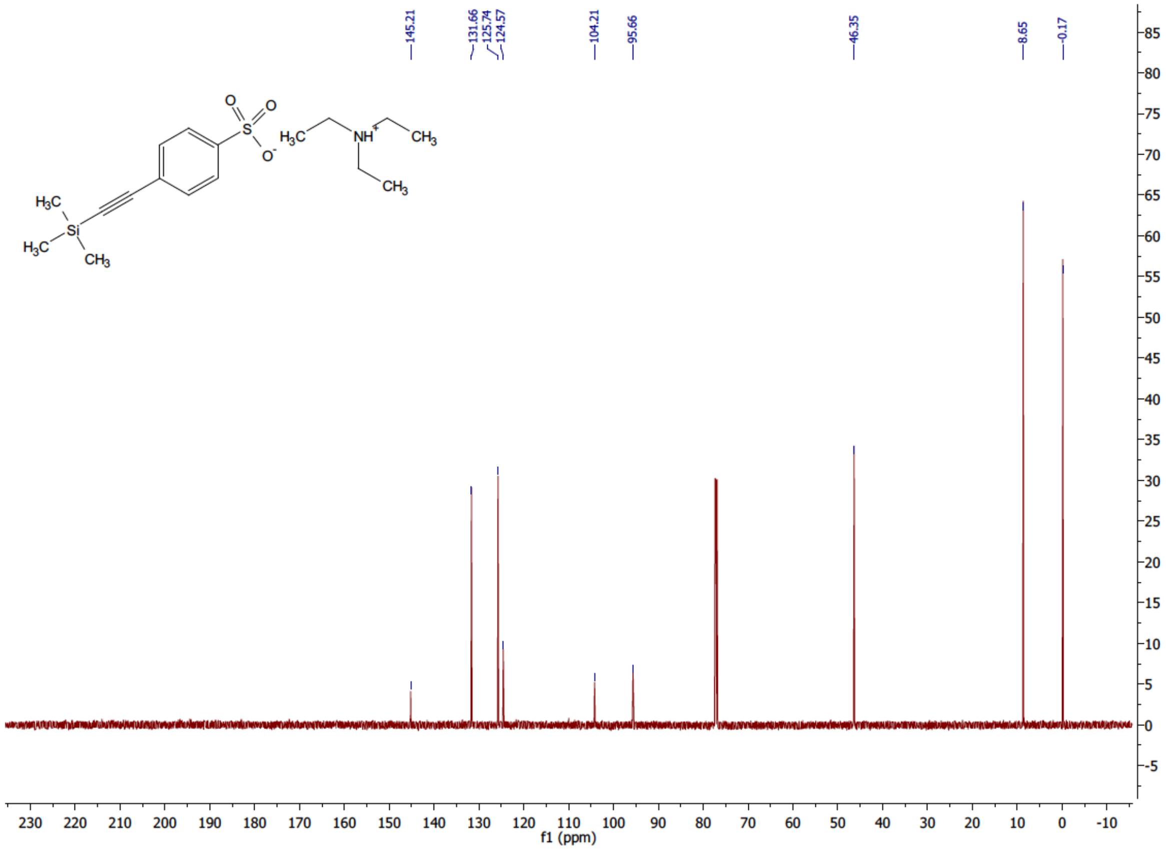
145.21
131.66
125.74
124.57
104.21
95.66
46.35
8.65
0.17
f1 (ppm)

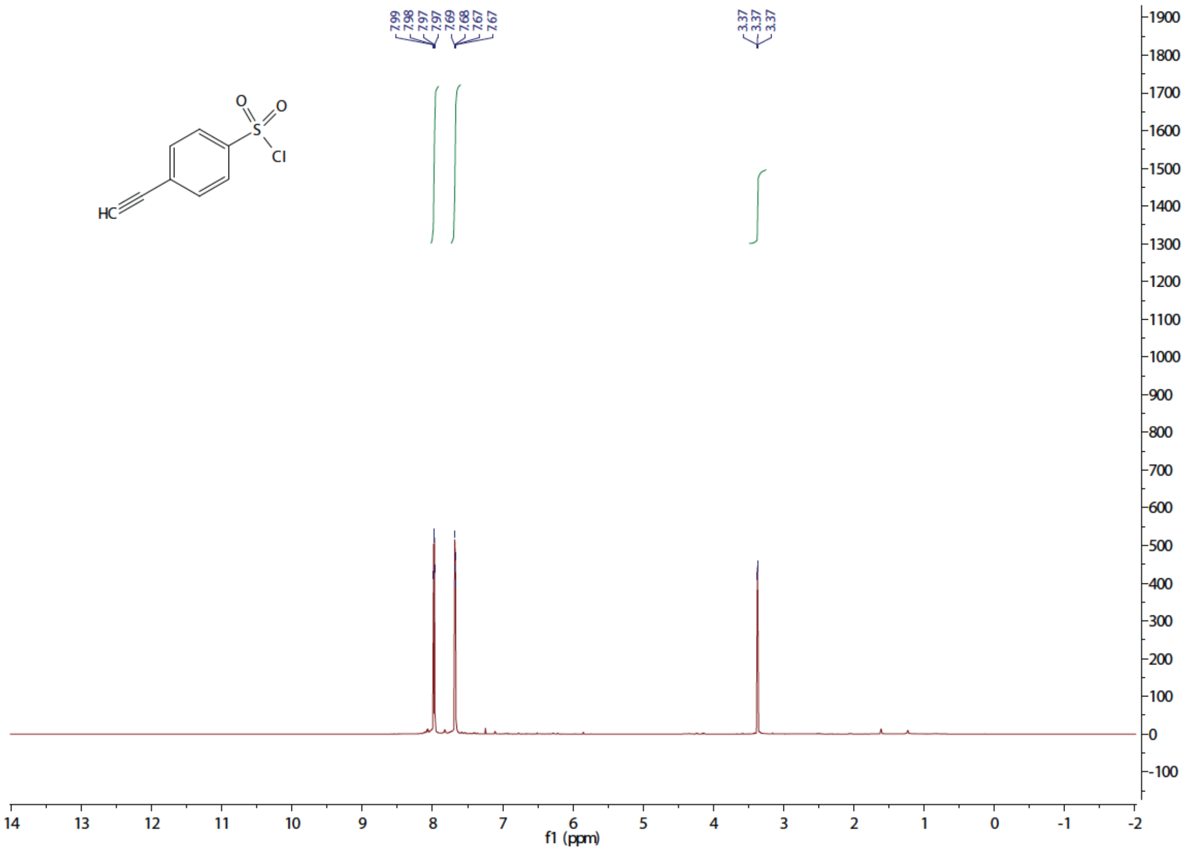

7.99
7.98
7.97
7.97
7.69
7.68
7.67
7.67
3.37
3.37
3.37
O
O
S
Cl
HC
f1 (ppm)

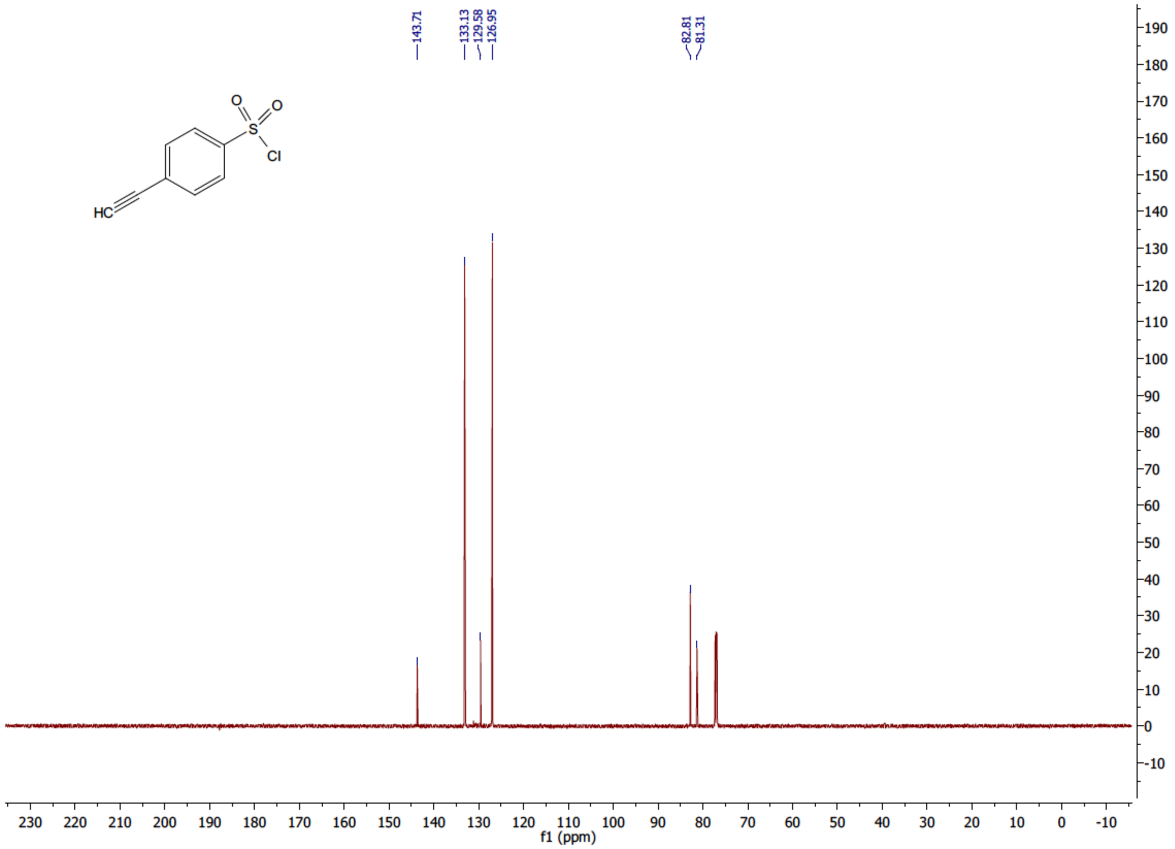
143.71
133.13
129.58
126.95
82.81
81.31
HC
O
O
S
Cl
f1 (ppm)

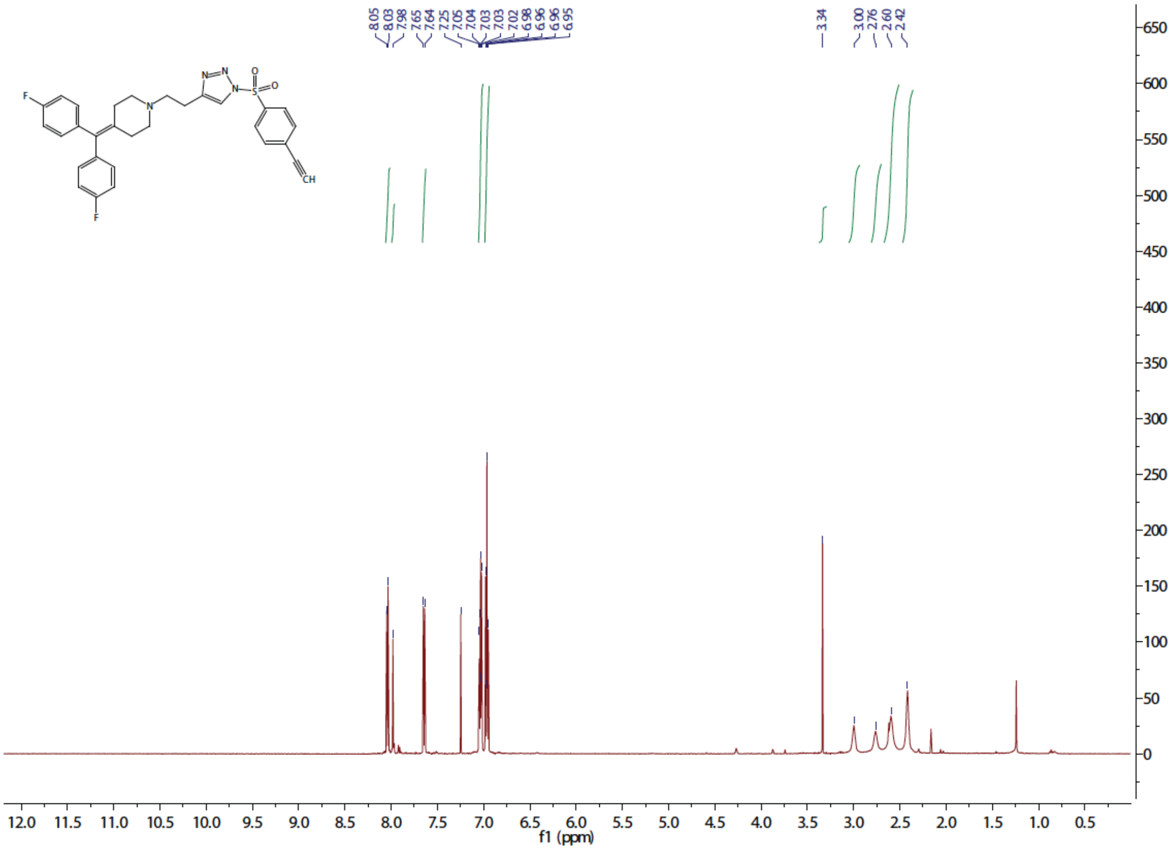

8.05
8.03
7.98
7.65
7.64
7.25
7.05
7.04
7.03
7.03
7.02
6.98
6.96
6.96
6.95
3.34
3.00
2.76
2.60
2.42
f1 (ppm)

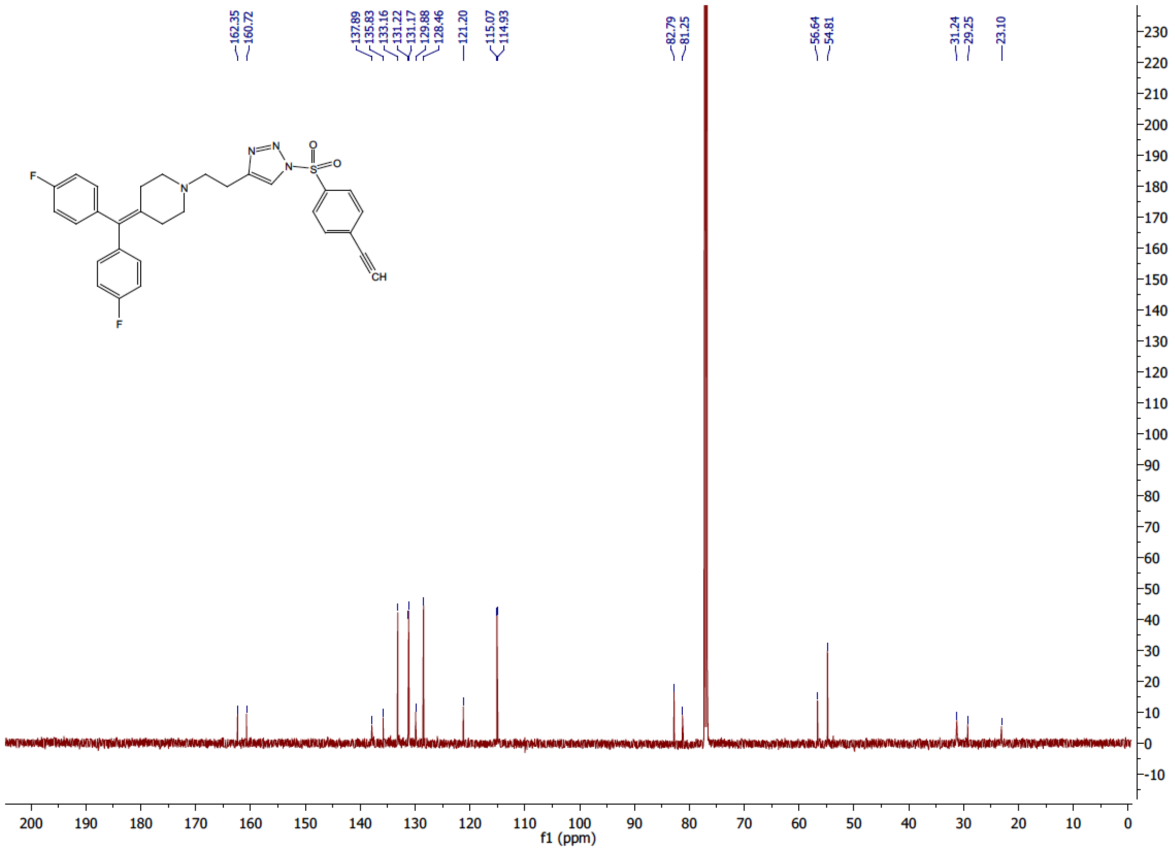
162.35
160.72
137.89
135.83
133.16
131.22
131.17
129.88
128.46
121.20
115.07
114.93
82.79
81.25
56.64
54.81
31.24
29.25
23.10
f1 (ppm)

5.2 MS/MS Spectra of probe-modified peptides

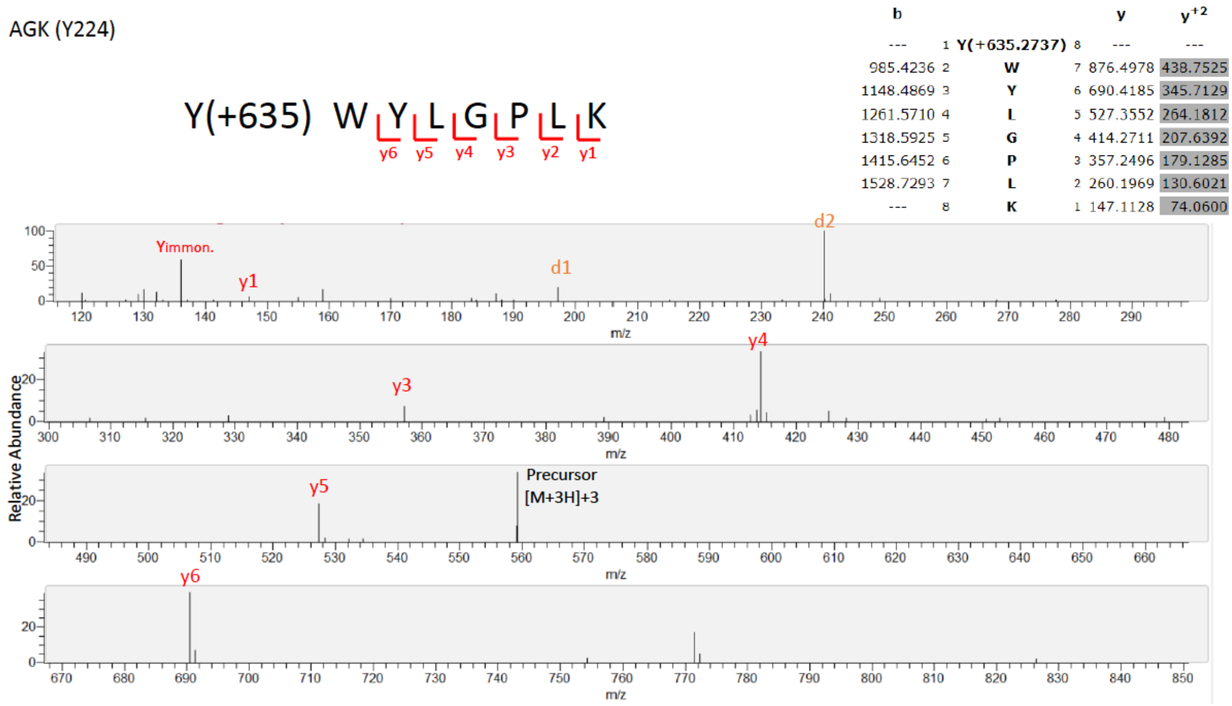

b				y	y^{+2}
---	1	Y(+635.2737)	8	---	---
985.4236	2	W	7	876.4978	438.7525
1148.4869	3	Y	6	690.4185	345.7129
1261.5710	4	L	5	527.3552	264.1812
1318.5925	5	G	4	414.2711	207.6392
1415.6452	6	P	3	357.2496	179.1285
1528.7293	7	L	2	260.1969	130.6021
---	8	K	1	147.1128	74.0600

PI42C (Y384)

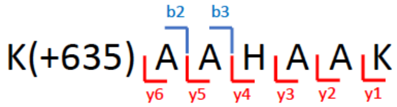

b	b^{+2}				y	y^{+2}
---	---	1	K(+635.2737)	7	---	---
835.4131	418.2102	2	A	6	568.3202	284.6637
906.4502	453.7287	3	A	5	497.2831	249.1452
1043.5091	522.2582	4	H	4	426.2459	213.6266
1114.5462	557.7767	5	A	3	289.1870	145.0972
1185.5833	593.2953	6	A	2	218.1499	109.5786
---	---	7	K	1	147.1128	74.0600

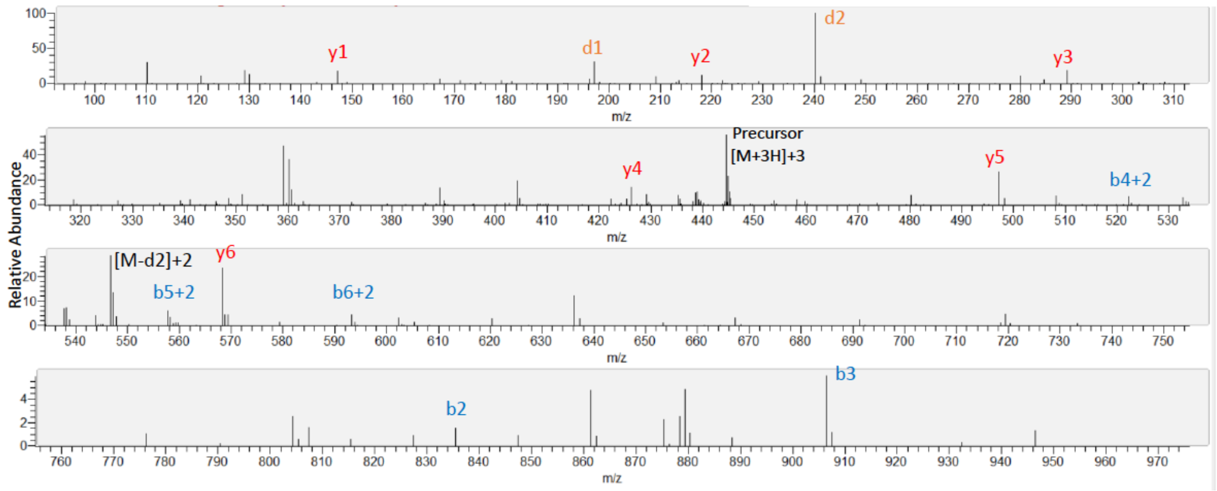

KT3K (K45)

b	b^{+2}				y	y^{+2}
---	---	1	V	8	---	---
214.1186	---	2	N	7	1420.7001	710.8537
311.1714	---	3	P	6	1306.6572	653.8322
1074.5400	537.7737	4	K(+635.2737)	5	1209.6044	605.3059
1145.5772	573.2922	5	A	4	446.2358	223.6215
1274.6198	637.8135	6	E	3	375.1987	188.1030
1345.6569	673.3321	7	A	2	246.1561	123.5817
---	---	8	R	1	175.1190	88.0631

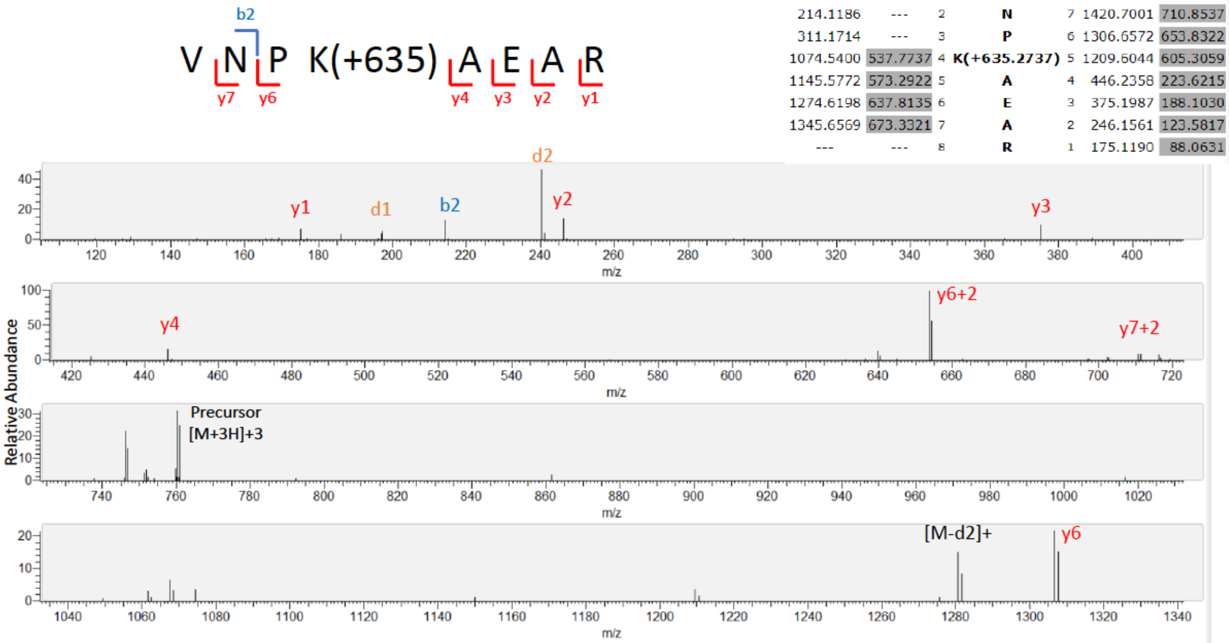

PRPK (Y67)

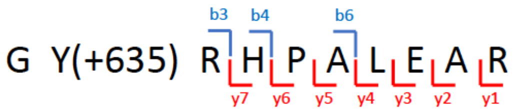

b	b^{+2}				y	y^{+2}
---	---	1	G	10	---	---
856.3658	---	2	Y(+635.2737)	9	1747.8697	874.4385
1012.4669	506.7371	3	R	8	949.5326	475.2700
1149.5258	575.2665	4	H	7	793.4315	397.2194
1246.5786	623.7929	5	P	6	656.3726	328.6899
1317.6157	659.3115	6	A	5	559.3198	280.1636
1430.6997	715.8535	7	L	4	488.2827	244.6450
1559.7423	780.3748	8	E	3	375.1987	188.1030
1630.7794	815.8934	9	A	2	246.1561	123.5817
---	---	10	R	1	175.1190	88.0631

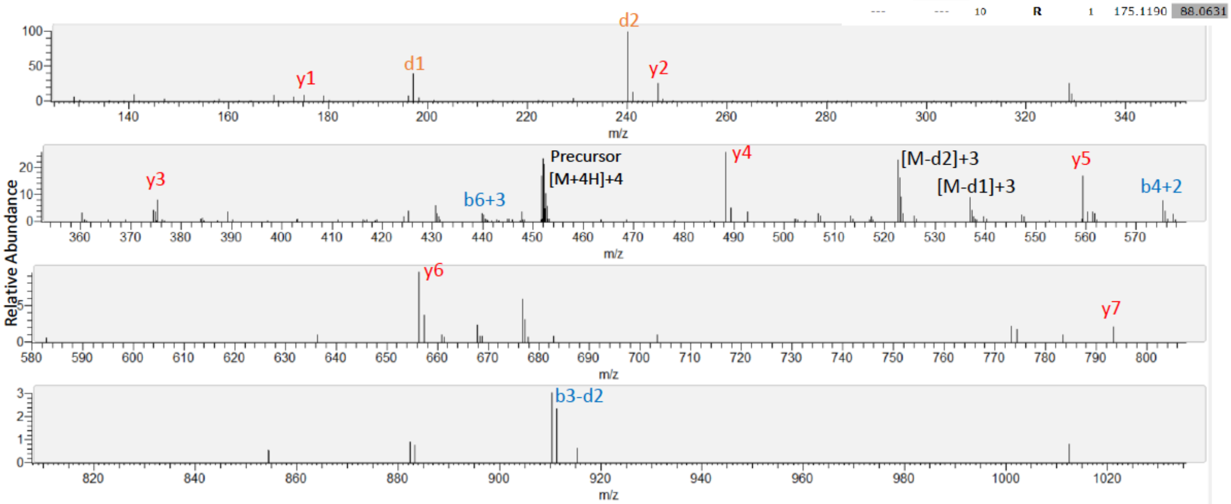

MK03 (Y280)

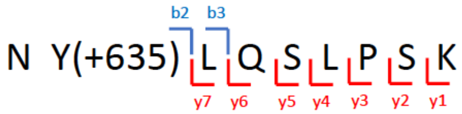

b				y	y^{+2}
---	1	**N**	9	---	---
913.3872	2	**Y(+635.2737)**	8	1570.7934	785.9003
1026.4713	3	**L**	7	772.4563	386.7318
1154.5299	4	**Q**	6	659.3723	330.1898
1241.5619	5	**S**	5	531.3137	266.1605
1354.6460	6	**L**	4	444.2817	222.6445
1451.6987	7	**P**	3	331.1976	166.1024
1538.7308	8	**S**	2	234.1448	117.5761
---	9	**K**	1	147.1128	74.0600

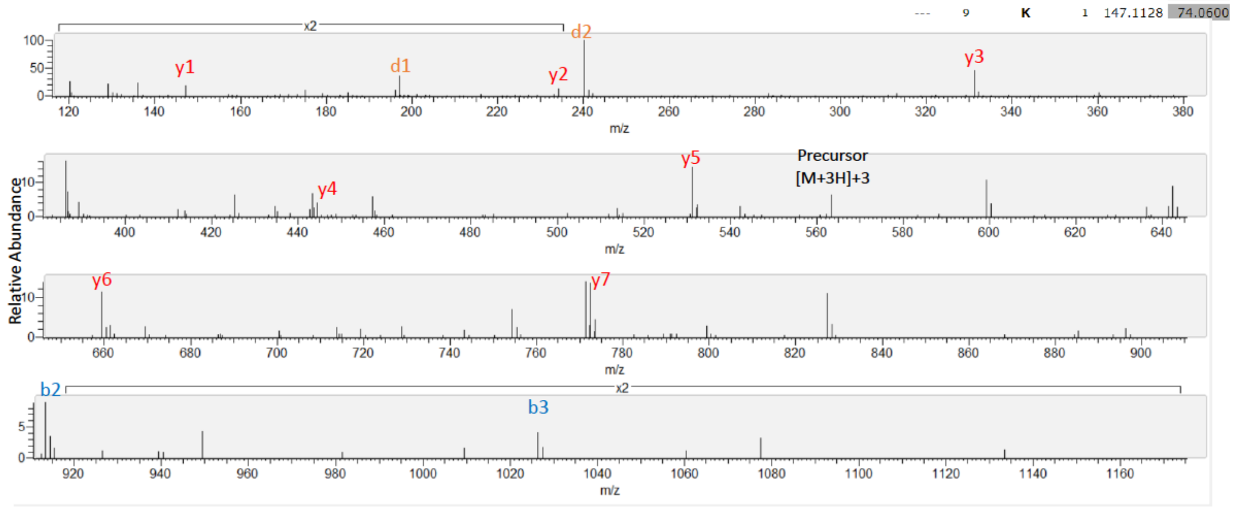

DGKA (Y22)

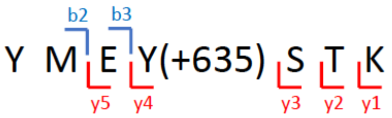

b				y	y^{+2}
---	1	Y	7	---	---
295.1111	2	M	6	1393.6126	697.3099
424.1537	3	E	5	1262.5721	631.7897
1222.4907	4	Y(+635.2737)	4	1133.5295	567.2684
1309.5227	5	S	3	335.1925	168.0999
1410.5704	6	T	2	248.1605	124.5839
---	7	K	1	147.1128	74.0600

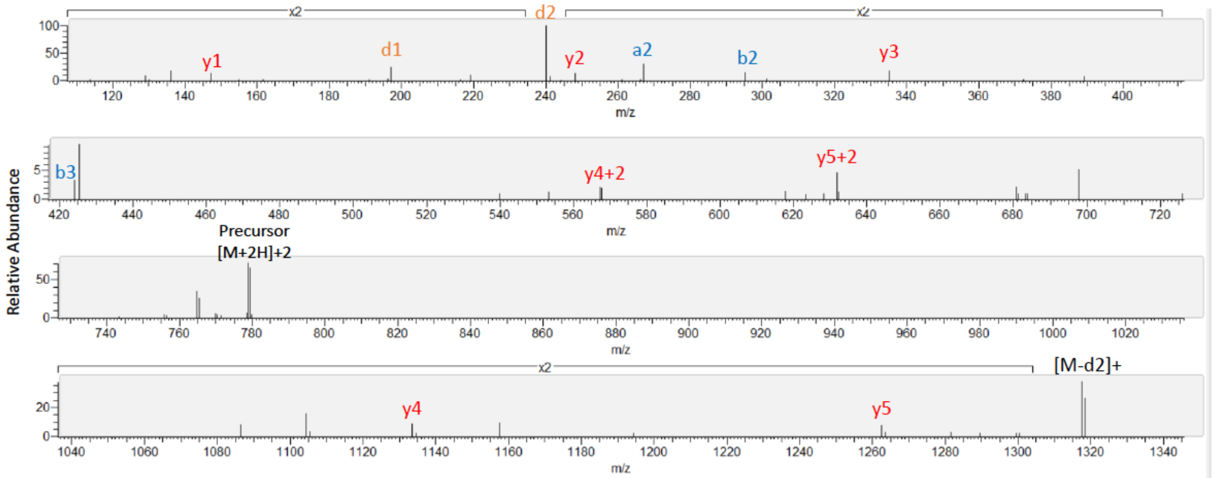

DGKA (Y544)

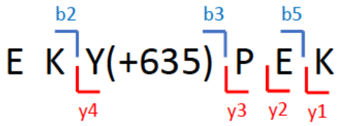

b	b^{+2}				y	y^{+2}
---	---	1	E	6	---	---
258.1448	129.5761	2	K	5	1299.6402	650.3237
1056.4819	528.7446	3	Y(+635.2737)	4	1171.5452	586.2762
1153.5346	577.2710	4	P	3	373.2082	187.1077
1282.5772	641.7922	5	E	2	276.1554	138.5813
---	---	6	K	1	147.1128	74.0600

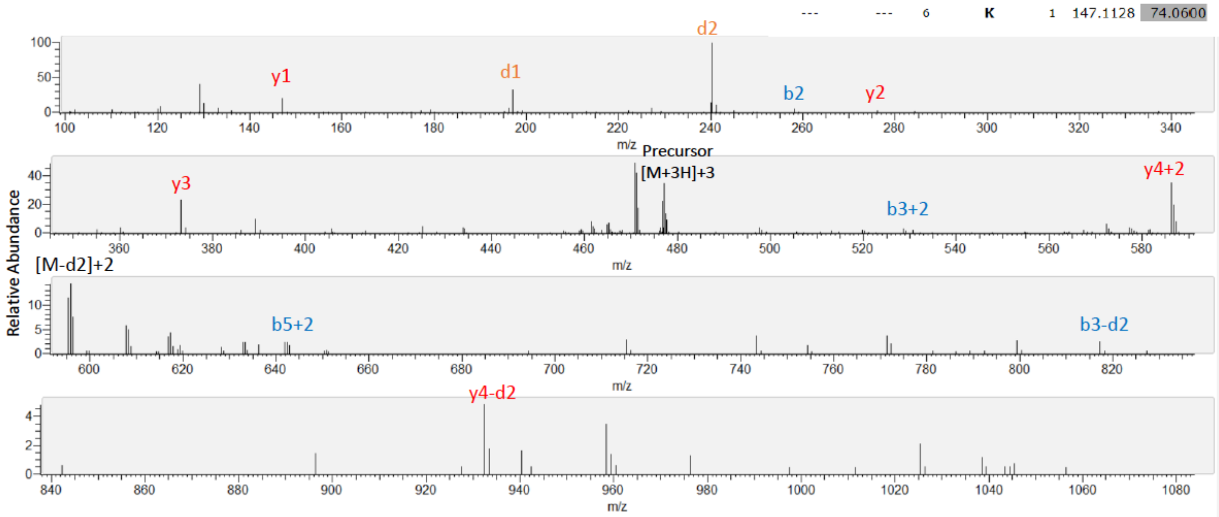

DGKA (Y240)

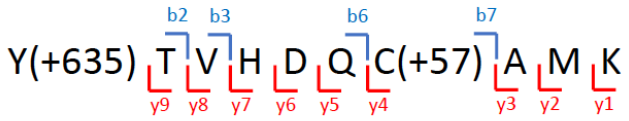

b	b^{+2}				y	y^{+2}
---	---	1	**Y(+635.2737)**	10	---	---
900.3920	---	2	**T**	9	1089.4816	545.2444
999.4604	---	3	**V**	8	988.4339	494.7206
1136.5193	568.7633	4	**H**	7	889.3655	445.1864
1251.5463	626.2768	5	**D**	6	752.3066	376.6569
1379.6048	690.3061	6	**Q**	5	637.2796	319.1435
1539.6355	770.3214	7	**C(+57.02146)**	4	509.2210	255.1142
1610.6726	805.8399	8	**A**	3	349.1904	175.0988
1741.7131	871.3602	9	**M**	2	278.1533	139.5803
---	---	10	**K**	1	147.1128	74.0600

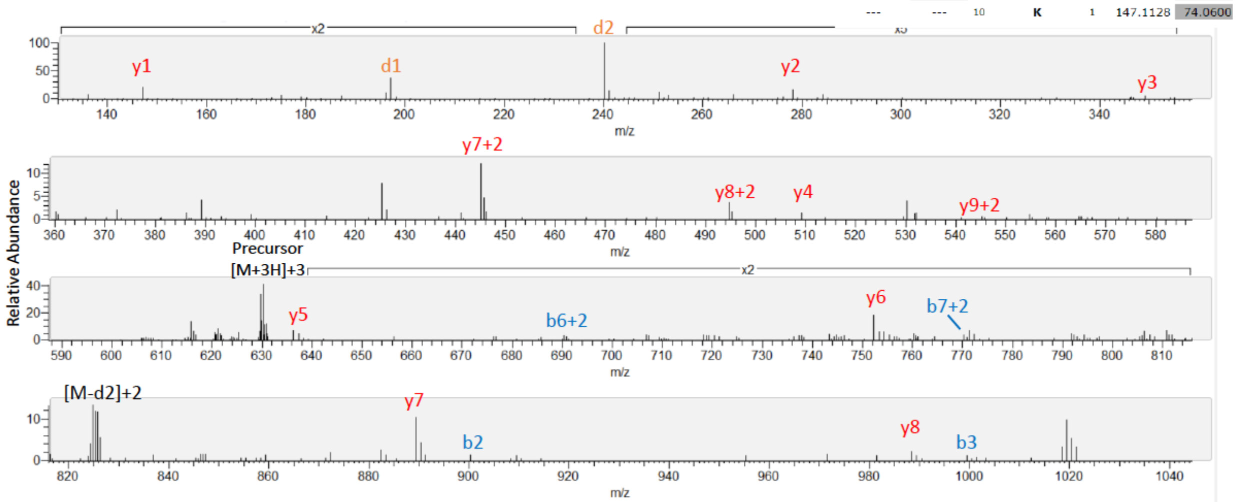

DGKA (Y623)

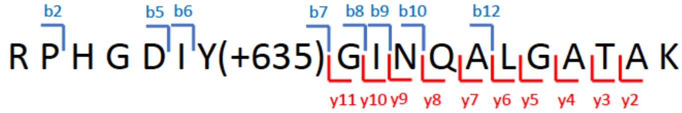

b	b^{+2}				y	y^{+2}
---	---	1	R	18	---	---
254.1612	127.5842	2	P	17	2361.1656	1181.0864
391.2201	196.1137	3	H	16	2264.1128	1132.5600
448.2415	224.6244	4	G	15	2127.0539	1064.0306
563.2685	282.1379	5	D	14	2070.0324	1035.5198
676.3525	338.6799	6	I	13	1955.0055	978.0064
1474.6896	737.8484	7	Y(+635.2737)	12	1841.9214	921.4643
1531.7110	766.3592	8	G	11	1043.5844	522.2958
1644.7951	822.9012	9	I	10	986.5629	493.7851
1758.8380	879.9226	10	N	9	873.4789	437.2431
1886.8966	943.9519	11	Q	8	759.4359	380.2216
1957.9337	979.4705	12	A	7	631.3774	316.1923
2071.0178	1036.0125	13	L	6	560.3402	280.6738
2128.0392	1064.5233	14	G	5	447.2562	224.1317
2199.0764	1100.0418	15	A	4	390.2347	195.6210
2300.1240	1150.5657	16	T	3	319.1976	160.1024
2371.1611	1186.0842	17	A	2	218.1499	109.5786
---	---	18	K	1	147.1128	74.0600

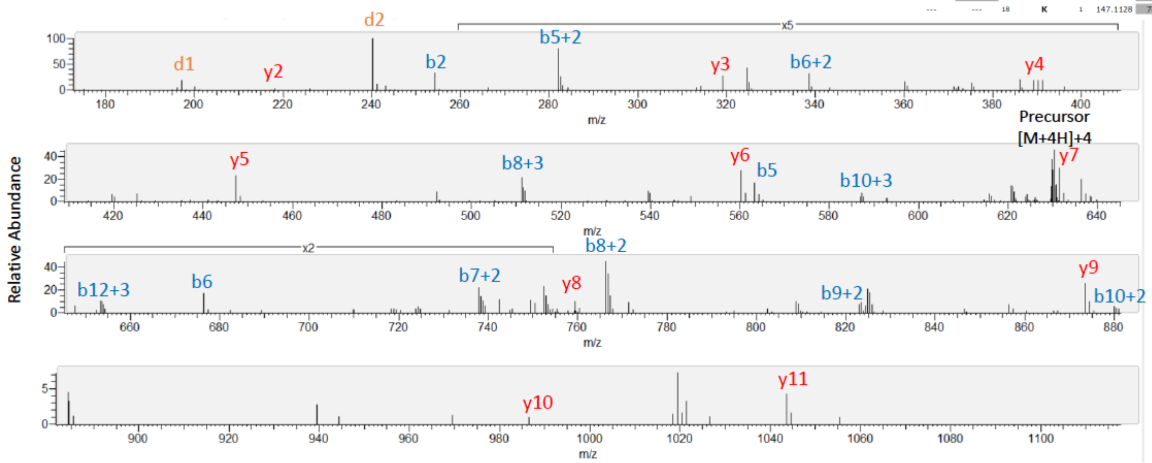

Supplement: SC-012-D0SC06623K-s001 [file SC-012-D0SC06623K-s001.pdf]
